# A Systematic Review of Negative Work Behavior: Toward an Integrated Definition

**DOI:** 10.3389/fpsyg.2021.726973

**Published:** 2021-10-27

**Authors:** Cokkie M. Verschuren, Maria Tims, Annet H. de Lange

**Affiliations:** ^1^Department of Management and Organization, School of Business and Economics, VU Amsterdam, Amsterdam, Netherlands; ^2^Department of Human Resource Management, HAN University of Applied Sciences, Nijmegen, Netherlands; ^3^Department of Psychology, Universidade da Coruna, A Coruña, Spain; ^4^Faculty of Psychology, Open University Heerlen, Heerlen, Netherlands; ^5^Faculty of Social Sciences, University of Stavanger, Stavanger, Norway; ^6^Faculty of Psychology, Norwegian University of Science and Technology (NTNU), Trondheim, Norway

**Keywords:** systematic review, negative work behavior, harm, actor types, actor roles, agreed definition

## Abstract

The objective of this systematic review was to identify the overlapping and unique aspects of the operationalizations of negative work behaviors (NWBs) to specify a new integrative definition of NWB. More specifically, we examined (1) how many operationalizations and conceptualizations of NWB can be identified, (2) whether these operationalizations can be categorized into facets, i. e., the nature of NWB, harm, actor types, and roles, with subcategories, (3) what the meaningful overlap in these operationalizations was, (4) whether the operationalizations tapped unique and meaningful elements, i.e., positive labels and dynamic processes, and (5) how the overlapping and unique elements of the operationalizations could be integrated into a new theory-based research model for NWB for future research. In the literature search based on the Prisma framework, Pubmed, PsycINFO, and Google Scholar, we identified *k* = 489 studies that met the inclusion criteria of our review. The results of these studies revealed 16 frequently studied NWB labels, e.g., bullying and aggression. Many of these could be categorized in the same way, namely, in terms of the type of behavior, type of harm, and type of actor involved in the NWB. In our new definition of NWB, we integrated the content of the overlapping and meaningful unique elements of the 16 labels.

## Introduction

Negative work behavior is a serious problem in contemporary workplaces that causes harm for involved targets (Verkuil et al., 2015; Van Steijn et al., 2019) and incurs costs for companies (Porath and Pearson, 2012) and society (Carlson et al., 2011; Nielsen et al., 2017). Negative work behavior (NWB) was defined as an “exposure to ongoing negative and unwanted behavior by superiors or colleagues” (Glambek et al., 2020 p. 509), which is harmful to employees and the organization (Cooper et al., 2004; Pearson et al., 2005; Spector and Fox, 2005; Hogh et al., 2012). Since no unitary definition of NWB exists, labor organizations such as Eurofound generally word the phenomenon as an “adverse social behavior (ASB) including all acts of physical and verbal violence and intimidation at work” (Eurofound and EU-OSHA (2014), 2014, p. 27).

Negative behavior was originally described in the schoolyard as deviance (Heineman, 1972), mobbing (Pikas, 1975), aggression, harassment, violence, and bullying (Olweus, 1978; Olweus et al., 1999). From the start, these studies regarded these behaviors not as mutually excluding each other but as complementary to each other. This means that actors are likely to use various negative behaviors interchangeably (Barboza et al., 2009; Ferrer et al., 2011). For example, a study by Pikas (1975) described that schoolchildren were “reinforcing each other in their interaction,” (Pikas, 1975, p. 3) “with humiliation, from ostracism to overt physical violence, with forms existing between these extremes, from rather benevolent ridicule to harassment of the target, verbally and physically” [1973, according to Pikas (1975), p 13–14, translated by Kirsti Lagerspetz and Kaj Berts] (Lagerspetz et al., 1982). In other words, actors use a range of behavioral possibilities in their dynamic reciprocal interaction in this schoolyard approach (Barboza et al., 2009; Ferrer et al., 2011). To sum it up, these findings suggest that a focus on the differences between the behaviors was regarded as less important than a focus on the dynamic interaction by which they occur in a complementary manner.

In the workplace, numerous labels of NWB were presented as mutually exclusive types. This number continued to grow despite signals that this diversity of conceptualizations and operationalizations was hindering progress in the field of NWB (Schat and Kelloway, 2005; Crawshaw, 2009). Meanwhile, solutions to reduce this diversity were sought in operational definitions or nested labels. For example, an operational definition of a wide range of NWBs has been used to investigate sleep problems, with the definition of NWBs including bullying, unwanted sexual attention, mobbing, physical and verbal assaults, sexual violence, verbal discrimination, verbal and sexual harassment, stalking, and assaults by internal and external actor types (Magnavita et al., 2019). As a result, the impact of different NWBs on the outcome of interest cannot be linked to one single label. Another solution is defining narrow constructs as nested within broader ones (Cropanzano et al., 2017). For example, bullying as a form of aggression (Nielsen and Einarsen, 2018) or abuse and victimization as a form of harassment (Neall and Tuckey, 2014). However, in a nested NWB structure, the data of these labels still overlap or differ at the different levels in such a hierarchy. Therefore, the problems remain as in the operational solution.

In line with the discussed concerns, researchers called for a well-developed integrated model of NWBs (Schat et al., 2006; Raver and Barling, 2007; Aquino and Thau, 2009; Hershcovis, 2011; Nielsen and Einarsen, 2018). Nevertheless, this resulted in an ongoing discussion on the possible overlap between different labels and operationalizations of NWB types (Hershcovis and Reich, 2013; Cropanzano et al., 2017), their interchangeability (Schat et al., 2006; Barling et al., 2009), and their reciprocity (Fox and Spector, 2005; Ireland, 2013). Regarding the possible overlap among NWB labels, scholars have found that this is considerable (Griffin and Lopez, 2005; Schat et al., 2006). More specifically, the overlap is found in the behavior and its repetitive patterns (Leymann, 1996; Keashly and Harvey, 2005; Lee and Brotheridge, 2006; Einarsen et al., 2011; Bayramoglu and Toksoy, 2017; Serenko, 2019), the harm that it inflicts on targets (Hershcovis, 2011), and the actors of NWBs (O'Leary-Kelly et al., 2000). To illustrate this point, scholars in earlier research found little to no difference between the various types of NWB and their relationship with psychological, e.g., mental health problems, and physical harm, e.g., physical health problems (Hershcovis, 2011). More recently, research has indicated material, e.g., loss of income, and social harm, e.g., damage of the friendship network, as other outcomes of NWB (Beus et al., 2016).

Next to the type of behavior, previous research has also paid attention to the different types of actors involved in the NWBs and defined actors mainly in three roles, namely, target, perpetrator, and witness/bystander (Neall and Tuckey, 2014). In addition to these roles, actors were characterized as four types of actors, namely, stranger, co-worker, customer, and relative (Merchant and Lundell, 2001). The relation of actors to the organization was not described in every type of NWB (Spector and Fox, 2005; De Cuyper et al., 2009), although the influence of external roles has been demonstrated through social support, flattery, or the securing of alternative resources (Fiset et al., 2017). Furthermore, definitions have referred to actors in organizational groups and population groups. Actor characteristics from these groups were regarded as important and meaningful (Kern and Grandey, 2009; Raver and Nishii, 2010; Ferrer et al., 2011; Agrawal et al., 2019).

Various studies have shown that it is not only the overlap that leads to an agreed definition, but that the meaningful unique elements also exist; thus, both should be included in the new integrative definition (Griffin and Lopez, 2005; Lim and Cortina, 2005; Schat et al., 2006; Tepper and Henle, 2011; Nielsen and Einarsen, 2018; Serenko, 2019). These overlapping and unique elements are based on important theoretical differences of constructs (Tepper and Henle, 2011). For example, the theoretical explanation in the schoolyard of complementary behavior stems from an explanation that actors show co-occurrent dynamic behavior with a wide variety of NWBs, e.g., Barboza et al. (2009). Another example of an underlying theoretical idea on behavior is to label an NWB as positive when it serves an organizational purpose such as leader bullying (Ferris et al., 2007). Furthermore, the inclusion of external actors in NWB for their influence on internal actors is another underlying theoretical idea (Fiset et al., 2017). Moreover, according to some theoretical insights, these actors are not just perpetrators and targets but consist of actor groups with various more actor roles of bystanders in a network (Salmivalli et al., 1998; Twemlow et al., 2010; Paull et al., 2012). Therefore, unique elements must be integrated considering their meaningful theoretical contribution, including elements such as external actors, several actor roles besides perpetrator and target in a group network, complementary behavior, and positive behavior.

In summary, a wide variety of NWB labels has been conceptualized in previous research to explain and measure NWBs, and scientists have been calling for a new integrative definition with a sound theoretical basis (Nielsen and Einarsen, 2018) to better operationalize NWB for future research. The present study aimed to contribute to this research field by conducting a systematic literature review of this field and developing an integrated definition of NWB. For this purpose, we successively categorized types of behavior, types of harm, types of actors, and actor roles to collect data on overlapping and unique elements of NWBs. With these data, we formed our new integrated definition on NWB.

The present study proceeded as follows. First, we present our review method, the Preferred Reporting Items for Systematic Reviews and Meta-Analyses (PRISMA) model (Moher et al., 2009) for screening and found studies with NWB operationalizations. Second, the current systematic review addresses the following research questions: (1) how many operationalizations and conceptualizations of NWB can be identified; (2) whether these operationalizations can be categorized into specific facets such as the nature of NWB with subcategories such as psychological and sociocultural NWB; (3) what the meaningful overlap was in these operationalizations; (4) whether the operationalizations tapped unique and meaningful elements, i.e., positive labels, dynamic process, and actor roles; (5) how the overlapping and unique elements of the operationalizations could be integrated into a new theory-based research model for NWB for future research. Finally, we discuss and present our new integrated model for NWB and address its limitations, implications, and pathways for future research.

## Method

### Identification of Studies

**Figure 1:**
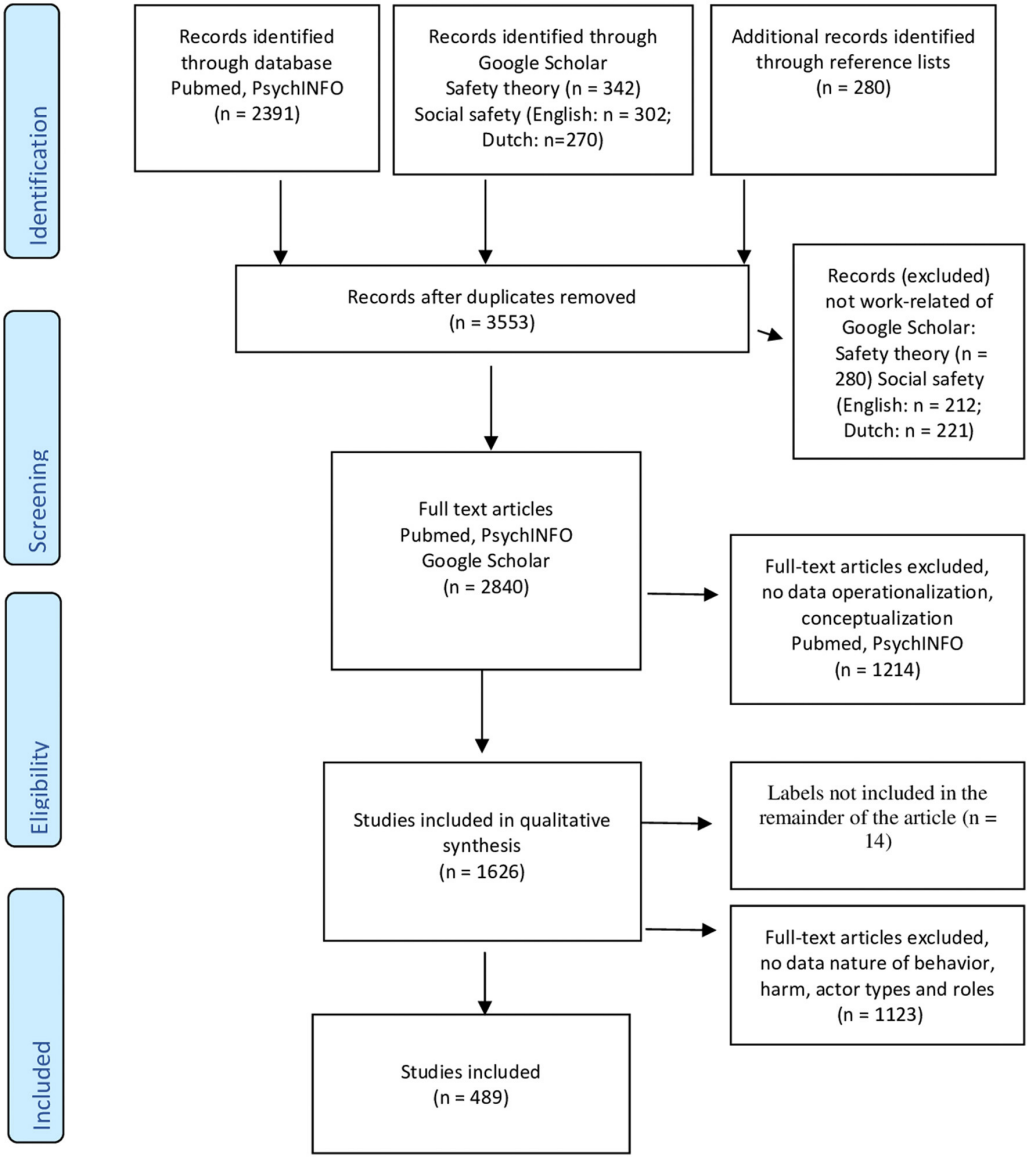
Systematic review of negative work behavior (NWB) in a Preferred Reporting Items for Systematic Reviews and Meta-Analyses (PRISMA) flow diagram.

We used the PRISMA model (Moher et al., 2009) for this review (Figure 1). In total, 3,526 articles were identified after removing the doubles. To identify relevant studies, the PsycINFO (American Psychological Association (APA), 1929) and PubMed (United States National Library of Medicine (NLM), 1996) databases were searched for peer-reviewed journals, books, reports, guidelines, dissertations, and conference papers published between 2000 and December 2020. We focused on this time frame because the field of NWB has seen large growth since 2000 due to the regulatory initiatives, policies, and research agendas in several European countries (Di Martino et al., 2003; Sloan et al., 2011). The reference lists of the full-text articles, including articles before 2000, were also searched so as to not exclude leading articles from the previous period. Since the review addressed NWB, we decided to search on seven search terms, which were “aggression and work,” “workplace bullying,” “mobbing and work,” “harassment and work,” “deviance and work,” “counterproductive work behavior,” and “social safety and work.” Social safety is an established label for NWB in sectors working with the public, clients, and pupils (Abraham et al., 2011; Ufkes and Giebels, 2013; Cheung and Yip, 2017).

Using these search terms, we identified 23 NWB labels (see Table 1). Since we obtained a large number of various labels under all search terms, we decided that further searching under more terms would be unnecessary. For example, under the search term “aggression and work” we found a total of 384 papers with 113 referring to aggression, 88 to violence, 84 to bullying, and the remaining 296 articles equally yielding other labels.

**Table 1:** Frequency of NWB labels in study titles.

**Label**	**Frequency**
Aggression	530
Bullying	170
Mobbing	59
Harassment	211
Discrimination	15
Deviance	80
CWB	30
Violence	178
Abuse	51
Abusive supervision	29
Terror	8
Injustice	19
Interpersonal conflict	13
Victimization	20
Micropolitics	6
Ostracism	30
Incivility	65
Social safety	171
**Labels not included in the remainder of the article:**	
Social undermining	4
Interpersonal mistreatment	5
Negative workplace behaviors	2
Antisocial behaviors	2
Scapegoating	1
Total qualitative analysis	1,626

*27 Papers were added in the peer review process and in Figure 1 but not in this original Table 1. In these 27 papers we found 8 bullying, 3 aggression, 3 mobbing, 2 violence, 1 victimization labels in titles*.

However, this was not the case for the term social safety, for which only 12 papers were found. Therefore, we used Google Scholar (Verstak and Acharya, 2004) to identify more studies on “safety theory” and “social safety” (English and Dutch). To limit the number of Google articles, we selected the first 30 pages of each Google search. These pages were copied to a Word file to search for the combination of the terms “safety and theory” and “social safety and work.”

### Screening

Among the identified Pubmed and PsycINFO records, we included studies on youth to provide new insights from this historically closely related NWB research field. For the Google Scholar records copied into Word, an additional step was required to screen full-text NWB articles. We screened these abstracts to include occupation-related studies only: health and safety; psychosocial safety climate; corporate social responsibility; history of safety; occupational groups, e.g., health care, social work, and education; identity and sense making related to safe work. This screening resulted in 2,813 full-text articles from Pubmed, PsycINFO, and Google Scholar.

Our next step was to screen these full-text articles for data on operationalization and conceptualization. This screening resulted in 1,599 relevant full-text articles. Among these studies, we excluded 14 studies because they examined NWBs that were found in the titles of studies less than five times. However, one of these labels, which is scapegoating, including its literature, could be categorized under victimization because of the central role of the victim in this label. The label NWB appeared to be formulated in such a general way that it could be used for the title of this study. The remaining full-text articles were screened for data on the nature of NWB, the nature of the harm inflicted, actor types, and roles. This qualitative synthesis resulted in 489 studies.

Since one author (CV) assessed the Google Scholar records on NWB and the full-text articles on the operationalizations and conceptualizations of NWB, we examined the inter-rater agreement of all authors in this screening process. Both authors screened a random sample of four safety and six social safety records (three were in English and three were in Dutch) to examine whether they could be included in the NWB literature. The same approach was taken for a sample of 10 full-text articles to examine whether they contain data on NWB operationalizations and conceptualizations. The inter-rater reliability for both samples was 96%.

### Coding Samples

In the meta-analysis, the conceptual and operational definitions of the 489 studies were selected on the following facets: A. nature of NWB; B. type of harm inflicted; C. type of actors. This qualitative information was listed and coded into four tables for further analysis. In Supplementary Table 1, we coded the natures of behaviors and occurrence patterns that were identified in concepts of NWB. The natures of NWB were A1. physical behaviors, e.g., hitting (Bernaldo-De-Quirós et al., 2015), A2. material behaviors, e.g., theft (Berry et al., 2007), A3. psychological behaviors, e.g., undermining (Nielsen and Einarsen, 2018), A4. sociocultural behaviors, e.g., NWB based on race (Johnson and Otto, 2019), and A5. digital behaviors, e.g., cyberaggression (Weatherbee, 2007). The occurrence patterns were A6. systematic behaviors, e.g., repetitive (Sexton and Brodsky, 1977; Einarsen, 2000), A7. duration (Martin and Hine, 2005; Hershcovis and Reich, 2013), A8. escalating behaviors, from mild into more severe forms (Leymann, 1996; Zapf and Gross, 2001), and A9.visibility, such as overt (Jensen et al., 2014) and covert NWB (Porath and Pearson, 2012). In Table 2, we coded positive labels of NWB. In Supplementary Table 2, we coded the natures of harm, which were B1. physical harm, e.g., headache (Tynes et al., 2013), B2. psychological harm, e.g., stress (Sarfraz et al., 2019), B3. material harm, e.g., loss of income (Lutgen-Sandvik, 2006), and B4. social harm, e.g., to the children of a victim (Ng, 2019). In Supplementary Table 3, we coded four actor types based on the model of Merchant and Lundell (2001) into C1. criminal/stranger, C2. customer/client/pupil, C3. co-worker/manager, and C4. personal relative. We combined manager and co-worker because definitions and prevalence figures name these actors separately (Health safety department US, 2016). We added to this list type C5. group of actors. In this table, we coded described actor roles in italics.

**Table 2:** Comparison of behavior in PWB and NWB labels.

CWB	**Organizational citizenship behavior (OCB)**: employee behavior that contributes to organizational performance considered including a variety of related constructs as innovative and spontaneous behavior (Katz, 1964), contextual performance (Borman and Motowidlo, 1993) and pro-social behaviors (Brief and Motowidlo, 1986), it may be person- or task related (Settoon and Mossholder, 2002)
Injustice	**Organizational justice**: is concerned with the outcomes, processes, and interactions within the organization. Distributive justice is concerned with the fairness of outcomes, procedural justice is defined as the fairness of the process that leads to decisions, and interactional justice is concerned with the quality of interactional treatment during enactment of procedures (Liljegren and Ekberg, 2009).
Incivility	**Civility:** (Ghosh et al., 2011), respect and engagement in the workplace (CREW) seeks to develop a work environment defined by civility and reduce acceptance of inappropriate behavior in the workplace (Osatuke et al., 2009)
Unsafety	**Social safety:** is protected from the likelihood of risk, harm (Edwards et al., 2013), prevent and downsize unsafety (Brewer et al., 2018)
Aggression	**Professional aggression:** socially accepted forms of aggression in the workplace, such as maintaining order, actions during strikes, and working in war zones (Kelloway et al., 2005)
Bullying	**Leader bullying**: strategically selected tactics of influence by leaders designed to convey a particular image and place targets in a submissive, powerless position whereby they are easily influenced and controlled, in order to achieve personal and/or organizational objectives (Ferris et al., 2007)
Harassment/ Discrimination	**Positive discrimination:** reversing the discrimination against the dominant majority (Webb, 1997; Noon, 2010)
Deviance	**Positive and constructive deviance**: workplace behaviors that intentionally depart from norms in honorable ways, either at an individual or organizational level (Spreitzer and Sonenshein, 2004), **constructive deviance** (Vadera et al., 2013)
Interpersonal conflict	**Task-related interpersonal conflict** entails positive behavior and outcomes in task related conditions to improve group decision making and prevent groupthink (Janis, 1972; Cosier and Schwenk, 1990; Brodbeck et al., 2002; Raver and Barling, 2007)
Micropolitics	**Productive micropolitics**: productive functions of organizational politics (micropolitics) (Neuberger, 2006).
Ostracism	**Ostracism**: powerful social influence tool to protect members and group identity, a signal to targets that their behavior needs correction, to remove deviant individuals (Hales et al., 2016)

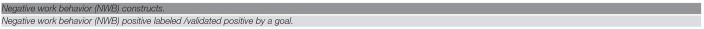

*Positive work behavior (PWB) positive alternative of primary construct NWB*.

## Results

Table 1 shows an overview of 23 labels in titles, of which 18 were included in this study. We decided to combine two types of these 18 labels due to their limited distinctions. First, harassment and discrimination were combined given that they only differ in the perceived reason for the harassment or discrimination (see also Rospenda and Richman, 2004, 2005). Second, we combined abuse and abusive supervision since the difference is mainly in terms of hierarchy, which was not coded separately in this study. This resulted in 16 constructs of NWB. Next, we presented our results with an overview of the natures of NWB and examples in each construct. We exhibited the identified categories as facets to use these sub-categories in our integrated definition.

### A. Natures of NWB

In this section, we present our results on the nature and occurrence patterns of the NWB. Nature is categorized in an occurence pattern with systematic, duration, escalation, and visibility characteristics (see Supplementary Table 1). This categorization was extracted from the way NWB has been categorized in previous studies. To mention some examples of these studies, they included physical or psychological (Santos et al., 2009) material such as organizational and personal property (Steinberg et al., 2011; Conway et al., 2016). The categories of the sociocultural and digital nature of NWB were added because of their increasing prevalence over the past 20 years. The increase in the sociocultural nature of NWB was identified in workforce diversity as a social determinant (Baron and Neuman, 1996). The increase and development of the digital NWB nature were identified as a human-machine fusion that proceeds the behavior of users at such a high rate that it exceeds the time frame for conceptualization, theory development, and investigation (Weatherbee and Kelloway, 2006). Separate from these natures of NWB, the occurrence patterns of these behaviors have been studied. These patterns reflect the intensity, persistence, or visibility of the NWB. We elaborated our results below in the facets such as A. nature and occurrence pattern of NWB, B. harm of NWB, and C. actors and actor roles in NWB.

#### A1. Physical Nature of NWB

The behavior in this category is physical and bodily in nature. This behavior is commonly defined as physical assault such as hitting, kicking, biting, scratching, pushing, spitting, or grabbing (Spector and Jex, 1998; Macdonald and Sirotich, 2005; Abraham et al., 2011; Every-Palmer et al., 2015). More severe natures of physical NWB have different names according to the label in which it is described. In harassment, it is rape (Champion, 2006), in terror, it is stalking (Every-Palmer et al., 2015), and in social safety, it is possession of weapons (Nelen et al., 2018). Besides these severe manifestations, milder forms were found such as inappropriate physical attention (Gruys et al., 2010), body language (Rominiecka, 2008), and the exclusion of rites and rituals (Anderson, 2009).

Some of the described physical natures were focused on work performance such as work violations and organizational rule-breaking. Examples of work violations are intentionally working slowly, doing work incorrectly, and withdrawal. Organizational rule-breaking involves acts against organizational rules, including production rules, service rules, and rules about sharing confidential company information. Another found physical nature related to work performance is drug and alcohol use during working hours.

#### A2. Material Nature of NWB

The common factor in the material category is behavior directed at material issues such as property, goods of persons, or the organization (Van Dam et al., 2009). This negative work behavior reflects, for example, burglary, theft, and vandalism in victimization (Engel et al., 2015), the withholding of information in incivility (Andersson and Pearson, 1999), corruption in bullying (Vickers, 2014), media portrayal in victimization (Reichert and Carpenter, 2004), and property interference in terror (Every-Palmer et al., 2015). The material nature of NWB is more frequently described in counterproductive work behavior (CWB) than in the other labels. This is probably because the primary interest of this label was behaviors thwarting work production. In this, the label distinguishes NWB toward the organization (CWBO; Ambrose et al., 2002), individuals (CWBI), or persons (CWBP; Bennett and Robinson, 2000; Neuman and Baron, 2005). Examples of CWBO are property theft and production deviance such as intentionally working slowly, doing work incorrectly, or neglecting to follow procedures (Spector et al., 2006). Examples of CWBI are knowledge withholding (Peng, 2012) or knowledge hiding (Serenko, 2019).

#### A3. Psychological Nature of NWB

Psychological behavior can be either verbal or non-verbal. Examples of verbal behavior are yelling, cursing, swearing, and shouting (Bernaldo-De-Quirós et al., 2015) or storytelling in CWBI. On the other hand, non-verbal are cues in speech (Rominiecka, 2008) or manner (Blau and Andersson, 2005). Subsequently, non-verbal acts are subdivided into acts of omission (such as ignoring something) and commission (such as a disapproving glance) (Bennett, 1983). As a result of this subclassification, the following examples fall into the subcategory psychological non-verbal acts of omission: not inviting migrants for job interviews due to discrimination (Weichselbaumer, 2017); isolation in terror (Leymann and Niedl, 1994); silent treatment in victimization (Kaukiainen et al., 2001); resistant or passive obedient behavior and loophole-seeking behavior in CWBI (Peng, 2012).

#### A4. Sociocultural Nature of NWB

The sociocultural nature of NWB is an integration of social and cultural factors (Harris, 1979). Social factors refer to society as a structure with behaviors in corporations, political organizations, hierarchies, population groups, and castes. Cultural factors refer to the learned behaviors shared by the members of society such as beliefs, values, norms, and the material products of such behaviors such as laws and religion. The way society and culture integrate with each other to function in unity is the third component, named the system (Little, 2011).

The sociocultural forms of NWB are all directed at organizational and population groups or social identity. Examples of committing NWBs that are sociocultural in nature are rude behavior on age in incivility (Kern and Grandey, 2009), discrimination on race in injustice (Raver and Nishii, 2010), aggression toward Muslims in violence (Agrawal et al., 2019), making fun of the personal life of someone in CWBP (Robinson and Bennett, 1995), or the exclusion of sociocultural workers in production groups in CWBI (Hitlan et al., 2006). The latter example shows an overlap between population and organizational groups. This overlap indicates that the integration of demographic and social identity groups into the community has an indirect relationship with NWBs in organizations (Ferrer et al., 2011).

#### A5. Digital Nature of NWB

Digital NWB is characterized by the use of emails, the internet, smartphones, and/or widespread participation on social networks, e.g., Weatherbee and Kelloway (2006) and Patchin and Hinduja (2006).

This digital medium adds new elements to NWB (McLuhan, 1994). It can be anonymous (Pettalia et al., 2013), can take place 24/7, in the very homes of individuals (Park et al., 2018), and involves a larger audience in a short time (Vandebosch et al., 2012). Although this NWB concerns the use of a technological medium, such remarkable effects have emerged in the behavior of actors in NWB that scholars have named and measured them as a separate nature, e.g., Li (2007) and Olweus (2012). Because of the anonymity, victims of digital NWB may not know who their perpetrator is or why they are targeting them. Therefore, compared with traditional forms of NWB, these victims are less likely to report and seek help (Pettalia et al., 2013). On the other hand, anonymity allows perpetrators to engage in more harmful behavior because they do not see the consequences of their behavior as they would in face-to-face encounters. Moreover, an anonymous environment does not challenge their values and emotions, which are limited by norms of morality or empathy (Pornari and Wood, 2010). In general, this less personalized information technology (IT) makes users lose connection with others (Baruch, 2005) and enables them to behave in a way that is disconnected from reality (Black et al., 2012), resulting in interpersonal conflicts (Kavitha and Bhuvaneswari, 2016) and an escalation in serious NWB with less moral limitations, reporting, and help-seeking on the part of its users (Pornari and Wood, 2010; Pettalia et al., 2013).

The acceleration of the medium requires another behavior change in all actors, which includes being alert 24/7 and missing nothing so as to react quickly even during eating and sleeping hours (Suris et al., 2014). These quick reactions are enabled by the less effort involved in spreading a message quickly and widely by cutting, pasting, and sending with the new technology. The content used in such actions, such as photos or videos, has far-reaching consequences for all actors as it can be easily spread and shared among many bystanders and often remains accessible after the initial NWB. Because of these differences in the behavior of users through the use of this medium, scholars consider the nature of digital NWB different from traditional NWB with specific conceptual and definitional aspects (Berne et al., 2013). From the point of view of users, NWB causes a different nature of behavior.

From the point of view of technology, two distinctions between the real and cyber world can be made, namely, cyber-enabled and cyber-dependent NWB (Furnell, 2002). The cyber-enabled behavior reflects actual NWBs that occur in cyberspace, such as online cyber harassment (Towns and Johnson, 2003), cyberbullying (Weatherbee, 2007), technology-facilitated violence and pornography (Henry and Powell, 2016), and fraud (Trembly, 2004). These behaviors are enabled by IT systems and could still be committed without them. Therefore, they have much overlap with traditional NWB. Cyber-dependent NWB is a direct result of IT (Furnell, 2002). These behaviors cannot be committed without IT, such as hacking (Stafford and Urbaczewski, 2004) and identity theft (Neese et al., 2005).

### A. Occurrence Patterns of NWB

#### A6. Systematic Nature of NWB

All 16 types describe NWB not as a one-time event but as a systematic, repeated, or persistent process (Loraleigh Keashly and Harvey, 2005; Bayramoglu and Toksoy, 2017; Baran Tatar and Yuksel, 2018). Since systematic NWB may lead to severe psychiatric and psychosomatic outcomes, the 6-month timeframe was originally chosen in a study by Leymann et al. to observe the occurrence pattern for its relation to the assessment of various psychiatric disorders in NWB victims (Leymann and Tallgren, 1989; Leymann and Zapf, 1990; Leymann, 1996). This observed occurrence pattern in the 16 labels varies in a range from monthly to daily (Kern and Grandey, 2009; Van Jaarsveld et al., 2010; Leiter et al., 2011; Sakurai and Jex, 2012; Sliter et al., 2012), or from never to always (Marcus et al., 2002; Ferris et al., 2008; Ruiz-Hernández et al., 2016).

#### A7. Duration

By describing NWB as a repeated process, the duration of the occurrence of incidents experienced by a victim became a part of the criterion of NWB (Einarsen and Skogstad, 1996). For the described duration in the various labels, we looked at the operationalization in the measurement instruments of NWB. In these operationalizations, we found differences in the defined duration. Among the 16 labels, a duration is defined in 1 label with 6 months (bullying; Einarsen and Raknes, 1997), 1 label with the past few months (abuse; Tepper, 2000), 1 label with past and present (victimization; Hamburger et al., 2011), 2 labels with various time frames (injustice; Colquitt, 2001, and interpersonal conflict; Spector and Jex, 1998), and 9 labels with 1 year (deviance; Bennett and Robinson, 2000; CWB; Marcus et al., 2002). The remaining 2 labels, which are micropolitics and ostracism, do not define a specific duration with the frequency of incidents but the duration of the current incident itself. By indicating the duration of an incident, the reaction patterns of actors during this incident can be observed (Hershcovis and Reich, 2013).

#### A8. Escalation Nature of NWB

Escalation is an indication that the process of NWB persistently worsens into more serious forms of NWB. Terms used for this process are spiral (Leymann, 1996; Lutgen-Sandvik, 2003; Nielsen et al., 2015), cycle (Robinson, 2008; Fisk, 2010), circle (Williams and Zadro, 2005; Chan, 2006; Martinez et al., 2008; Khoo, 2010), increased levels (Cortina and Magley, 2003; Hauge et al., 2011; Namie and Namie, 2011; Leon-Perez et al., 2015; Bashir et al., 2019), or chain of reactions (Kane et al., 2008; Webb, 2008).

#### A9. Visibility

Negative work behavior may change into more serious forms, as in escalation, and it may also change from overtly visible to covert and less visible forms (Neuman and Baron, 1998). All 16 labels include these overt and covert forms of NWB. Examples of overt forms of NWB are publicly criticizing and physical behaviors (Crawford, 1999; Kaukiainen et al., 2001). Examples of covert forms of NWB are spreading rumors, giving silent treatments, using menacing body language, and hiding knowledge, e.g., Thomas and Burk (2009) and Lewis (2006). Other terms used for overt NWB are explicit or detectible NWB, e.g., Mayhew et al. (2004) and Crawford (1999), while for covert NWB, the terms used are subtle, mild, private, or lower-level NWB, e.g., Khan et al. (2014) and Martin and Hine (2005). Examples of subtle forms of NWB are racism and discrimination because of their legalization in current society (Kern and Grandey, 2009). Scholars explained the use of these covert tactics in maximizing harm while minimizing danger to perpetrators, indicated with the “effect-danger ratio” (Björkqvist et al., 1994a,b), disguising identity (Baron and Neuman, 1996; Kaukiainen et al., 2001), or keeping an option for restoration, e.g., Skarlicki and Folger (1997). Studies on youth show that during adolescence, the overt NWB gives way to covert forms (Kern and Grandey, 2009). Apparently, covert NWB depends on the development of verbal skills and social insights (Lagerspetz et al., 1988).

In sum, all 16 constructs can be defined along with the proposed categories (A1–A5) of the different natures of NWB, although examples of some natures were listed more often in one construct than in another. For instance, the physical and psychological natures of NWB were richly defined in all constructs with many examples, while the sociocultural nature of NWB was richly filled with examples of harassment. Furthermore, we made choices for some aspects that can be classified in more than one specific nature of NWB. Examples of these are non-verbal and bodily NWB, IT-enabled or real-world NWB, and NWB in organizational and demographic groups. Another choice we made is to distinguish the occurrence patterns in these natures of NWB (A6–A9). All labels defined systematic, escalation, and visibility as meaningful occurrence patterns in NWB. The definition of duration differs, wherein 12 labels were defined duration as a timeframe for the repeated incidents experienced by the victim and 4 labels defined the duration of an incident in a general work situation with reaction patterns between actors.

### Positive Labels

Interestingly, while searching for NWBs, we also found 11 positive labels, namely, professional aggression (Kelloway et al., 2005), leader bullying (Ferris et al., 2007), positive discrimination (Noon, 2010), positive (Spreitzer and Sonenshein, 2004), and constructive (Vadera et al., 2013) deviance, task-related interpersonal conflict (Raver and Barling, 2007), ostracism as an influence tool (Hales et al., 2016), productive micropolitics (Neuberger, 2006), organizational citizenship behavior (OCB) (Brief and Motowidlo, 1986; Settoon and Mossholder, 2002), organizational justice (Liljegren and Ekberg, 2009), civility (Osatuke et al., 2009), and social safety (Edwards et al., 2013; Brewer et al., 2018) (see Table 2). Authors have remained unclear about whether these positive labels are distinct from NWB, e.g., Vadera et al. (2013), which decreases our understanding of them.

Cropanzano et al. (2017) demonstrated in their review and line of reasoning that positive and negative behaviors are psychologically distinct. With this, they build on the research of Shapiro et al. (2008) who showed, with the collected data of various constructs, that positive constructs tended to load together and were empirically separate from the NWB constructs. This suggests that positive work behavior (PWB) such as being fair and trustful is psychologically distinct from negative social behavior such as behaving unfairly or distrustful (Nicklin et al., 2011). The studies by Govier (1994) and Lewicki et al. (1998) pointed out that trust and distrust are different constructs, which means that the one is not the absence of the other, just as is the case with justice and injustice (Colquitt et al., 2010).

This implies that each of these behaviors is not bipolar but has a single continuum with a positive and negative pole for high or low scores at its opposite end (Cropanzano et al., 2017). Consequently, a low score on NWB as injustice or distrust does not automatically imply a high score on positive behavior as justice or trust (Govier, 1994; Colquitt et al., 2010). The knowledge that PWB and NWB are distinct behaviors from separate constructs with their own continuum has enabled us to compare the above-mentioned positive labels and analyze these distinct behaviors in policy and in individuals.

The comparison of behaviors in positive labels is listed in Table 2. Seven positive labels, such as positive discrimination or professional aggression, contained the same negative behavior as the primary construct. This behavior is validated as positive by a goal such as speeding up opportunities for underrepresented groups in workplaces by positive discrimination (Noon, 2010) or peacekeeping by the army (Kelly et al., 2006). Important to note here is that these goals or beneficial consequences are not equal for all workers. Some workers may experience harm or damage regardless of the positive goal (Bies and Tripp, 2005; Miao et al., 2013).

In comparing the 11 positively labeled constructs of NWB, we found 4 labels that included PWB, which are organizational justice, OCB, civility, and social safety. This behavior differs from the primary types of NWB, which are injustice, CWB, incivility, and social safety. This last type is another exception since it is labeled positive by its goal and composed of two sub-constructs, which are positive/safe and negative/unsafe behaviors. Safety has an explicit focus on the desired situation, including safety for workers, patients, clients, and the public (Young, 2012), while unsafety has to be repressed (DeJoy et al., 2010; Sijbers et al., 2014).

The distinction between NWB and PWB and the influence of goals provided meaningful knowledge for NWB policy, as research demonstrated that policies that aim to suppress negative behavior may not automatically lead to more positive behavior, just as promoting positive behavior will not automatically lead to less negative behavior. Therefore, organizations that strive to be socially safe need a focus on both, i.e., a focus on decreasing NWB and increasing PWB (DeJoy et al., 2010; Sijbers et al., 2014). To monitor the progress of this policy, PWB and NWB can be scored on their own continuums.

Another insight is how the difference between NWB and PWB can be observed in the reactions of individuals to each other. When one or more individuals exhibit NWB, they likely provoke aggressive or retaliatory responses in the other person (Hershcovis et al., 2007; Matthiesen and Einarsen, 2007; Milam et al., 2009). According to social exchange theory, in this way, a process of interpersonal reactions occurs (Mitchell et al., 2012). In this process, there is also a possibility that some individuals react with support to create an alliance with the perpetrator or target of the NWB (Heider, 1958; Priesemuth et al., 2013). Consequently, as more than two individuals are involved, their choice to respond with various constructive (PWB) or destructive (NWB) behaviors makes a substantial difference in the NWB or PWB balance in the group (Salmivalli, 1999; Aquino and Lamertz, 2004). In paragraph 3.5, we review various constructive and destructive behaviors of third parties or bystanders in this process.

### B. Natures of Harm

The harm potentially caused by NWB is part of every label definition. This may be the harm to the target, bystanders, organization, or society, such as the health of the children of the victims (Ng, 2019), the undermining of families as displaced aggression (Hoobler and Brass, 2006), and economic and moral harms (Fredericksen and McCorkle, 2013). Previous research indicated four categories of NWB harm, namely, physical, material, psychological, and social harms (Hershcovis, 2011; Beus et al., 2016). These categories with examples are listed in Supplementary Table 2. Another existing finding is that this harm may either occur immediately or be delayed (Beus et al., 2016). Although these categories were mentioned separately in this overview, they occur more often in combination, e.g., Campo and Klijn (2018) and Fredericksen and McCorkle (2013).

#### B1. Physical Nature of Harm

All types of NWB include physical harm with various somatic problems (Fredericksen and McCorkle, 2013; Hansen et al., 2014; Giorgi et al., 2015; Choi et al., 2018). More specific types of physical harm are sleep problems (Hansen et al., 2014), cardiovascular diseases (Hansen et al., 2011), and fatigue (Reknes et al., 2014b). Severe types of physical harm are injury and death (Reknes et al., 2014a; Health safety department US, 2016; Campo and Klijn, 2018).

#### B2. Material Nature of Harm

Material harm, also indicated as damage, are costs for employees, organizations, and society at large. It was also part of all NWB labels. Individual costs include loss of income (Sabbath et al., 2018), loss of employment (Lutgen-Sandvik, 2006), and damage to property (Chen and Spector, 1992). Organizational costs are sick leave (Lusinyan and Bonato, 2007), lower quality of work (Esmaeilpour et al., 2011), lower productivity (Giga et al., 2008), claims and legal fees (Bultena and Whatcott, 2008), reputational damage (Citron and Franks, 2014), turnover costs (Bambi et al., 2018), and several employer monetary costs (Chen, 2017; Sabbath et al., 2018). Societal costs are higher health care utilization (Sabbath et al., 2018), unemployment (Glambek et al., 2016), and socioeconomic impact (Reknes et al., 2019).

#### B3. Psychological Nature of Harm

All types of NWB include psychological harm. To give some examples of these outcomes, these are psychological damage, deprivation (Campo and Klijn, 2018), mental health problems, including depressive, anxiety, and PTSD symptoms (Baran Tatar and Yuksel, 2018), and other stress-related psychological complaints (Verkuil et al., 2015) such as burnout, withdrawal (Sliter et al., 2012), paranoia from digital injustice (Citron and Franks, 2014), alienation and helplessness (Riva et al., 2017), and lower self-esteem (Ferris et al., 2015).

#### B4. Social Nature of Harm

Although NWB is widely labeled as an occupational stressor, it appears to have several effects on the private life of an employee. This harm that carries over to the private sphere is also described as a spillover or crossover harm (Hoobler and Brass, 2006; Carlson et al., 2011) or generally as a social harm (Fredericksen and McCorkle, 2013). Examples of this harm are reduced marital satisfaction or work-to-family conflicts (Liu et al., 2015), depressive symptoms in family members (Crouter et al., 2006), health consequences for the children of the victims (Ng, 2019), and effects on the friendship networks (Björkqvist et al., 1994b) and intimate relationships of workers (Sperry and Duffy, 2009). In sum, all the 16 NWB labels describe elements of the four types of inflicted harm, which are physical, material, psychological, and social in nature.

### C. Actor Types

In this section, we present the results on actor types and roles. The actor type is the type of actor in relation to the organization (Merchant and Lundell, 2001), while the actor role refers to the role of an actor, such as supporter or perpetrator (Huitsing and Veenstra, 2012). The 16 constructs with four categories of actor types are listed in Supplementary Table 3. The roles are shown in italics. Added to this list is a fifth category “group,” a unit of actors, that also appeared to be important during the systematic analysis of the literature.

#### C1. Stranger

Strangers were included as actors in 12 of the 16 NWB definitions. We found no definition of strangers in the NWB types mobbing, CWB, interpersonal conflict, and ostracism. The descriptions of strangers varied from third parties to indirect actors such as an audience or the public. Their common feature is a position outside the organization, but they differ in roles such as criminal, visitor, or co-user in traffic. Two unique aspects were found here. First, actors in bullying are originally described within the organizational boundaries, but these boundaries are shifted outward by the digital medium in cyberbullying (Kavitha and Bhuvaneswari, 2016). Second, in procedural harassment, the judicial system is described as an outside actor (Clemente et al., 2019).

#### C2. Co-worker/Manager

All 16 types of NWB define actors as organizational members, such as workers or staff. They have been defined hierarchically as divisions between managers and subordinates, whose relationship is vertical, and between co-workers, whose relationship is horizontal (Parzefall and Salin, 2010). They may be current or former workers (Baron and Neuman, 1996). Over the past 20 years, these employees have been defined in the roles of perpetrators, targets, and bystanders as witnesses (Neall and Tuckey, 2014). Some NWB labels are less specific about actors and just mention the workplace such as in deviance (Bashir et al., 2019), working life in mobbing and terror (Leymann and Zapf, 1990), or insiders in abuse (Grandey et al., 2007).

#### C3. Customers

In 15 of the 16 types of NWB, actors are defined as parties in the business. They are customers or more specifically defined as clients, patients, pupils, students, the public, passengers, or prisoners. These may be perpetrators, such as in aggression (Dormann and Zapf, 2004), sexual harassment (Yagil, 2008), and incivility (Sliter et al., 2010; Wilson and Holmvall, 2013; Schilpzand et al., 2016), targets, such as in service sabotage (Harris and Ogbonna, 2006), or supporters, such as in ostracism (Fiset et al., 2017). Moreover, these roles may alternate in a reciprocal process (Ireland, 2013). We found no definition of customer parties in mobbing.

#### C4. Relatives

Relatives come from the network of workers or customers, such as family or friends. We found no definition of relatives in 6 of the 16 types of NWB, including deviance, CWB, injustice, interpersonal conflict, micropolitics, and ostracism. With that, these labels limit themselves to organization members only. However, in many professions, this boundary between internal and external actors is not so sharply drawn. In this study, external actors appeared to play a key role in the interactions of employees with each other, customers, and their relations (Van Dierendonck and Mevissen, 2002). In this interaction, relatives may take escalating or conciliatory roles in NWB (Levine et al., 2011). Therefore, it is not surprising that NWB labels with considerable attention to this actor type, such as aggression, violence, and social safety, were found in our reviewed studies. Some examples of these occupations are the healthcare sector, where relatives intervene in the provided care of their family member/friend (Muzembo et al., 2015; Maran et al., 2017; Sun et al., 2017; Cheung et al., 2018; Cannavò et al., 2019), and the public sector, where relatives are clients too (Waddington, 2004; Ufkes and Giebels, 2013).

#### C5. Group

Every type of NWB defines the group as a unit of actors. This group may be restricted to the organization (Hoel et al., 2001; De Cuyper et al., 2009) or to a part of the community (Campo and Klijn, 2018). A combination of both is when actors are participants interacting within (intra) and between (inter) groups (O'Boyle et al., 2011). These dynamics evolve in the shared characteristics of employee or customer groups (Brees et al., 2013; McLindon et al., 2018). The importance of knowledge about group dynamics is demonstrated by the research of Glomb and Liao (2003), which showed that 46% of the NWB is determined by group and 10% is individually determined.

### C. Actor Roles

Since all labels include actors in a group, their various roles may influence NWB in different ways (Merton, 1957; Callero, 1994). The most studied roles in these groups are target and perpetrator (dyad) or extended with a bystander (triad) (Neall and Tuckey, 2014; Pinto, 2014; Maran et al., 2018). This extension in the triad changes the process by alliances of persons (Heider, 1958). For instance, an alliance between perpetrator and bystander increases the negativity, whereas an alliance between target and bystander decreases the negativity (Aquino and Lamertz, 2004). In addition to the role of perpetrator and victim, 13 bystander roles with constructive and destructive behaviors in such alliances were found, such as assistants, reinforces, endorsers, outsiders, defenders, supporters (Salmivalli, 1999), bully bystanders, puppet-masters (instigator), victim bystanders, avoidant bystanders, abdicating bystanders, sham-bystanders, and helpful bystanders (Twemlow et al., 2010). To illustrate this, the assistants of the perpetrator offer positive feedback as the audience or incite by laughing or making encouraging gestures (reinforces and/or endorsers). Outsiders or abdicating and avoidant bystanders take no sides but have important influence (Twemlow et al., 2010). The avoiding, neglecting, or laisser-faire role of NWB silently approves of the NWB (Salin and Hoel, 2010; Ågotnes et al., 2018). In addition, some bystanders disapprove of the NWB by supporting the target and trying to make the others stop, i.e., defenders, supporters, and helpful bystanders (Salmivalli, 1999; Twemlow et al., 2010). According to these findings, bystanders play an important role in the balance of NWB and PWB in groups.

#### C6. Constructive and C7. Destructive Actor Roles

Based on the works of Twemlow et al. (2010) and Salmivalli (1999), the 13 potential bystander roles were categorized into four clusters, which are active or passive and constructive or destructive (Paull et al., 2012). The four active constructive roles were defending, sympathizing, defusing, and intervening, while the one passive constructive role was empathizing. The four active destructive roles were facilitating, collaborating, manipulating, and instigating. The four passive destructive roles were submitting, succumbing, avoiding, and abdicating. Although the study of Paull et al. (2012) chose to use the label bullying, their operational definition is often broader, including the full range of behaviors described in facet A1–5 in this study.

Another point is that, from the dynamic perspective, actors are not fixed in their roles but may switch between roles; for instance, a change from a perpetrator into a target, taking both roles as “bully/victim” (Ireland, 2013). Another example is a switch from a bystander into a target because of emotional responses (succumbing bystander) or by offering oneself as an alternative target (submitting bystander) (Omari, 2010). In this way, actors can take different roles, move between them, or simultaneously take more roles, thus changing the group dynamics of NWB (Oh and Hazler, 2009).

These studies show a more comprehensive picture of NWB than just the alliance between perpetrator and target. This alliance is extended by a wide variety of relationships between actors influencing the balance of NWB with constructive or destructive behaviors (Heider, 1958; Twemlow et al., 2010; Paull et al., 2012; Fiset et al., 2017). That is why these studies rather speak of social networks of actors instead of dyads or triads (Volk et al., 2017; Herkama and Salmivalli, 2018), as evidenced by researchers using network theory to analyze roles in NWB (Veenstra et al., 2013).

In sum, all 16 constructs overlapped in the definition of actor roles since they contained similar elements of actor types as co-workers/managers and a group of actors. A full overlap was found in the definition of three actor roles: each label defined the target, perpetrator, and bystander as a witness. A large overlap was found in the other three actor definitions in the 16 constructs: criminal/stranger (identified in 14 labels); customers (identified in 15 labels); relatives (identified in 10 labels). Unique and meaningful actor roles were the 13 active or passive roles of bystanders with constructive and destructive influences on the balance of NWB. These bystander roles are more complex to study than roles in a dyad. Consequently, the network theory is an emerging method to research more actor roles in a group (see Table 3).

**Table 3:** Facets, categories, overlap and unique elements in NWB operationalizations and conceptualizations.

1. We identified 489 studies with data on definition categories in NWB operationalizations and conceptualizations.		
2. Categorized into facets:	3. Meaningful overlap of defining elements:	4. Tapped unique and meaningful elements:
**A. Nature of NWB:**		Work violations
A1 physical	A1-5: 16 types	Organizational rulebreaking
A2 psychological		Drug / alcohol use at work
A3 material		
A4 sociocultural		
A5 digital		
**A. Occurrence pattern**		
A6. systematic	All 16 types	
A7. duration		
A8. escalation		
A9. visibility		
**PWB**		Positive behavior: in 4 types
**B. Harm**		
B1. physical B2. psychological B3. material B4. social	B1-4: 16 types	
**C. Actor type**		
C1. criminal/stranger C2. co-worker/manager C3. customer C4. relatives C5. group	1: 14 types 2: 16 types 3: 15 types 4: 10 types 5: 16 types	1: for 2 types 2: - 3: for 1 type 4: for 6 types 5: -
**C. Actor roles (15)**		
perpetrator, target, bystander as witness C6. Constructive roles (5)active constructive: defending, sympathizing, defusing, interveningpassive constructive: empathizing. C7. Destructive roles (8)active destructive: facilitating, collaborating, manipulating, instigatingpassive destructive: submitting, succumbing, avoiding, abdicating.	All 16 types C6/-C7: 3 types	For 13 types, dynamic interaction, network methodto study actor roles

## Discussion

This study aimed to provide a systematic review of earlier research examining NWB and to determine whether an integrated operationalization of NWB would be possible after examining the contents of the studies found. We arrived at the following results as an answer to our preconceived five questions for this study. To answer the first research question that stated, “How many operationalizations of NWB can be identified?” we identified 16 different operationalizations of NWB in the literature. We further investigated whether the negative work behaviors within these 16 constructs could be categorized into specific facet areas, namely, type of harm, type of actor, and actor roles (assessing research question 2: “Can the operationalizations found in the reviewed studies be categorized into specific facet areas?”). Interestingly, we found that all operationalizations of NWB show overlap in these three categories, namely, the behavioral category, the type of harm category, and actor roles (addressing research question 3: “What is the meaningful overlap in the operationalizations?”). This means that all NWB constructs share similar characteristics that define the behaviors and the type of harm, and each construct defines actor roles as perpetrator, target, and a bystander as a witness. We also found a full overlap on the actor types of co-workers/managers and groups, but less so for the actor types of strangers, customers, and relatives. Taken together, this means that none of the labels, for example, only focuses on physical behaviors, material harm, or the perpetrator.

Finally, next to the overlapping elements of NWB, we also encountered unique elements in the operationalizations of NWB (addressing research question 4: “Do the operationalizations used in the reviewed studies tap unique and meaningful elements?”). Specifically, in the category of actor types, we noticed that several additional actors were included in some operationalizations but not in others. For example, within the category of actor types, strangers, customers, and relatives were included in addition to the actor type of the co-worker (Merchant and Lundell, 2001) and a group of actors. Our choice to include all actor types is in line with the choice of many recent studies (Cheung et al., 2017; Baran Tatar and Yuksel, 2018; Gilardi et al., 2019; Maran et al., 2019). This is not surprising, since these actor types are considered important in sectors such as the health service (Öztunç, 2006; Muzembo et al., 2015; Cheung and Yip, 2017; Yenealem et al., 2019), education (Ferrer et al., 2011), and other public sectors such as hospitality and travel (Bentley et al., 2009).

Considering NWB as a group phenomenon implied perspectives to broaden our focus from a purely interpersonal approach to a network approach. The perpetrator and target do not determine the balance of NWB, but the shared hierarchy, norms, and behaviors of the entire actor group (Forsberg and Thornberg, 2016). Mapping this dynamic process with 13 destructive and constructive actor roles provides the NWB research field with new opportunities for analysis and intervention (Twemlow et al., 2010; Paull et al., 2012; Herkama and Salmivalli, 2018). In these dynamics, repetition and escalation offer a new perspective compared with the previous labels because they provide information about the ties and intensities between actors (Veenstra et al., 2013). These different observations about actors, their ties, and roles in NWB are increasingly supported by analyses from network theory (Volk et al., 2017).

A specific point that was noted in this study was that PWB is psychologically different from NWB (Shapiro et al., 2008; Cropanzano et al., 2017), and yet it is essential to include this behavior in an integrated NWB operationalization. The reason for this is that PWB gives substance to constructive bystander roles as defenders, sympathizers, defusing actors, interveners, and empathizers in a group (Paull et al., 2012). More specifically, this concerns involuntary groups in which the actors are obliged to each other, such as work teams and classrooms (Juvonen and Galván, 2008). Evaluations in the schoolyard show how promising interventions aimed at increasing constructive roles in these groups are for reducing NWB (Escartin, 2016; Raveel and Schoenmakers, 2019). Also in work situations, interventions that not only suppress negative behavior but encourage positive behavior at the same time are positively evaluated (Gamble Blakey et al., 2019). Since constructive bystander roles create a PWB balance in a group, we cannot exclude this meaningful element from an integrated operationalization (Juvonen and Galván, 2008; Brechwald and Prinstein, 2011).

An advantage of this study is that we have been able to organize our NWB data within categories. However, in real life, this distinction is not so sharp, and the co-occurrence of these behaviors is more norm than exception (Richman et al., 1999; O'Connor et al., 2004; Rospenda et al., 2009). In fact, we could rather speak of co-occurrent and sequential behavior from a broad collection of NWBs. In a co-occurrence perspective, multiple natures of NWB occur simultaneously, such as yelling and hitting (psychological and physical) or exclusion based on gender (psychological and social). In a temporal sequence, actors are likely to exhibit one type of NWB that triggers another (Gruys and Sackett, 2003), such as alcohol use evolving into physical abuse (McFarlin et al., 2001). As we discussed above, these behaviors are not limited to one person or one action. Therefore, we should rather speak of co-current and sequential NWBs between joint actors (Lim and Cortina, 2005). Consequently, we should regard the various NWBs as complementary in the communication style of actors (Barboza et al., 2009; Ferrer et al., 2011). In sum, the following integrated definition can be derived from the overlapping and the unique elements of earlier NWB operationalizations examined in this review, namely:

Negative work behaviors consist of physical, material, psychological, sociocultural, and/or digital behaviors that may inflict physical, psychological, material, and social harm for individuals, organizations, and society in a direct or delayed way. Negative work behavior is conducted by actors operating in dyads, triads, or within a networking group. These actors may be a criminal/stranger, customer/client/pupil, co-worker/manager, or personal relative during the performance of work. Depending on the process over time, these actors may take different constructive and destructive roles in a group network with targets and perpetrators. These roles influence the balance of NWB in this group. The repetition of the behavior escalating into more severe acts at one person over a period of 4–6 months makes this target no longer able to defend himself.

### Theoretical Implications

The vast majority of NWB research concentrates on harms, perpetrators, targets, and bystanders as witnesses, leaving other meaningful elements from labels barely touched (Branch et al., 2013; Neall and Tuckey, 2014). Since these NWB elements overlap completely, this implies that we are investigating the same phenomenon under different labels. This problem, also known as construct proliferation, indicates that successive labels are too similar to previous ones and therefore lack discriminant validity (Shaffer et al., 2016). This problem can be solved by an integrated operationalization of NWB. Consequently, inadequate solutions as a temporary operational definition or nested construction become redundant.

An important implication of our work is related to the measurement of NWBs. Such a measure should integrate complementary behaviors by various actors in a dynamic group network, which requires new measurement techniques. In recent years, these new techniques have been applied in research on youth in environmental systems (Veenstra et al., 2013). Although, so far, this research has focused on the graphical presentations of bullying networks of (reciprocal) in/outgroups and roles in classrooms, this may be a promising pathway for research in workplaces (Huitsing and Veenstra, 2012). In the network approach, the duration of observation makes it possible to register different natures of harm in time. For example, at time one, we may observe that the perpetrator exhibits NWB toward the target, resulting in physical or psychological harm to this target (Verkuil et al., 2015; Van Steijn et al., 2019) and bystanders (Mayhew et al., 2004; Lutgen-Sandvik, 2006). At time two, this experience of the target spills over to irritated negative behavior toward his partner, causing social harm (Ng, 2019). At time three, two team bystanders take a destructive role toward the perpetrator, resulting in a loss of status and social damage for this person (Kircher et al., 2011). At time four, the NWB causes the loss of team-sprit, production, and absenteeism among team members, subsequently causing financial harm for the team (Harris and Ogbonna, 2006; Nielsen and Einarsen, 2018). At time five, HRM solves the problem through an intervention, e.g., a mediator, lawyer, organizational advice, or exit, causing financial harm to the organization (Porath and Pearson, 2012). This means that NWB from various actors in a network causes different harm at different times, whether direct or delayed in time (Beus et al., 2016).

### Practical Implications

An integrated NWB definition connects to the social debate on actual themes. These themes, such as Black Lives Matter, #MeToo, or Doxing, include no mutually excluding behaviors but a wide range of complementary and co-current physical, psychological, material, sociocultural, and digital NWBs (Snyder et al., 2017; Holroyd-Leduc and Straus, 2018; Chen et al., 2019; West et al., 2019). If the debate about mutually exclusive labels among researchers continues, the scientific community may miss the connection with the social debate.

Another practical implication is that bystanders are easier to influence by HRM than the dyad of perpetrator and target (Herkama and Salmivalli, 2018). Indeed, HRM hardly succeeds in protecting the victim and correcting the perpetrator in the escalated and visible phase of NWB. In this phase, the perpetrator may already have organized support from management or colleagues, resulting in little support from HRM for the victim (Namie, 2003; Namie and Namie, 2009). Therefore, this escalated phase often leaves arbitration as the only option left (Keashly and Nowell, 2003). In the option with bystanders, HRM can achieve more, as they are easier to influence than the dyad (Herkama and Salmivalli, 2018). These bystanders of NWB are caught in a social dilemma. On the one hand, they understand that this behavior is wrong and would like to stop it. On the other hand, they strive to secure their own status and safety in the group (Salmivalli, 2010; Forsberg et al., 2014). Therefore, it is easier for HRM to support bystanders with skills to play a constructive role in this process (Salmivalli, 1999). Moreover, over time, this bystander intervention creates a safe group atmosphere, including the dyad (Salmivalli et al., 1998).

In describing the digital nature of NWB, we realized that this NWB may be more specific to actors in the younger generation, especially the youth born between 1980 and 1995 in the world of technology entering the labor market (Schäffer, 2012). These young workers are more comfortable with digital communication (Bencsik et al., 2016). However, simultaneously, the use of ICT is changing labor markets in such a way that the production, trade, sales, and service facilitated by online platforms dominate over traditional markets (Rabenu, 2021). This development has far-reaching consequences for workers with, on the one hand, a large group of low-skilled workers such as cab drivers and production and warehouse workers, and, on the other hand, a small group of high-skilled workers with ICT and social skills (European Commission, 2019). For instance, labor services mediated by platforms is the main work activity for only 1.4% of the working-age population, and the average age of platform workers is just below 34 years (European Commission, 2019). Further research is needed to analyze whether actors of different generations in these labor markets produce a different nature of NWB by using a different medium, e.g., physical and digital.

### Limitations

There are several limitations of the current review study we would like to pay attention to. Firstly, although our focus was primarily on NWB, we devoted an entire paragraph to PWB. This is because the paradox of NWB policy is its interventions focus on achieving PWB. This study provided important insights into this policy, wherein PWB differs from NWB and a majority of constructive bystander roles in a group are necessary for the PWB balance. Because this study did not focus on the nature of PWB, future research is needed to see if and how PWB can be included in an integrated operationalization of NWB.

Another limitation of this study is our choice to focus on three elements in each NWB definition. As a result, other elements regarded as important by some authors in NWB labels such as intent, e.g., Namie and Namie (2011), and power, e.g., Einarsen and Skogstad (1996), were not reviewed. For instance, intent has been variously conceptualized either as a necessary feature (Neuman and Baron, 2005; Spector and Fox, 2005; Hershcovis et al., 2007; Jex and Bayne, 2017), or not conceptualized explicitly as a defining element (Notelaers et al., 2018). Despite these various conceptualizations, these elements could be potentially important in an integrated conceptualization of NWB and deserve further investigation.

The next limitation is the underrepresentation of research from non-Western countries on NWB (Giorgi, 2010), which is reflected in our study. Therefore, cultural differences should be considered when interpreting the results of this study. This is because culture can play a crucial role in the operationalization of NWB. For example, in South Korea, NWB terms imply a group action that is deeply rooted in the collectivist national culture with value for conformity. Strong social ties among in-group members simultaneously imply strong discrimination against the out-group. This makes it easy for anyone who is different from the group, or a threat to the advantage of the group, to be targeted by NWB from within the group. These targeted individuals are discouraged from contradicting the group and conforming according to the core social value in Korean society (Seo et al., 2012). Future research is needed to study operationalizations from more cultures allowing other valuable insights to allowing valuable new insights from future research to broaden the knowledge about the phenomenon NWB.

A fourth limitation is that this study collected data that are not necessarily a reflection of the predominant theories from which they originate. For example, studies from individual, dyadic, group, industry, or community perspectives are tied to one or more organizational levels of NWB (Klein et al., 1994). Actors are part of a dyad, group, industry, and society (Saam, 2010). In fact, no element in this integration is level-free. These multi-level elements are not explicitly discussed in this study, although these levels, can be recognized as for instance in actor types outside the organization (Merchant and Lundell, 2001). More specifically, the influence of demographic, population and organization groups in the sociocultural nature of NWB (Lewis and Gunn, 2007; Kern and Grandey, 2009; Žukauskas and Vveinhardt, 2013; Agrawal et al., 2019), and the dyad as part of the group with bystanders (Fiset et al., 2017). A multi-level approach is necessary because it renders more validity and reliability in NWB research (Podsakoff et al., 2003; Branch et al., 2013). Therefore, future research is needed to extend this integrated conceptualization with a multi-level approach.

Finally, with a combination of all labels into one single integrated operationalization of NWB, we build on the accumulated science of NWB. This means that the original valuable elements such as the powerless role that targets eventually end up in are extended with valuable elements such as the influence of various positive and negative bystander behaviors in the balance of NWB.

## Conclusion

This study aimed to review the existing literature on NWB and develop an integrated definition of NWB. The literature review revealed that the 16 frequently studied NWB labels overlap to such an extent that they can be reduced by a comprehensive integrated model. This provided a definition that should help researchers to overcome construct proliferation and related methodological and practical‘ problems. By integrating the unique meaningful elements into this NWB definition, valuable new pathways for research and intervention were provided. The focus of this study on the key characteristics of behavior, harm, actor types, and roles brought limitations to elements such as PWB, moderators, and the multi-level approach of NWB. These elements need further investigation for integration.

## Data Availability Statement

The original contributions presented in the study are included in the article/Supplementary Material, further inquiries can be directed to the corresponding author/s.

## Author Contributions

CV: study conceptualization, data collection, and drafting of the manuscript. MT: continuous feedback, critical revisions during drafting, supervision, and substantial revision suggestions. AL: final editing and substantial revisions suggestions. MT and AL: independent rating of a sample of 10 abstracts and a sample of 10 articles for inclusion criteria and approval of the final completion of the manuscript. All authors contributed to the article and approved the submitted version.

## Conflict of Interest

The authors declare that the research was conducted in the absence of any commercial or financial relationships that could be construed as a potential conflict of interest.

## Publisher's Note

All claims expressed in this article are solely those of the authors and do not necessarily represent those of their affiliated organizations, or those of the publisher, the editors and the reviewers. Any product that may be evaluated in this article, or claim that may be made by its manufacturer, is not guaranteed or endorsed by the publisher.
