# Supplementary material for: A Systematic Review of Negative Work Behavior: Toward an Integrated Definition
Source: Front Psychol. 2021 Oct 27;12:726973. doi: 10.3389/fpsyg.2021.726973 (PMC8578924; doi:10.3389/fpsyg.2021.726973)
Supplement: Supplementary file 1 [file Table_1.docx]

**Supplementary**

**Table 1 |** A. Nature of NWB in labels derived from study titles

| **Construct NWB** | **Nature: A1. Physical A2. Material A3. Psychological A4. Social A5. Digital**  **Occurrence patterns: A6. Systematic A7. Duration A8. Escalating A9. Visibility Nested labels: in italics** |
| --- | --- |
| Aggression A  A  A  A  A  A  A  A  A | 1. personal attacks, painful messages and evil responses (Anderson and Bushman, 2002) 2. interpersonal aggression towards materials (Weatherbee and Kelloway, 2006) 3. nonverbal (Underwood 2002), hostile workplace behaviors (Keashly and Jagatic 2010), conceptualized as *abusive supervision, incivility, bullying/mobbing, harassment, victimization, interpersonal deviance, emotional abuse, ostracism, social undermining* (Nielsen and Einarsen 2018), attacking a person circuitously by remaining unidentified (Björkqvist, Österman, and Lagerspetz, 1994), social exclusion (Anderson and Bushman, 2002) 4. on gender (Maran et al. 2019), on racial and ethnic minorities (Holmes and Smith 2012) 5. cyber-aggression (Weatherbee 2007) 6. pattern of regular reacting behavior (Lee and Brotheridge 2006) 7. ﻿﻿during the past year, ranging from never, annually, quarterly, monthly, weekly to daily (Healthcare-worker’s Aggressive Behaviour Scale-Users: HABS-U, Ruiz-Hernández et al. 2016), current situation, ranging from extremely, to somewhat, neither-nor uncharacteristic, somewhat, extremely characteristic of me (Aggression Questionnaire: AQBP, Buss and Perry 1992) 8. gradually escalating process to more open and aggressive (Neuman and Baron, 1998), ﻿low level of aggression in the workplace can escalate into more intense forms of aggression (Andersson and Pearson 1999) 9. development of social and verbal skills that are more sophisticated strategies to harm a target person without being identified: named indirect aggression (Lagerspetz, Björkqvist, and Peltonen 1988), level of physical aggression decreases drastically during adolescence replaced by verbal and covert aggression to maximize effect and minimize risk ‘effect/danger ratio’(Björkqvist, Österman, and Lagerspetz 1994), make a distinction in covert-overt aggression (Buss 1995), workers exhibit direct covert aggression, such as sabotage, at a supervisor in an attempt to restore a perceived inequitable situation (e.g., Skarlicki and Folger 1997) |
| Bullying A  A  A  A  A  A  A  A  A | 1. ﻿battery such as grabbing someone's arm, pushing or punching a person, or striking a victim with an object and homicide in the worst form (Namie 2003)all types of mistreatment at work (namie en namie 2009) forms of aggression such as badgering (Solberg and olwues 2003) 2. theft of resources (Conway et al. 2016), corruption (Vickers 2014) 3. psychological disrespect and intolerance of human values (Margaretha Strandmark and Rahm 2014) 4. being rejected and expelled (Strandmark and Hallberg 2007), on ethnicity, migrant (Daly et al. 2018), on age, socio economic status (Lange et al. 2019) on ﻿social inequality and gender (Klein 2006) on gender, race and class (Sobre-Denton 2012), on minorities (Lewis and Gunn 2007), on social vulnerable (Tsuno et al. 2015),﻿ no definitive list of bullying behavior﻿, involves divers forms of social exclusion (Nielsen et al. 2015),﻿ means harassing, offending, socially excluding someone (Einarsen, Hoel, Zapf, and Cooper, 2003) 5. cyber bullying (Weatherbee and Kelloway, 2006), aggressive intentional, electronic forms of contact (Smith 2009) 6. repeatedly and over time against a victim (Smith 2009), occurs regularly and across a certain time period (Einarsen, 2000), repeated negative acts (Nielsen, Tangen, Idsoe, Matthiesen, and Magerøy, 2015) 7. ﻿during the past 6 months, ranging from never to almost never, once a week, more (Negative Acts Questionnaire: NAQ, Einarsen and Raknes 1997) 8. Spiral of Conflict; at least four phases from conflict to stigma (Leymann 1996), gradually evolving process (Hauge, Skogstad, and Einarsen 2009), continuum of negative interpersonal behavior, from mild to severe (Namie and Namie 2011) 9. ﻿methods of bullying: overt visible physical and mental nature, and covert, the less detectible, subtle nature (Crawford 1999), ﻿subtle or covert acts (Lewis 2006; Quine 2001) |
| Mobbing A  A  A  A  A  A  A  A  A | 1. physical violence (Zapf, Knorz, and Kulla 1996; Wu, Lyons, and Leong 2015; Leymann 1996; Hubert and Furda 1996) 2. prohibit work (Hubert 2002), giving a person too few or overly simple tasks (Vartia 1993), negatively affecting someone’s work tasks (Hutchinson 2013) 3. slander, threatening or criticizing, insinuations about the victim’s mental health (Vartia 2001), aggression as communication blockage, being discredited, in horizontal, ascending, descending harassment (da Silva João and Saldanha Portelada 2019), verbal aggression such as spreading rumors (Zapf, Knorz, and Kulla 1996) 4. on socio-demographic characteristics, such as age, gender, marital status (Žukauskas and Vveinhardt 2013), on workers with ﻿indefinite contract situations, union members (González Trijueque and Graña Gómez 2010), social isolation (Vartia 1993), sexual harassment, discrimination (Hubert 2002), social aggression and harassing, offending, socially excluding someone (Hutchinson 2013), attacking the victim's private life (Zapf, Knorz, and Kulla 1996) 5. cyber-mobbing (Fawzi 2009), Blackmail (da Silva João and Saldanha Portelada 2019) 6. ﻿systematically and frequently repeatedly and regularly and over a long period (Friedenberg 2008), repeatedly targeted (Baran Tatar and Yuksel 2018) 7. past year, ranging from never/ rarely to once a month, once a week, daily (Leidse Mobbing Schaal: LEMS-II, Hubert and Furda 1996; Hubert 2002); last 6 months, ranging from never to rarely, at least once every month, at least once every week, at least once every day (Revised version of the Negative Acts Questionnaire: NAQ-R, Einarsen, Hoel, and Notelaers 2009) 8. Cycle of Escalation (Leymann 1996), phases of mobbing (Khoo 2010) 9. covert mobbing in the public sector (Shallcross, Sheehan, and Ramsay 2008), overt vertical mobbing is overt power where it has lost legitimation and has become coercion (Kerckhove 1993) |
| Harassment A  /Discrimination A  A  A  A  A  A  A  A | 1. fondling, rape (Champion 2006) 2. physical display of sexually explicit materials, intending to insult and derogate (Konik and Cortina 2008), ‘Quid pro quo’ involving bribery, or conditions of employment (EEOC 2011) 3. verbal harassment ﻿is behavior expressed through words, tone or manner (Neuman and Baron, 1998), symbolic behavior such as sexist jokes, sexual epithets (Konik and Cortina 2008) 4. ﻿on age (Perry and Finkelstein 1999), on minority group (Schneider, Wesselmann, and DeSouza 2017), on disability, religion, national origin, sexual orientation (Raver and Nishii 2010), on various identity group characteristics: race, gender, sex (Johnson and Otto, 2019), umbrella term encompassing all specific forms only differ in the perceived reason (sex, race, age; Rospenda, Richman, and Shannon 2009), harms members of socially marginalized groups (Feagin and McKinney 2005) 5. cyber (sexual) harassment (Towns and Johnson 2003), e-mail harassment (Baruch 2005) 6. repeated and persistent (Sexton and Brodsky 1977), harms systematically (Feagin and McKinney 2005) 7. ﻿past year, ranging from ﻿never, once or twice, sometimes, often, many times (Gender Experiences Questionnaire: SEQ, Leskinen and Cortina 2014),﻿ past 24 months on a scale ranging from never to almost always (Ethnic Harassment Experiences (EHE) scale, Schneider, Hitlan, and Radhakrishnan 2000) 8. unfolds within-persons taking several months or longer to become apparent (Neall and Tuckey 2014), ﻿unfolds across time, as do victims’ responses (Fitzgerald, Swan, and Fischer 1995), typical escalating process (Zapf and Gross, 2001) 9. ﻿covert, subtle forms of interpersonal mistreatment (“microaggressions”) of discrimination, racism (Kern and Grandey 2009; Konik and Cortina 2008), ﻿direct overt: ﻿insulting comments, showering abuse on the person, publicly criticizing the other person’s appearance, threatening fellow employees, blaming an employee unfairly/ ﻿covert: negative glances and gestures, do-not-speak-to-me behavior, interrupting someone on purpose, imitating an employee’s style of walk, expressions or gestures in a derogative manner, refusing to listen to the other person, insinuating that the other person has mental problems (e.g., Kaukiainen et al. 2001) |
| Deviance A  A  A  A  A  A  A  A  A | 1. aggressive physical acts (Robinson and Bennett, 2000), violent acts (Berry, Ones, and Sackett 2007), physical assault (Stewart et al. 2009), assault, physical violence on body (Ménard, Brunet, and Savoie 2011) 2. rule breaking acts against organizational property/ assets and production (Hollinger, 1986), service sabotage (Harris and Ogbonna, 2002), theft from co-workers (Berry, Ones, and Sackett 2007), organizational deviance (OD) is working slowly, damaging company property, sharing confidential company information (Berry, Ones, and Sackett 2007), privacy invasion (e.g., taking stationery, items without permission (Martin and Hine 2005), physical violence on property (Ménard, Brunet, and Savoie 2011) 3. gossip (Berry, Ones, and Sackett 2007), verbal harassment, sexual harassment (Ménard, Brunet, and Savoie 2011), puts others at disadvantage (Litzky, Eddleston, and Kidder 2006), interpersonal deviance such as making fun of someone at work (Bennett and Robinson 2000) 4. violates significant organizational norms (Robinson and Bennett, 2000), disadvantage on sociodemographic variables (Ménard, Brunet, and Savoie 2011), rule breaking on minority groups, underclass, colored people (Cicourel 1998), violate on gender differences (Chernyak-Hai, Kim, and Tziner 2018) 5. cyber deviance (Weatherbee and Kelloway, 2006) 6. regularly rule breaking (Pfuhl and Henry 1993), primary deviance can be systematically reinforced by societal reactions into secondary deviance (Lemert 1997), 7. ﻿last year, ranging from ﻿never, to once a year, twice a year, several times a year, monthly, weekly, 7 daily (Workplace Deviance Scale, Bennett and Robinson 2000) 8. from minor to serious, on a level of severity (Robinson and Bennett, 1995), develops as a domino-pattern/cycle of deviance (Robinson, 2008), stress leads to higher level of interpersonal deviance (Bashir et al. 2019) 9. ﻿overt and covert conduct problems in conversation and role taking (Snijder et al. 2005) |
| Counterproductive Work A  Behavior (CWB)  A  A  A  A  A  A  A  A | 1. Negative physical acts (Greenberg and Scott 1996), violent and aggressive behaviors (Barling, Dupré, and Kelloway 2009), inappropriate physical attention (Gruys, Stewart, and Bowling 2010), physical sexual advances toward co-workers (Gruys 1999) 2. sabotage (e.g., physically damaging organizational property; Ambrose, Seabright, and Schminke 2002), service sabotage (Harris and Ogbonna 2002), destruction of property, misuse information (Gruys and Sackett 2003), CWBO, ﻿counterproductive work behaviors toward the organization e.g. property theft, production deviance (e.g., intentionally working slowly, doing work incorrectly, or neglecting to follow procedures), withdrawal (e.g., taking longer breaks than allowed, arriving late, leaving early) (Spector et al. 2006), time theft (Brock, Martin, and Buckley 2013), nested as: bad behavior at work including *deviance, politics, injustice, unsafety, misuse, bullying, harassment* (Furnham and Taylor 2011), poor quality of work (Gruys and Sackett 2003), knowledge hiding (Serenko 2019) 3. acting uncivil (Andersson and Pearson 1999), interpersonal gossip (Bennett and Robinson, 2000; Neuman and Baron, 2005), unsafe behavior, drug and alcohol use, poor attendance (Gruys and Sackett 2003), revengeful behavior (Bies and Tripp 2005),﻿ abuse against others, ignoring or arguing with others (Spector et al. 2006), exclusion of workers (Hitlan and Noel 2009), abusive behavior (Spector, Fox, and Domagalski 2006), CWBI, counterproductive work behaviors toward the individual e.g. unethical, resistant, passive obedient behavior, loophole seeking, storytelling, knowledge withholding (Peng 2012) 4. CWBP counterproductive work behavior targeting persons e.g. make fun of someone’s personal life (Robinson and Bennett 1995), gender as moderator (Spector and Zhou 2014), exclusion of social groups in production (Hitlan, Cliffton, and DeSoto, 2006) 5. counterproductive use of technology: surfing during work hours (Lim, 2002; Weatherbee, 2010), identity theft (Neese, Ferrell, and Ferrell 2003), cyber fraud (Trembly 2014), cyber loafing (Lim, 2002) 6. repeatedly (Serenko 2019) 7. ﻿percentage of participants who indicated to have conducted the behavior at least once during the last twelve months (Marcus et al. 2002) 8. escalation due to different reasons (Zapf and Gross, 2001), increasing by seeking social support, confronting the perpetrator or tell their supervisors (Cortina and Magley 2003), ﻿escalates by negative reciprocity norms (Gibney, Zagenczyk, and Masters 2009), spirals by inflated self-esteem (Fisk 2010) 9. ﻿occurs in private and covert (Khan, Quratulain, and Bell 2014), covert behavior (Fox et al. 2007), overt behaviors, and covert damaging behaviors are a strategy to reduce the emotionally unpleasant condition (Spector 1998; Penney and Spector 2005) |
| Violence A  A  A  A  A  A  A  A  A | 1. serious instances of physical assaults (Baron and Neuman, 1998), with physical strength or power (Campo and Klijn 2018), negative physical occupational behavior (Azodo, Ezeja, and Ehikhamenor 2011), physical attack at work (Angland, Dowling, and Casey 2014) 2. taking risks for the security, or health of the worker (Wolf, Delao, and Perhats 2014) 3. any unreasonable action, incident or behavior in which a person is threatened, humiliated by another person while performing his/her professional activities or as a consequence of such actions (Angland, Dowling, and Casey 2014), non-verbal cues of speech (Rominiecka 2008), verbal abuse, and bullying, harassing behavior (Cheung, Lee, and Yip 2017), loud shouting, threat, sexual harassment, swearing (Azodo, Ezeja, and Ehikhamenor 2011) 4. gender and class-related assaults (Rickett and Roman 2012), sexual, racial related assaults (Cheung, Lee, and Yip 2017), assaults on Muslins (Agrawal et al. 2019) 5. technology facilitated violence (Henry and Powell 2016) 6. violence becomes structural when dehumanization of the other is institutionalized (Galtung 1988), increases by repeatedly inappropriate behaviors (Boyd 2002) 7. ﻿twelve months, ranging from not at all, to infrequently (a few times in 12 months), occasionally (a few times each six months), often (a few times each month), frequently (once or more each week) terrorism and threats, on physical and psychological violence such as verbal abuse, bullying/mobbing, harassment (sexual, racial, age, marital status, socioeconomic status), ﻿ILO, ICN, WHO and PSI, report in the health care sector in Brazil, Bulgaria, Lebanon, Portugal, South Africa, Thailand, Australia (Di Martino 2009) 8. escalation in violence, resulting in intense aggressive acts (Martinez et al. 2008) 9. involves covert or lower-level forms of aggression (e.g. bullying) with ﻿subtle variations in perpetrator behaviors, as well as more explicit forms (e.g. assaults) (Mayhew et al. 2004), covert and subtle violence such as withholding information or disseminating gossip and overt and direct such as criticism in front of other staff, false accusations, or menacing body language (Thomas and Burk 2009) |
| Abuse A  /Abusive supervision  A  A  A  A  A  A  A  A | 1. physical abuse (Kisa 2008),﻿ ﻿abusive supervision aligns with supervisor aggression, ﻿including physical hostility (Grandey, Kern, and Frone 2007) 2. material and economical abuse (Poole 2010), abuse by supervisors to control or force organizational outcomes (Einarsen et al., 2003), supervisor undermining behavior intended to hinder work related success, and favorable reputation (Duffy, Ganster, and Pagon 2002) 3. emotional abuse is repeated verbal and nonverbal (but nonphysical), hostile behaviors (Keashly and Harvey 2005), verbal abuse (Kisa 2008), a specific type of harassment (Bambi et al. 2018), in abusive supervision power is used with hostility (Tepper 2000, 2007) 4. procedural unfair with the Universal Declaration of Human Rights of 1948 (Industrial Relations Services 1999), destructive communication by more powerful members toward less powerful members in the workplace (Lutgen-Sandvik 2003), sexual related abuse (Kisa 2008), ageism related abuse (Brownell and Powell 2013) 5. internet abuse (Churchman 2003), hacking, unauthorized entry into co-workers or supervisor's computers (Stafford and Urbaczewski 2004), misuse of information, and problematic use on internet (Caplan 2007) 6. repetitive patterns (Keashly and Harvey 2005), duration of exposure (Keashly and Jagatic 2010) 7. in the past few months, ranging from never to seldom, sometimes, often (abusive supervision15-item instrument, Tepper 2000) 8. escalating (Leafloor and Biggs 1993), spiral (Mawritz et al. 2012; Yu and Duffy 2015) 9. ﻿overt and covert physical and psychological abuse (Shahtahmasebi 2004) |
| Terror A  A  A  A  A  A  A  A  A | 1. physical attacks, stalking, following, loitering (Every-Palmer, Barry-Walsh, and Pathé 2015), frequent changes of tasks to punish (Leymann 1996) ﻿ 2. property interference (Every-Palmer, Barry-Walsh, and Pathé 2015) 3. negative communication, humiliating behavior (Leymann 1996; Matthiesen and Einarsen 2007)﻿, attacking a person’s integrity, isolation, direct and indirect critique, sanction by certain tasks, threats, sexual encroachment (Leymann and Niedl 1994), tyranny leadership: arbitrariness and self-aggrandizement, belittling sub-ordinates, lack of consideration, forcing style of conflict resolution, discouraging initiative, noncontingent punishment (Ashforth 1994) 4. social isolating behavior, violence or threat (Leymann 1996), attacking a person’s private sphere (Leymann and Niedl 1994) ﻿ 5. cyberstalking (Every-Palmer, Barry-Walsh, and Pathé 2015) 6. ﻿repeated attacks (Bayramoğlu and Toksoy 2017), occurring at least once a week over a 6-month period (Leymann 1996) 7. In current work, questions being a victim, ranging from completely disagree, to strongly disagree, fairly disagree, neither disagree nor agree, fairly agree, strongly agree, completely agree (Leymann Inventory of Psychological Terror; LIPT: Leymann 1990) 8. cycle of escalation, organizational aspects reinforces victimization (Leymann 1996), stress and anxiety are both antecedent and consequence, working in a vicious circle (Reijntjes et al. 2010), ﻿high task interdependence and prolonged work in physical proximity contribute to a worsening process (Hershcovis and Barling 2007) 9. covert are illegal, dark persons or networks, and the opposite are legal, public persons or networks for political, social, economic action (Raab 2003) |
| Injustice A  A  A  A  A  A  A  A  A | 1. willingness to punish transgressors in judgement tasks (Goldberg, Lerner, and Tetlock 1999) 2. perceptions of inequity between workers allocation decisions, no procedural justice (Elovainio, Kivimaki, and Vahtera 2002), unfair distribution of outcome allocation, no distributive justice (Tepper 2000) 3. hostile insults and jokes (Alleyne 2004), bullying behavior (Krieger et al. 2006), discrimination of workers (Rospenda, Richman, and Shannon 2009), harassment, abusive behavior (Okechukwu et al. 2014), act of omission/commission (Bennett, 1983), unfairness of the interpersonal treatment individuals receive during the enactment of procedures, no interactional justice ﻿(Skarlicki and Folger 1997) 4. isolating or excluding workers (Alleyne 2004), unfair treatment based on age (Gee, Pavalko, and Long 2007), gender/sex related injustice (Pina, Gannon, and Saunders 2009), injustice on disability (Snyder et al. 2010; Moore et al. 2011), race/color related unequal treatment (Raver and Nishii 2010), unequal decisions based on genetic information, national origin, pregnancy or religion (EEOC 2011) ﻿ 5. injustice build in systems discriminating on gender, race and power in AI (West, Crawford, and Whittaker 2019), cybercrime, blackmail (Henry and Powell 2016) 6. no onetime event, unfolds across time, as do victims’ responses (Fitzgerald, Swan, and Fischer 1995), unfolds within-persons taking several months or longer to become apparent (Neall and Tuckey 2014) 7. ﻿various timeframes (Colquitt and Rodell 2015), procedural and distributive over periods of four weeks (Holtz and Harold 2009, ) to ﻿three months (Hausknecht and Sturman 2011), and interpersonal and information justice over periods of a day, a week, a month (Hausknecht and Sturman 2011), depends on the type of justice, ranging from a very small extent to a small extent, a moderate extent, a large extent, a very large extent (Colquitt 2001) 8. ﻿typical escalating process (Zapf and Gross, 2001) 9. ﻿workers motivated by injustice focus on more ‘‘subtle and covert’’ forms (Robinson and Bennett 1995), overtly hostile actions (Alleyne 2004) |
| Interpersonal conflict A  A  **A**  A  A  A  A  A  A | 1. physical assault (Spector and Jex 1998), voice disagreement in a physical manner (Einarsen et al., 2003) 2. ﻿actions to gain in resources (Hobfoll and Shirom 2000), struggle on realization of material outcomes (Van der Vliert 1997) 3. disagreement in an emotional, personal, or professional manner (Einarsen et al., 2003), many types of interpersonal mistreatment in the workplace, including workplace rudeness (Johnson and Indvik, 2001), verbal aggression (Grandey, Dickter, and Sin 2004), bullying acts (Nielsen, Matthiesen, and Einarsen 2008), interpersonal mistreatment behaviors in the workplace, such as rude behavior, yelling, or other interpersonally deviant acts (Spector and Jex 1998) 4. race-based conflicts between workers (waters 1992), sexual need based (Hoskins 1983), male dominated management with sexual harassment and discrimination (De Dreu, van Dierendonck, and Dijkstra 2004) 5. negative interpersonal behavior/ raising conflicts of millennials through social media (Kavitha and Bhuvaneswari 2016) 6. escalation of aggression by the involved parties and witnesses/ negative hostile actions and reactions (Andersson and Pearson 1999; Mitchell and Ambrose 2007), blow up the conflict broader and last longer (Van de Vliert 2010) 7. ﻿Current work, ranging from once per month or never, to once or twice per month, once or twice per week, once or twice per day, several times per day (Interpersonal Conflict at Work Scale, ﻿ICAWS: Spector and Jex 1998) 8. spiral of conflict, denying and pushing each other to more drastic measures of both parties (Zapf and Gross 2001), into higher emotional levels (Leon-Perez et al. 2015), escalating into harsh interpersonal conflict (Zahlquist et al. 2019) 9. can manifest itself in both overt and subtle forms (Spector and Jex 1998), covert punish messages ( De Dreu, van Dierendonck, and Dijkstra 2004) |
| Victimization A  /Scapegoating  A  A  A  A  A  A  A  A | 1. physical victimization by aggressive, externalizing behavior with a bodily component (Chan 2006), ﻿cost and reform pressures to intensify job insecurity, resulting in scapegoating and blame for worker compliance (Cooke 2012) 2. portrayal in media (Reichert and Carpenter 2004), sex in advertising as pin-up (Rosewarne 2007), assault, burglary, robbery, theft, vandalism, and sexual assault (Engel et al. 2015) 3. psychological acts of aggression, the exclusion of one on personal characteristics (Cowan 2012) 4. making someone a victim because of masculinity, discourse, ideology, and power (Scarduzio and Geist-Martin 2010), being victimized because of gender, minority race/ethnicity, same-sex attraction, alcohol binge drinking (Clear et al. 2014),﻿ disadvantaged groups, are victimized because they are scorned by both society and the justice (Athwal and Burnett 2014), victimization on vulnerability traits (Bowling and Beehr 2006; Milam, Spitzmueller, and Penney 2009) 5. cyberstalking victimization (Reyns, Henson, and Fisher 2012) 6. over time performed weekly (Trépanier, Fernet, and Austin 2013), chronically victimized (Chan 2006) 7. before this school / in this school bullied: how long did the attacks last: I wasn’t bullied, just a few days, weeks, months, a year or more, last seven days: 0, 1, 2, 3, 4, 5, 6+ times (Bully-Victim scale tools: Hamburger, Basile, and Vivolo 2011); ﻿34 different types of victimization from the time they were born until 17 years of age, ranging from no times, to 1 time, 2 times, 3 times, 4 times, or 5 or more times (﻿JVQ–Adult Retrospective Version) 8. persistent escalation (Cowan 2012), mechanism that can escalate (Hutchinson 2013), aggressor increases, covert rather than overt actions also increases (Baron and Neuman, 1998), serial bullies subject victims to more serious forms of aggression (Chan 2006) 9. ﻿covert forms of victimization ﻿taking into consideration the risks (Jensen, Patel, and Raver 2014), ﻿openly or covertly express frustration influenced by self-esteem issues and past exposure, covert form with disguised, subtle actions: swearing or yelling, withholding job-related information, give the “silent treatment” (Kaukiainen et al. 2001) |
| Micropolitics A  A  A  A  A  A  A  A  A | 1. body language of eyes, hands, speech, legs, posture (Rominiecka 2008) 2. competing for the same resources, work environment is characterized as self-serving of various individuals and groups, at the cost of other individuals or groups (Ferris, Harrell-Cook, and Dulebohn, 2000), enhance one’s personal and/or organizational objectives (Ferris et al. 2007), fake news (Farkas and Schou 2018) 3. harassment to survive in organizational contexts (Bühler and Zapf 1997), pejorative or unfair treatment (Thomas and Burk 2009), undervalue workers (Pearcey and Elliott 2004), verbal abusive ats (Ferns and Meerabeau 2009), domination and oppression (Canales 2010), ignore or unwelcome persons (Hoel, Giga, and Davidson 2007), 4. treat people differently on gender and minority groups (Maccallum 2002; Slavin, Batrouney, and Murphy 2007; Bach 2005), ﻿organizationally and socially harmful activities and relationships (Ferris, Harrell-Cook, and Dulebohn 2000), handle unequally on demographic variables such as female, age, race, minority, longer employment tenure (Adams, Treadway, and Stepina 2008; Treadway, Adams, and Goodman 2005) 5. internet politics (Fung, Russon Gilman, and Shkabatur 2013) 6. system of representation of power (Foucault 1980), organizational interests are fabricated and reproduced as a process of libidinal production (Webb 2008) 7. current work, perceptions of organizational politics: e.g. anxiety produced by politics, ranging from not at all, to somewhat, moderately, very much so (Zhou and Ferris 1995), context of current work environment, ranging from strongly disagree, to disagree, neutral agree, strongly agree (Political skill Inventory: PSI, Ferris et al. 2005) 8. escalation (Canales 2010) 9. visible and not visible (Vallant and Neville 2006), covertly power, not easily observed, not observed at all (Webb 2008) |
| Ostracism A  A  A  A  A  A  A  A    A | 1. exclusion of rites and rituals that signify organizational membership (Anderson, 2009) 2. only respected, not ostracized, will be given control over coveted resources (Magee and Galinsky 2008) 3. (de)-identification process to exclude or not (Riketta 2005), spread of reputational information through gossip (Feinberg, Willer, and Schultz 2014), treat someone differently based on demographic variables such as female, age, race, minority, longer employment tenure (Adams, Treadway, and Stepina 2008), exclude someone from meaningful existence depends on low self-esteem (Gonsalkorale and Williams 2007) 4. social procedure under the Athenian democracy: any citizen could be expelled from the city-state of Athens for ten years (Livius), deliberately left out of a group or social setting by exclusion and rejection (Williams and Sommer 1997), excluding people along gender, age, and demographic lines (Williams 1997), the excluding behavior reflects the guidelines of the social environment in which employees work (Ehrhart 2004) 5. internet ostracism (Zadro, Williams, and Richardson 2004) 6. Shunning: not brief and infrequent but systematic exclusion of an individual who was once an included member of the group (Anderson, 2009), revolves with continued reinforcement (Schein 2010) 7. being ostracized, ranging from never, to once in a while, sometimes, fairly often, often, constantly, always (Workplace ostracism scale: WOS, Ferris et al. 2008) 8. inappropriate﻿ behavioral strategies make the situation worse (Williams and Zadro 2005), processes of social influence (Jones 1990), ingratiation to perform properly (Treadway et al. 2007) 9. ﻿extreme and complete forms of ostracism are exile and banishment, less dramatic are using the silent treatment or avoiding eye contact (Ferris et al. 2008) |
| Incivility A  A  A  A  A  A  A  A  A | 1. ﻿hostile looks (Cortina et al. 2013), nonverbal disrespectful behaviour such as glaring at someone (Lim, Cortina, and Magley, 2008) 2. give no information needed by the target (Andersson and Pearson, 1999) 3. low-intensity deviant behaviour (Andersson and Pearson 1999), insulting the target (Glomb 2002), disrespectful remarks, or refusal to collegial work (Cortina et al. 2013), ﻿impolite manner, talking negatively about another (Blau and Andersson 2005; Martin and Hine 2005) 4. ignoring, or excluding colleagues (Lim, Cortina, and Magley, 2008), norm violation (Pearson, Andersson, and Wegner, 2001), race targetted uncivil acts (Kern and Grandey 2009), uncivil acts on gender (Gallus et al. 2014), modern sexism, racism (Cortina et al. 2013), ignoring, rude remarks (Porath and Pearson, 2013), deviates from workplace norms of mutual respect (Pearson et al., 2000), bystanders acting during uncivil social encounters (Pearson et al., 2000)﻿, from a social interactionist perspective, the social environment influences rude incivil behavior (Andersson and Pearson 1999) 5. computer flaming (Pearson et al., 2001), cyber-incivility (Park, Fritz, and Jex, 2018), active and passive e-mail incivility (Yuan, Park, and Sliter 2020), rude e-mail (Blau and Andersson 2005; Martin and Hine 2005) 6. chronic, not a single, static event but an interactive process (Pearson, Andersson, and Porath, 2005), reciprocal process over time (Schilpzand, De Pater, and Erez 2016) 7. ﻿duration of 1 year, ranging ﻿from never, to once or twice, sometimes, often, and many times (Uncivil ﻿Workplace Behavior Questionnaire: UWBQ, Martin and Hine 2005); during the preceding 5 years, from never to rarely, occasionally, often, very often (Workplace Incivility Scale: WIS, Cortina et al. 2001); non-measurement literature indicates also: past year (Walsh et al. 2012; Ferguson 2012), repeating in 2 weeks to a month (Kern and Grandey, 2009; Leiter, Laschinger, Day, and Oore, 2011; Sakurai and Jex, 2012; Sliter, Sliter, Withrow, and Jex, 2012; van Jaarsveld, Walker, and Skarlicki, 2010), or epeating in past 6, 4 or 3 months (Wilson and Holmvall 2013; Blau 2007; Scott, Restubog, and Zagenczyk 2013) 8. incivility spiral, ﻿uncivil acts can potentially spawn a spiral of increasingly aggressive events (Andersson and Pearson 1999), low-level incivilities can escalate in extreme and intense (Lutgen-Sandvik 2003) 9. lesser powerful targets with fear are more likely to choose covert indirect responses, as opposed to more direct aggression against the instigators (Porath and Pearson 2012), overt includes techniques like extreme micromanaging, verbal criticism, name-calling, insults, and direct threats. Covert incivility is indirect and passive-aggressive, and can include rumors and gossip, withholding information, unfair assignments, low grades or undesirable tasks as punishment, and sabotage. Microaggressions—brief and common- place indignities denigrating a person’s race, religion, or membership in another marginalized group—are also considered as a form of covert behaviors (Edmonson and Zelonka 2019) |
| Social safety A  A  A  A  A  A  A  A  A | 1. physical assault (Macdonald and Sirotich 2005), hitting, violent attacks, kicking, grabbing, scratching (Cele 2018; Bernaldo-De-Quirós et al. 2015), possession/use of weapons, violence related with alcohol, drugs, LGBT use (Nelen et al. 2018) 2. vandalism and theft (Steinberg, Allensworth, and Johnson 2011; Schuilenburg, Coenraads, and Van Calster 2009) 3. shouting, verbal harassment, ﻿threat with physical harm (Macdonald and Sirotich 2005), ﻿yelling, swearing, cursing (Bernaldo-De-Quirós et al. 2015), psychological harassment, verbal violence (Cavalcanti et al. 2018), interchangeable with bullying, negative interpersonal interactions, mobbing, harassment emotional/psychological abuse (Park, Lee, and Park, 2017) 4. harassment, sexual abuse, sexual norm violation and violence (Nelen et al. 2018), active distrust of foreigners and government, vulnerability in lack of employment, poor housing and education leading to anti-social behavior on woman, children and environment (Ransley and Mazerolle 2009) 5. in social media: e.g. Cyber violence on Twitter (Nagle 2018), cyber-enabled, and -dependent (Furnell 2002) 6. regularly, day-to-day incidents (Steinberg, Allensworth, and Johnson 2011), physical aggression often combines with frequently verbal aggression (Bernaldo-De-Quirós et al. 2015), repeatedly, regularly (Park et al., 2017) 7. ﻿twelve months, ranging in Never/ Sometimes/ Usually/ Always (﻿risk stratification tool for safety survey; Ruiz-Hernández et al. 2016)   last year, ranging from never/seldom, sometimes, often, very often (Social Safety Index: SVI, Verschuren 2009b, 2012, 2009a)   1. escalation of behavior leading to assaults / de-escalation to prevent these (Health and safety department US, 2016), ﻿escalating conflicts and reinforced negative patterns (Steinberg, Allensworth, and Johnson 2011) 2. visibility of physical and material acts (Cele 2018); covert; possesion of weapons (Nelen et al. 2018), overt or covert behavior (Eagly and Chaiken 1993) |

# References Table 2 |

Adams, G. L., D. C. Treadway, and L. R. Stepina. 2008. “The Role of Dispositions in Politics Perception Formation: The Predictive Capacity of Negative and Positive Affectivity, Equity Sensitivity, and Self- Efficacy.” *Journal of Managerial Issues* 20 (4): 545–63. https://www.jstor.org/stable/40604628.

Agrawal, Priyanka, Yousra Yusuf, Omrana Pasha, Shahmir H. Ali, Homayra Ziad, and Adnan A. Hyder. 2019. “Interpersonal Stranger Violence and American Muslims: An Exploratory Study of Lived Experiences and Coping Strategies.” *Global Bioethics* 30 (1): 28–42. https://doi.org/10.1080/11287462.2019.1683934.

Alleyne, Aileen. 2004. “Black Identity and Workplace Oppression.” *Counselling and Psychotherapy Research* 4 (1): 4–8. https://doi.org/10.1080/14733140412331384008.

Ambrose, Maureen L, Mark A Seabright, and Marshall Schminke. 2002. “Sabotage in the Workplace: The Role of Organizational Injustice.” *Organizational Behavior and Human Decision Processes* 89 (1): 947–65. https://doi.org/10.1016/S0749-5978(02)00037-7.

Anderson, Craig A., and Brad J. Bushman. 2002. “Human Aggression.” *Annual Review of Psychology* 53 (1): 27–51. https://doi.org/10.1146/annurev.psych.53.100901.135231.

Anderson, Janice W. 2009. “Organizational Shunning: The Disciplinary Functions of ‘Non-Sense.’” *Atlantic Journal of Communication* 17 (1): 36–50. https://doi.org/10.1080/15456870802506140.

Andersson, Lynne M, and Christine M Pearson. 1999. “Tit for Tat? The Spiraling Effect of Incivility in the Workplace.” *The Academy of Management Review* 24 (3): 452–71. https://doi.org/10.2307/259136.

Angland, Shirley, Maura Dowling, and Dympna Casey. 2014. “Nurses’ Perceptions of the Factors Which Cause Violence and Aggression in the Emergency Department: A Qualitative Study.” *International Emergency Nursing* 22 (3): 134–39. https://doi.org/10.1016/j.ienj.2013.09.005.

Ashforth, Blake. 1994. “Petty Tyranny in Organizations.” *Human Relations* 47 (7): 755–78. https://doi.org/10.1177/001872679404700701.

Athwal, Harmit, and Jon Burnett. 2014. “Investigated or Ignored? An Analysis of Race-Related Deaths since the Macpherson Report.” *Race & Class* 56 (1): 22–42. https://doi.org/10.1177/0306396814531694.

Azodo, C. C., E. B. Ezeja, and E. E. Ehikhamenor. 2011. “Occupational Violence against Dental Professionals in Southern Nigeria.” *African Health Sciences* 11 (3): 486–92. https://www.ajol.info/index.php/ahs/article/view/73420.

Bach, Betsy Wackernagel. 2005. “The Organizational Tension of Othering.” *Journal of Applied Communication Research* 33 (3): 258–68. https://doi.org/10.1080/00909880500149478.

Bambi, Stefano, Chiara Foà, Christian De Felippis, Alberto Lucchini, Andrea Guazzini, and Laura Rasero. 2018. “Workplace Incivility, Lateral Violence and Bullying among Nurses. A Review about Their Prevalence and Related Factors.” *Acta Biomedica* 89 (6): 51–79. https://doi.org/10.23750/abm.v89i6-S.7461.

Baran Tatar, Zeynep, and Sahika Yuksel. 2018. “Mobbing at Workplace - Psychological Trauma and Documentation of Psychiatric Symptoms.” *Archives of Neuropsychiatry* 56 (1): 57–62. https://doi.org/10.29399/npa.22924.

Barling, Julian, Kathryne E. Dupré, and E. Kevin Kelloway. 2009. “Predicting Workplace Aggression and Violence.” *Annual Review of Psychology* 60 (1): 671–92. https://doi.org/10.1146/annurev.psych.60.110707.163629.

Baron, R. A., and J. H. Neuman. 1998. “Workplace Violence and Workplace Aggression: Evidence Concerning Specific Forms, Potential Causes, and Preferred Targets.” *Journal of Management* 24 (3): 391–419. https://doi.org/10.1177/014920639802400305.

Baruch, Yehuda. 2005. “Bullying on the Net: Adverse Behavior on e-Mail and Its Impact.” *Information and Management* 42 (2): 361–71. https://doi.org/10.1016/j.im.2004.02.001.

Bashir, Mohsin, Muhammad Abrar, Muhammad Yousaf, Shahnawaz Saqib, and Rizwan Shabbir. 2019. “Organizational Politics and Workplace Deviance In Unionized Settings: Mediating Role of Job Stress and Moderating Role of Resilience.” *Psychology Research and Behavior Management* Volume 12 (October): 943–59. https://doi.org/10.2147/PRBM.S213672.

Bayramoğlu, Mahmut M., and Devlet Toksoy. 2017. “Leadership and Bullying in the Forestry Organization of Turkey.” *BioMed Research International* 2017: 1–11. https://doi.org/10.1155/2017/9454682.

Bennett, Jonathan. 1983. “Positive and Negative.” *American Philosophical Quarterly 20* 20 (1): 185–94. https://doi.org/10.1016/S0140-6736(00)97680-3.

Bennett, Rebecca J., and Sandra L. Robinson. 2000. “Development of a Measure of Workplace Deviance.” *Journal of Applied Psychology* 85 (3): 349–60. https://doi.org/10.1037/0021-9010.85.3.349.

Bernaldo-De-Quirós, Mónica, Ana T. Piccini, M. Mar Gómez, and Jose C. Cerdeira. 2015. “Psychological Consequences of Aggression in Pre-Hospital Emergency Care: Cross Sectional Survey.” *International Journal of Nursing Studies* 52 (1): 260–70. https://doi.org/10.1016/j.ijnurstu.2014.05.011.

Berry, Christopher M., Deniz S. Ones, and Paul R. Sackett. 2007. “Interpersonal Deviance, Organizational Deviance, and Their Common Correlates: A Review and Meta-Analysis.” *Journal of Applied Psychology* 92 (2): 410–24. https://doi.org/10.1037/0021-9010.92.2.410.

Bies, Robert J., and Thomas M. Tripp. 2005. “The Study of Revenge in the Workplace: Conceptual, Ideological, and Empirical Issues.” In *Counterproductive Work Behavior: Investigations of Actors and Targets.*, edited by Suzy Fox and Paul E. Spector, 65–81. Washington: American Psychological Association. https://doi.org/10.1037/10893-003.

Björkqvist, Kaj, Karin Österman, and Kirsti M. J. Lagerspetz. 1994. “Sex Differences in Covert Aggression among Adults.” *Aggressive Behavior* 20 (1): 27–33. https://doi.org/10.1002/1098-2337(1994)20:1<27::AID-AB2480200105>3.0.CO;2-Q.

Blau, Gary. 2007. “Partially Testing a Process Model for Understanding Victim Responses to an Anticipated Worksite Closure.” *Journal of Vocational Behavior* 71 (3): 401–28. https://doi.org/10.1016/j.jvb.2007.08.005.

Blau, Gary, and Lynne Andersson. 2005. “Testing a Measure of Instigated Workplace Incivility.” *Journal of Occupational and Organizational Psychology* 78 (4): 595–614. https://doi.org/10.1348/096317905X26822.

Bowling, Nathan A., and Terry A. Beehr. 2006. “Workplace Harassment from the Victim’s Perspective: A Theoretical Model and Meta-Analysis.” *Journal of Applied Psychology* 91 (5): 998–1012. https://doi.org/10.1037/0021-9010.91.5.998.

Boyd, Carol. 2002. “Customer Violence and Employee Health and Safety.” *Work, Employment and Society* 16 (1): 151–69. https://doi.org/10.1177/09500170222119290.

Brock, Meagan E., Laura E. Martin, and M. Ronald Buckley. 2013. “Time Theft in Organizations: The Development of the Time Banditry Questionnaire.” *International Journal of Selection and Assessment* 21 (3): 309–21. https://doi.org/10.1111/ijsa.12040.

Brownell, Patricia, and Mebane Powell. 2013. “Definitions and Theoretical Models for Understanding Ageism and Abuse in the Workplace.” In *Ageism and Mistreatment of Older Workers*, 17–28. Dordrecht: Springer Netherlands. https://doi.org/10.1007/978-94-007-5521-5_2.

Bühler, K, and Dieter Zapf. 1997. *Stigmatisierung Am Arbeitsplatz. Eine Qualitative Studie Mit Mobbingopfern (Stigmatisation at Work. A Qualitative Study with Bullying Victims)*. University of Konstanz: Social Science Faculty.

Buss, Arnold H., and Mark Perry. 1992. “The Aggression Questionnaire.” *Journal of Personality and Social Psychology* 63 (3): 452–59. https://doi.org/10.1037/0022-3514.63.3.452.

Campo, Varinia Rodríguez, and Tatiana Paravic Klijn. 2018. “Verbal Abuse and Mobbing in Pre-Hospital Care Services in Chile.” *Revista Latino-Americana de Enfermagem* 25 (January): e2956. https://doi.org/10.1590/1518-8345.2073.2956.

Canales, Mary K. 2010. “Othering: Difference Understood?” *Advances in Nursing Science* 33 (1): 15–34. https://doi.org/10.1097/ANS.0b013e3181c9e119.

Caplan, Scott E. 2007. “Relations among Loneliness, Social Anxiety, and Problematic Internet Use.” *CyberPsychology & Behavior* 10 (2): 234–42. https://doi.org/10.1089/cpb.2006.9963.

Cavalcanti, Alessandro Leite, Eduardo dos Reis Belo, Emanuella de Castro Marcolino, Américo Fernandes, Yuri Wanderley Cavalcanti, Danielle Franklin de CARVALHO, Ana Maria Gondim Valença, Alidianne Fabia Cabral Cavalcanti, and Wilton Wilney Nascimento Padilha. 2018. “Occupational Violence against Brazilian Nurses.” *Iranian Journal of Public Health* 47 (11): 1636–43. http://ijph.tums.ac.ir.

Cele, B. 2018. “Addendum to the Saps Annual Report.” South African Police Service.

Champion, David R. 2006. “Sexual Harassment: Criminal Justice and Academia.” *Criminal Justice Studies* 19 (2): 101–9. https://doi.org/10.1080/14786010600764443.

Chan, John H.F. 2006. “Systemic Patterns in Bullying and Victimization.” *School Psychology International* 27 (3): 352–69. https://doi.org/10.1177/0143034306067289.

Chernyak-Hai, Lily, Se-Kang Kim, and Aharon Tziner. 2018. “Gender Profiles of Workplace Individual and Organizational Deviance.” *Journal of Work and Organizational Psychology* 34 (1): 46–55. https://doi.org/10.5093/jwop2018a6.

Cheung, Teris, Paul Lee, and Paul Yip. 2017. “Workplace Violence toward Physicians and Nurses: Prevalence and Correlates in Macau.” *International Journal of Environmental Research and Public Health* 14 (8): 879. https://doi.org/10.3390/ijerph14080879.

Churchman, P. 2003. “Abusing the Net - How to Curb Work Surfers.” *New Zealand Management* 50 (8): 46–47. https://www.questia.com/magazine/1G1-111064659/abusing-the-net-how-to-curb-work-surfers-with-surfing.

Cicourel, Aaron V. 1998. “Review on Edwin Lemerts’s Book ‘The Trouble with Evil:Social Control at the Edge of Morality.’” *Contemporary Sociology* 27 (2): 201–2. http://www.jstor.org/stable/2654819.

Clear, Emily R., Ann L. Coker, Patricia G. Cook-Craig, Heather M. Bush, Lisandra S. Garcia, Corrine M. Williams, Alysha M. Lewis, and Bonnie S. Fisher. 2014. “Sexual Harassment Victimization and Perpetration among High School Students.” *Violence Against Women* 20 (10): 1203–19. https://doi.org/10.1177/1077801214551287.

Colquitt, Jason A. 2001. “On the Dimensionality of Organizational Justice: A Construct Validation of a Measure.” *Journal of Applied Psychology* 86 (3): 386–400. https://doi.org/10.1037/0021-9010.86.3.386.

Colquitt, Jason A., and Jessica B Rodell. 2015. “Measuring Justice and Fairness.” In *The Oxford Handbook of Justice in the Workplace*, edited by Russell S. Cropanzano and Maureen L. Ambrose, 1–30. London: Oxford University Press. https://doi.org/10.1093/oxfordhb/9780199981410.013.8.

Conway, Paul Maurice, Thomas Clausen, Åse Marie Hansen, and Annie Hogh. 2016. “Workplace Bullying and Sickness Presenteeism: Cross-Sectional and Prospective Associations in a 2-Year Follow-up Study.” *International Archives of Occupational and Environmental Health* 89 (1): 103–14. https://doi.org/10.1007/s00420-015-1055-9.

Cooke, H. 2012. “Changing Discourses of Blame in Nursing and Healthcare.” In *Thinking Violence in Health Care Set- Tings: A Critical Approach*, edited by D Holmes, A Perron, and T Rudge, 47–66. Surrey ,UK: Ashgate.

Cortina, Lilia M., Dana Kabat-Farr, Emily A. Leskinen, Marisela Huerta, and Vicki J. Magley. 2013. “Selective Incivility as Modern Discrimination in Organizations.” *Journal of Management* 39 (6): 1579–1605. https://doi.org/10.1177/0149206311418835.

Cortina, Lilia M., and Vicki J. Magley. 2003. “Raising Voice, Risking Retaliation: Events Following Interpersonal Mistreatment in the Workplace.” *Journal of Occupational Health Psychology* 8 (4): 247–65. https://doi.org/10.1037/1076-8998.8.4.247.

Cortina, Lilia M., Vicki J. Magley, Jill Hunter Williams, and Regina Day Langhout. 2001. “Incivility in the Workplace: Incidence and Impact.” *Journal of Occupational Health Psychology* 6 (1): 64–80. https://doi.org/10.1037/1076-8998.6.1.64.

Cowan, Renee L. 2012. “It’s Complicated.” *Management Communication Quarterly* 26 (3): 377–403. https://doi.org/10.1177/0893318912439474.

Crawford, Neil. 1999. “Conundrums and Confusion in Organisations: The Etymology of the Word ‘Bully.’” *International Journal of Manpower* 20: 86. https://doi.org/https://doi.org/10.1108/01437729910268678.

Daly, Alison, Renee N. Carey, Ellie Darcey, HuiJun Chih, Anthony D. LaMontagne, Allison Milner, and Alison Reid. 2018. “Workplace Psychosocial Stressors Experienced by Migrant Workers in Australia: A Cross-Sectional Study.” Edited by Sonia Dias. *PLOS ONE* 13 (9): e0203998. https://doi.org/10.1371/journal.pone.0203998.

Dreu, Carsten KW De, Dirk van Dierendonck, and Maria T M Dijkstra. 2004. “Conflict at Work and Individual Well-Being.” *International Journal of Conflict Management* 15 (1): 6–26. https://doi.org/10.1108/eb022905.

Duffy, Michelle K., Daniel C. Ganster, and Milan Pagon. 2002. “SOCIAL UNDERMINING IN THE WORKPLACE.” *Academy of Management Journal* 45 (2): 331–51. https://doi.org/10.2307/3069350.

Eagly, A. H., and S. Chaiken. 1993. *The Psychology of Attitudes.* Harcourt Brace Jovanovich College Publishers.

Edmonson, Cole, and Caroline Zelonka. 2019. “Our Own Worst Enemies.” *Nursing Administration Quarterly* 43 (3): 274–79. https://doi.org/10.1097/NAQ.0000000000000353.

EEOC. 2011. “Laws, Regulations and Guidance MOUs.”

Ehrhart, Mark G. 2004. “Leadership and Procedural Justice Climate as Antecedents of Unit-Level Organizational Citizenship Behavior.” *Personnel Psychology* 57 (1): 61–94. https://doi.org/10.1111/j.1744-6570.2004.tb02484.x.

Einarsen, S. 2000. “Harassment and Bullying at Work.” *Aggression and Violent Behavior* 5 (4): 379–401. https://doi.org/10.1016/S1359-1789(98)00043-3.

Einarsen, S, Helge Hoel, and Guy Notelaers. 2009. “Measuring Exposure to Bullying and Harassment at Work: Validity, Factor Structure and Psychometric Properties of the Negative Acts Questionnaire-Revised.” *Work & Stress* 23 (1): 24–44. https://doi.org/10.1080/02678370902815673.

Einarsen, S, Helge Hoel, Dieter & Zapf, and Cary Lynn Cooper. 2003. *Bullying and Emotional Abuse in the Workplace : International Perspectives in Research and Practice*. Edited by Ståle Einarsen, Helge Hoel, Dieter Zapf, and Cary L. Cooper. 1st ed. London ; New York: Taylor & Francis. https://books.google.es/books.

Einarsen, Ståle, and Bj⊘rn Inge Raknes. 1997. “Harassment in the Workplace and the Victimization of Men.” *Violence and Victims* 12 (3): 247–63.

Elovainio, Marko, Mika Kivimaki, and Jussi Vahtera. 2002. “Organizational Justice: Evidence of a New Psychosocial Predictor of Health.” *American Journal of Public Health* 92: 105–8. https://ajph.aphapublications.org/doi/pdfplus/10.2105/AJPH.92.1.105.

Engel, Robin S., Gabrielle Isaza, Murat Yildirim, and Murat Ozer. 2015. “Enhancing Public Safety: University of Cincinnati Student, Faculty, and Ataff Survey Report.” Cincinnati. https://www.uc.edu/content/dam/refresh/publicsafety-62/docs/reform/fall-2014-enhancing-public-safety-survey.pdf.

Every-Palmer, Susanna, Justin Barry-Walsh, and Michele Pathé. 2015. “Harassment, Stalking, Threats and Attacks Targeting New Zealand Politicians: A Mental Health Issue.” *Australian & New Zealand Journal of Psychiatry* 49 (7): 634–41. https://doi.org/10.1177/0004867415583700.

Farkas, Johan, and Jannick Schou. 2018. “Fake News as a Floating Signifier: Hegemony, Antagonism and the Politics of Falsehood.” *Javnost - The Public* 25 (3): 298–314. https://doi.org/10.1080/13183222.2018.1463047.

Fawzi, Nayla. 2009. *Cyber-Mobbing: Ursachen Und Auswirkungen von Mobbing Im Internet (Cyber-Mobbing: Causes and Effects of Mobbing on the Internet)*. Edited by Klaus Beck, Joachim Höflich, Klaus Kamps, Friedrich Krotz, Wolfgang Schweiger, Werner Wirth, and Band. *Nomos Internet Research*. Band 37. Baden-Baden: Edition Reinhard Fischer.

Feagin, Joe R, and Karyn D McKinney. 2005. *The Many Costs of Racism*. Rowman & Littlefield Publishers. https://books.google.nl/books?id=TMtFIwXUqzAC&lr=&source=gbs_navlinks_s.

Feinberg, Matthew, Robb Willer, and Michael Schultz. 2014. “Gossip and Ostracism Promote Cooperation in Groups.” *Psychological Science* 25 (3): 656–64. https://doi.org/10.1177/0956797613510184.

Ferguson, Merideth. 2012. “You Cannot Leave It at the Office: Spillover and Crossover of Coworker Incivility.” *Journal of Organizational Behavior* 33 (4): 571–88. https://doi.org/10.1002/job.774.

Ferns, Terry, and Elizabeth Meerabeau. 2009. “Reporting Behaviours of Nursing Students Who Have Experienced Verbal Abuse.” *Journal of Advanced Nursing* 65 (12): 2678–88. https://doi.org/10.1111/j.1365-2648.2009.05114.x.

Ferris, D. Lance, Douglas J. Brown, Joseph W. Berry, and Huiwen Lian. 2008. “The Development and Validation of the Workplace Ostracism Scale.” *Journal of Applied Psychology* 93 (6): 1348–66. https://doi.org/10.1037/a0012743.

Ferris, Gerald R., Darren C. Treadway, Robert W. Kolodinsky, Wayne A. Hochwarter, Charles J. Kacmar, Ceasar Douglas, and Dwight D. Frink. 2005. “Development and Validation of the Political Skill Inventory.” *Journal of Management* 31 (1): 126–52. https://doi.org/10.1177/0149206304271386.

Ferris, Gerald R., Darren C. Treadway, Pamela L. Perrewé, Robyn L. Brouer, Ceasar Douglas, and Sean Lux. 2007. “Political Skill in Organizations.” *Journal of Management* 33 (3): 290–320. https://doi.org/10.1177/0149206307300813.

Ferris, Gerald R, Gloria Harrell-Cook, and James H Dulebohn. 2000. “Organizational Politics: The Nature of the Relationship between Politics Perceptions and Political Behavior.” *Research in the Sociology of Organizations* 17: 89–130. https://doi.org/10.1016/S0733-558X(00)17004-1.

Fisk, Glenda M. 2010. “‘I Want It All and I Want It Now!’ An Examination of the Etiology, Expression, and Escalation of Excessive Employee Entitlement.” *Human Resource Management Review* 20 (2): 102–14. https://doi.org/10.1016/j.hrmr.2009.11.001.

Fitzgerald, Louise F., Suzanne Swan, and Karla Fischer. 1995. “Why Didn’t She Just Report Him? The Psychological and Legal Implications of Women’s Responses to Sexual Harassment.” *Journal of Social Issues* 51 (1): 117–38. https://doi.org/10.1111/j.1540-4560.1995.tb01312.x.

Foucault, M. 1980. *Power/Knowledge: Selected Interviews and Other Writings.* New York: Pantheon.

Fox, Suzy, Paul E. Spector, Angeline Goh, and Kari Bruursema. 2007. “Does Your Coworker Know What You’re Doing? Convergence of Self- and Peer-Reports of Counterproductive Work Behavior.” *International Journal of Stress Management* 14 (1): 41–60. https://doi.org/10.1037/1072-5245.14.1.41.

Friedenberg, Joan. 2008. *The Anatomy of an Academic Mobbing.* Lewiston. NY: Edwin Mellen Press.

Fung, Archon, Hollie Russon Gilman, and Jennifer Shkabatur. 2013. “Six Models for the Internet + Politics.” *International Studies Review* 15 (1): 30–47. https://doi.org/10.1111/misr.12028.

Furnell, Steven. 2002. *Cybercrime: Vandalizing the Information Society*. London: Addison-Wesley.

Furnham, A., and J. Taylor. 2011. *Bad Apples: Identify, Prevent & Manage Negative Behavior at Work*. 1st ed. London: Palgrave Macmillan UK.

Gallus, Jessica A., Jennifer A. Bunk, Russell A. Matthews, Janet L. Barnes-Farrell, and Vicki J. Magley. 2014. “An Eye for an Eye? Exploring the Relationship between Workplace Incivility Experiences and Perpetration.” *Journal of Occupational Health Psychology* 19 (2): 143–54. https://doi.org/10.1037/a0035931.

Galtung, Johan. 1988. “Principles of Non Violent Action.” New Yersey. file:///Users/cokkie/Desktop/Johan Galtung - Galtung-Institut.htm.

Gee, G. C., E. K. Pavalko, and J. S. Long. 2007. “Age, Cohort and Perceived Age Discrimination: Using the Life Course to Assess Self-Reported Age Discrimination.” *Social Forces* 86 (1): 265–90. https://doi.org/10.1353/sof.2007.0098.

Gibney, Ray, Thomas J. Zagenczyk, and Marick F. Masters. 2009. “The Negative Aspects of Social Exchange: An Introduction to Perceived Organizational Obstruction.” *Group & Organization Management* 34 (6): 665–97. https://doi.org/10.1177/1059601109350987.

Glomb, Theresa M. 2002. “Workplace Anger and Aggression: Informing Conceptual Models with Data from Specific Encounters.” *Journal of Occupational Health Psychology* 7 (1): 20–36. https://doi.org/10.1037/1076-8998.7.1.20.

Goldberg, Julie H, Jennifer S Lerner, and Philip E Tetlock. 1999. “Rage and Reason: The Psychology of the Intuitive Prosecutor.” *European Journal of Social Psychology* 29 (5–6): 781–95. https://doi.org/10.1002/(SICI)1099-0992(199908/09)29:5/6<781::AID-EJSP960>3.0.CO;2-3.

Gonsalkorale, Karen, and Kipling D. Williams. 2007. “The KKK Won’t Let Me Play: Ostracism Even by a Despised Outgroup Hurts.” *European Journal of Social Psychology* 37 (6): 1176–86. https://doi.org/10.1002/ejsp.392.

González Trijueque, David, and José Luis Graña Gómez. 2010. “Workplace Bullying: Prevalence and Descriptive Analysis in a Multi-Occupational Sample.” *Psicothema* 21 (2): 288–93. http://www.psychologyinspain.com/content/full/2010/14003.pdf.

Grandey, Alicia A., David N. Dickter, and Hock-Peng Sin. 2004. “The Customer Isnot Always Right: Customer Aggression and Emotion Regulation of Service Employees.” *Journal of Organizational Behavior* 25 (3): 397–418. https://doi.org/10.1002/job.252.

Grandey, Alicia A., Julie H. Kern, and Michael R. Frone. 2007. “Verbal Abuse from Outsiders versus Insiders: Comparing Frequency, Impact on Emotional Exhaustion, and the Role of Emotional Labor.” *Journal of Occupational Health Psychology* 12 (1): 63–79. https://doi.org/10.1037/1076-8998.12.1.63.

Greenberg, J, and K.S. Scott. 1996. “Why Do Workers Bite the Hands That Feed Them? Employee Theft as a Social Exchange Process.” In *Research in Organizational Behavior: An Annual Series of Analytical Essays and Critical Reviews.*, edited by B.M. Staw and L.L Cummings, 18:111–56. Science/JAI Press.

Gruys, Melissa L. 1999. “The Dimensionality of Deviant Employee Behavior in the Workplace.” Minneapolis, MN: University of Minnesota. http://oai.dtic.mil/oai/oai?verb=getRecord&metadataPrefix=html&identifier=ADA370788.

Gruys, Melissa L., and Paul R. Sackett. 2003. “Investigating the Dimensionality of Counterproductive Work Behavior.” *International Journal of Selection and Assessment* 11 (1): 30–42. https://doi.org/10.1111/1468-2389.00224.

Gruys, Melissa L., Susan M. Stewart, and Nathan A. Bowling. 2010. “Choosing to Report: Characteristics of Employees Who Report the Counterproductive Work Behavior of Others.” *International Journal of Selection and Assessment* 18 (4): 439–46. https://doi.org/10.1111/j.1468-2389.2010.00526.x.

Hamburger, M.E, K. C Basile, and A Vivolo. 2011. “Measuring Bullying and Bystander Experiences: A Compendium of Assessment Tools.” *Centers for Disease Control and Prevention*. Atlanta, Georgia. https://www.cdc.gov/violenceprevention/pdf/bullycompendium-a.pdf.

Harris, Lloyd C., and Emmanuel Ogbonna. 2002. “Exploring Service Sabotage.” *Journal of Service Research* 4 (3): 163–83. https://doi.org/10.1177/1094670502004003001.

Hauge, Lars Johan, Anders Skogstad, and Ståle Einarsen. 2009. “Individual and Situational Predictors of Workplace Bullying: Why Do Perpetrators Engage in the Bullying of Others?” *Work & Stress* 23 (4): 349–58. https://doi.org/10.1080/02678370903395568.

Hausknecht, John P., and Michael C. Sturman. 2011. “Justice as a Dynamic Construct: Effects of Individual Trajectories on Distal Work Outcomes.” *Journal of Applied Psychology* 96 (4): 872–80. https://psytopaca.asso-web.com/uploaded/fair-today-fair-tomorrow-holtz-and-harold-jap-2009.pdf.

Health and safety department US. 2016. “Department of Labor: OSHA 3148-06R 2016.” *OSHA Report*. https://www.osha.gov/laws-regs/federalregister/2016-12-07.

Henry, Nicola, and Anastasia Powell. 2016. “Sexual Violence in the Digital Age.” *Social & Legal Studies* 25 (4): 397–418. https://doi.org/10.1177/0964663915624273.

Hershcovis, M Sandy, and Julian Barling. 2007. “Towards a Relational Model of Workplace Aggression.” In *Research Companion to the Dysfunctional Workplace*, edited by J Langan-Fox, Cary Lynn Cooper, and R J Klimoski, Edward Elg, 268–84. Cheltenham, UK: Edward Elgar Publishing. https://doi.org/10.4337/9781847207081.00024.

Hitlan, Robbert T, Rebecca J. Cliffton, and Catherine M DeSoto. 2006. “Perceived Exclusion in the Workplace: The Moderating Effects of Gender on Workrelated Attitudes and Psychological Health.” *North American Journal of Psychology* 8 (2): 217–36. https://doi.org/228079.

Hitlan, Robert T., and Jennifer Noel. 2009. “The Influence of Workplace Exclusion and Personality on Counterproductive Work Behaviours: An Interactionist Perspective.” *European Journal of Work and Organizational Psychology* 18 (4): 477–502. https://doi.org/10.1080/13594320903025028.

Hobfoll, Stevan E, and A Shirom. 2000. “Conservation of Resources Theory: Applications to Stress and Management in the Workplace.” In *Handbook of Organizational Behavior*, edited by Robert T Golembiewski, 2nd ed., 57–81. New York: Marcel Dekker.

Hoel, H, S I Giga, and M J Davidson. 2007. “Expectations and Realities of Student Nurses’ Experiences of Negative Behaviour and Bullying in Clinical Placement and the Influences of Socialization Processes.” *Health Services Management Research* 20 (4): 270–78. https://doi.org/10.1258/095148407782219049.

Hollinger, Richard C. 1986. “Acts against the Workplace: Social Bonding and Employee Deviance.” *Deviant Behavior* 7 (1): 53–75. https://doi.org/10.1080/01639625.1986.9967695.

Holmes, Malcolm D., and Brad W. Smith. 2012. “Intergroup Dynamics of Extra-Legal Police Aggression: An Integrated Theory of Race and Place.” *Aggression and Violent Behavior* 17 (4): 344–53. https://doi.org/10.1016/j.avb.2012.03.006.

Holtz, Brian C., and Crystal M. Harold. 2009. “Fair Today, Fair Tomorrow? A Longitudinal Investigation of Overall Justice Perceptions.” *Journal of Applied Psychology* 94 (5): 1185–99. https://doi.org/10.1037/a0015900.

Hoskins, Carol Noll. 1983. “Psychometrics in Nursing Research—Further Development of the Interpersonal Conflict Scale.” *Research in Nursing & Health* 6 (2): 75–83. https://doi.org/10.1002/nur.4770060207.

Hubert, Adrienne B. 2002. “Mobbing. Een Extreme Vorm van Sociale Stress (Mobbing. An Extreme Type of Stress).” Gedownload op 24/05/06 van http://home.tiscali.nl/pesten.werk.

Hubert, Adrienne B., and J Furda. 1996. “Leidse Mobbing Schaal-II (LEMS-II; Geïndividualiseerde Versie (Leiden Mobbing Scale-II (LEMS-II Individualised Version).” Leiden University.

Hutchinson, Marie. 2013. “Bullying as Workgroup Manipulation: A Model for Understanding Patterns of Victimization and Contagion within the Workgroup.” *Journal of Nursing Management* 21 (3): 563–71. https://doi.org/10.1111/j.1365-2834.2012.01390.x.

Industrial Relations Services. 1999. “Harassment Policies, Study 662.” London.

Jaarsveld, Danielle D. van, David D. Walker, and Daniel P. Skarlicki. 2010. “The Role of Job Demands and Emotional Exhaustion in the Relationship between Customer and Employee Incivility.” *Journal of Management* 36 (6): 1486–1504. https://doi.org/10.1177/0149206310368998.

Jensen, Jaclyn M., Pankaj C. Patel, and Jana L. Raver. 2014. “Is It Better to Be Average? High and Low Performance as Predictors of Employee Victimization.” *Journal of Applied Psychology* 99 (2): 296–309. https://doi.org/10.1037/a0034822.

Johnson, Carolina, and Kathleen Otto. 2019. “Better Together: A Model for Women and LGBTQ Equality in the Workplace.” *Frontiers in Psychology* 10 (FEB): 1–17. https://doi.org/10.3389/fpsyg.2019.00272.

Johnson, Pamela R., and Julie Indvik. 2001. “Rudeness at Work: Impulse over Restraint.” *Public Personnel Management* 30 (4): 457–65. https://doi.org/10.1177/009102600103000403.

Jones, E. E. 1990. *Interpersonal Perception*. New York: Freeman.

Kaukiainen, Ari, Christina Salmivalli, Kaj Björkqvist, Karin Österman, Auli Lahtinen, Anne Kostamo, and Kirsti Lagerspetz. 2001. “Overt and Covert Aggression in Work Settings in Relation to the Subjective Well‐being of Employees.” *Aggressive Behavior* 27 (5): 360–71. https://doi.org/10.1002/ab.1021.abs.

Kavitha, S, and R Bhuvaneswari. 2016. “Impact of Social Media on Millenials- a Conceptual Study.” *Journal of Management Sciences and Technology* 4 (1): 80–86. https://www.apeejay.edu/aitsm/journal/docs/issue-oct-2016/ajmst040108.pdf.

Keashly, Loraleigh, and Steve Harvey. 2005. “Emotional Abuse in the Workplace.” In *Counterproductive Work Behavior: Investigations of Actors and Targets.*, 201–35. Washington: American Psychological Association. https://doi.org/10.1037/10893-009.

Keashly, Loraleigh, and Karen Jagatic. 2010. “North American Perspectives on Hostile Behaviors and Bullying at Work.” In *Bullying and Harassment in the Workplace*, 41–71. CRC Press. https://doi.org/10.1201/EBK1439804896-4.

Kerckhove, Johan Van De. 1993. “De Dynamiek in Het Belgisch Preventiebeleid.” *Tijdschrift Voor Arbeidsvraagstukken* 9 (1): 40–50.

Kern, Julie H., and Alicia A. Grandey. 2009. “Customer Incivility as a Social Stressor: The Role of Race and Racial Identity for Service Employees.” *Journal of Occupational Health Psychology* 14 (1): 46–57. https://doi.org/10.1037/a0012684.

Khan, Abdul Karim, Samina Quratulain, and Chris M. Bell. 2014. “Episodic Envy and Counterproductive Work Behaviors: Is More Justice Always Good?” *Journal of Organizational Behavior* 35 (1): 128–44. https://doi.org/10.1002/job.1864.

Khoo, S. B. 2010. “Academic Mobbing: Hidden Health Hazard at Workplace.” *Malaysian Family Physician* 5 (2): 61–67. https://www.ncbi.nlm.nih.gov/pmc/articles/PMC4170397/.

Kisa, Sezer. 2008. “Turkish Nurses’ Experiences of Verbal Abuse at Work.” *Archives of Psychiatric Nursing* 22 (4): 200–207. https://doi.org/10.1016/j.apnu.2007.06.013.

Klein, Jessie. 2006. “Cultural Capital and High School Bullies.” *Men and Masculinities* 9 (1): 53–75. https://doi.org/10.1177/1097184X04271387.

Konik, Julie, and Lilia M. Cortina. 2008. “Policing Gender at Work: Intersections of Harassment Based on Sex and Sexuality.” *Social Justice Research* 21 (3): 313–37. https://doi.org/10.1007/s11211-008-0074-z.

Krieger, Nancy, Pamela D. Waterman, Cathy Hartman, Lisa M. Bates, Anne M. Stoddard, Margaret M. Quinn, Glorian Sorensen, and Elizabeth M. Barbeau. 2006. “Social Hazards on the Job: Workplace Abuse, Sexual Harassment, and Racial Discrimination—a Study of Black, Latino, and White Low-Income Women and Men Workers in the United States.” *International Journal of Health Services* 36 (1): 51–85. https://doi.org/10.2190/3EMB-YKRH-EDJ2-0H19.

Lagerspetz, Kirsti M. J., Kaj Björkqvist, and Tarja Peltonen. 1988. “Is Indirect Aggression Typical of Females? Gender Differences in Aggressiveness in 11- to 12-Year-Old Children.” *Aggressive Behavior* 14 (6): 403–14. https://doi.org/10.1002/1098-2337(1988)14:6<403::AID-AB2480140602>3.0.CO;2-D.

Lange, Stefanie, Hermann Burr, Paul Maurice Conway, and Uwe Rose. 2019. “Workplace Bullying among Employees in Germany: Prevalence Estimates and the Role of the Perpetrator.” *International Archives of Occupational and Environmental Health* 92 (2): 237–47. https://doi.org/10.1007/s00420-018-1366-8.

Leafloor, D, and J Biggs. 1993. “Management of Abusive Behaviour in a Hemodialysis Unit.” *The Journal of the Canadian Association of Nephrology Nurses and Technician* 3 (1): 19–20.

Lee, Raymond T., and Céleste M. Brotheridge. 2006. “When Prey Turns Predatory: Workplace Bullying as a Predictor of Counteraggression/Bullying, Coping, and Well-Being.” *European Journal of Work and Organizational Psychology* 15 (3): 352–77. https://doi.org/10.1080/13594320600636531.

Leiter, Michael P., Heather K. Spence Laschinger, Arla Day, and Debra Gilin Oore. 2011. “The Impact of Civility Interventions on Employee Social Behavior, Distress, and Attitudes.” *Journal of Applied Psychology* 96 (6): 1258–74. https://doi.org/10.1037/a0024442.

Lemert, Edwin M. 1997. *The Trouble with Evil: Social Control at the Edge of Morality*. Albany: State University of New York Press.

Leon-Perez, Jose M., Francisco J. Medina, Alicia Arenas, and Lourdes Munduate. 2015. “The Relationship between Interpersonal Conflict and Workplace Bullying.” *Journal of Managerial Psychology* 30 (3): 250–63. https://doi.org/10.1108/JMP-01-2013-0034.

Leskinen, Emily A., and Lilia M. Cortina. 2014. “Dimensions of Disrespect.” *Psychology of Women Quarterly* 38 (1): 107–23. https://doi.org/10.1177/0361684313496549.

Lewis, Duncan, and Rod Gunn. 2007. “Workplace Bullying in the Public Sector: Understanding the Racial Dimension.” *Public Administration* 85 (3): 641–65. https://doi.org/10.1111/j.1467-9299.2007.00665.x.

Lewis, Sian E. 2006. “Recognition of Workplace Bullying: A Qualitative Study of Women Targets in the Public Sector.” *Journal of Community & Applied Social Psychology* 16 (2): 119–35. https://doi.org/10.1002/casp.850.

Leymann, Heinz. 1990. “Mobbing and Psychological Terror at Workplaces.” *Violence and Victims* 5: 119–26. https://www.mobbingportal.com/LeymannV&V1990(3).pdf.

———. 1996. “The Content and Development of Mobbing at Work.” *European Journal of Work and Organizational Psychology* 5 (2): 165–84. https://doi.org/10.1080/13594329608414853.

Leymann, Heinz, and Klaus Niedl. 1994. *Mobbing. Psychoterror Am Arbeitsplatz. Ein Ratgeber Für Betroffene (Mobbing. Psychoterror at Work. A Guide for Targets)*. Wien: Verl. des Österr. Gewerkschaftsbundes. https://www.worldcat.org/title/mobbing-psychoterror-am-arbeitsplatz-ein-ratgeber-fur-betroffene/oclc/75519967.

Lim, Sandy, Lilia M. Cortina, and Vicki J. Magley. 2008. “Personal and Workgroup Incivility: Impact on Work and Health Outcomes.” *Journal of Applied Psychology* 93 (1): 95–107. https://doi.org/10.1037/0021-9010.93.1.95.

Lim, Vivien K. 2002. “The IT Way of Loafing on the Job: Cyberloafing, Neutralizing and Organizational Justice.” *Journal of Organizational Behavior* 23 (5): 675–94. https://doi.org/10.1002/job.161.

Litzky, Barrie E, Kimberly A Eddleston, and Deborah L Kidder. 2006. “The Good, the Bad, and the Misguided: How Managers Inadvertently Encourage Deviant Behaviors.” *Academy of Management Perspectives* 20 (1): 91–103. https://doi.org/10.5465/amp.2006.19873411.

Lutgen-Sandvik, Pamela. 2003. “The Communicative Cycle of Employee Emotional Abuse.” *Management Communication Quarterly* 16 (4): 471–501. https://doi.org/10.1177/0893318903251627.

Maccallum, E. J. 2002. “Othering and Psychiatric Nursing.” *Journal of Psychiatric and Mental Health Nursing* 9 (1): 87–94. https://doi.org/10.1046/j.1351-0126.2001.00449.x.

Macdonald, Grant, and Frank Sirotich. 2005. “Violence in the Social Work Workplace.” *International Social Work* 48 (6): 772–81. https://doi.org/10.1177/0020872805057087.

Magee, Joe C., and Adam D. Galinsky. 2008. “Social Hierarchy: The Self‐reinforcing Nature of Power and Status.” *Academy of Management Annals* 2 (1): 351–98. https://doi.org/10.5465/19416520802211628.

Maran, Daniela Acquadro, Claudio Giovanni Cortese, Pierluigi Pavanelli, Giulio Fornero, and Maria Michela Gianino. 2019. “Gender Differences in Reporting Workplace Violence: A Qualitative Analysis of Administrative Records of Violent Episodes Experienced by Healthcare Workers in a Large Public Italian Hospital.” *BMJ Open* 9 (11): e031546. https://doi.org/10.1136/bmjopen-2019-031546.

Marcus, Bernd, Heinz Schuler, Particia Quell, and Gerhardt Humpfner. 2002. “Measuring Counterproductivity: Development and Initial Validation of a German Self-Report Questionnaire.” *International Journal of Selection and Assessment* 10 (1&2): 18–35. https://doi.org/10.1111/1468-2389.00191.

Martin, Roberta J., and Donald W. Hine. 2005. “Development and Validation of the Uncivil Workplace Behavior Questionnaire.” *Journal of Occupational Health Psychology* 10 (4): 477–90. https://doi.org/10.1037/1076-8998.10.4.477.

Martinez, Marc A., Amos Zeichner, Dennis E. Reidy, and Joshua D. Miller. 2008. “Narcissism and Displaced Aggression: Effects of Positive, Negative, and Delayed Feedback.” *Personality and Individual Differences* 44 (1): 140–49. https://doi.org/10.1016/j.paid.2007.07.012.

Martino, Vittorio Di. 2009. “Workplace Violence in the Health Sector Country Case Studies Brazil, Bulgaria, Lebanon, Portugal, South Africa, Thailand and an Additional Australian Study. Synthesis Report.” https://www.who.int/violence_injury_prevention/violence/activities/workplace/WVsynthesisreport.pdf.

Matthiesen, Stig Berge, and Ståle Einarsen. 2007. “Harassment in the Workplace and the Victimization of Men.” *Violence and Victims* 22 (6): 85–105. https://doi.org/10.1891/0886-6708.12.3.247.

Mawritz, Mary Bardes, David M. Mayer, Jenny M. Hoobler, Sandy J. Wayne, and Sophia V. Marinova. 2012. “A Trickle-down Model of Abusive Supervision.” *Personnel Psychology* 65 (2): 325–57. https://doi.org/10.1111/j.1744-6570.2012.01246.x.

Mayhew, Claire, Paul McCarthy, Duncan Chappell, Michael Quinlan, Michelle Barker, and Michael Sheehan. 2004. “Measuring the Extent of Impact from Occupational Violence and Bullying on Traumatised Workers.” *Employee Responsibilities and Rights Journal* 16 (3): 117–34. https://doi.org/10.1023/B:ERRJ.0000038648.08568.46.

Ménard, Julie, Luc Brunet, and André Savoie. 2011. “Interpersonal Workplace Deviance: Why Do Offenders Act out? A Comparative Look on Personality and Organisational Variables.” *Canadian Journal of Behavioural Science / Revue Canadienne Des Sciences Du Comportement* 43 (4): 309–17. https://doi.org/10.1037/a0024741.

Milam, Alex C., Christiane Spitzmueller, and Lisa M. Penney. 2009. “Investigating Individual Differences among Targets of Workplace Incivility.” *Journal of Occupational Health Psychology* 14 (1): 58–69. https://doi.org/10.1037/a0012683.

Mitchell, Marie S., and Maureen L. Ambrose. 2007. “Abusive Supervision and Workplace Deviance and the Moderating Effects of Negative Reciprocity Beliefs.” *Journal of Applied Psychology* 92 (4): 1159–68. https://doi.org/10.1037/0021-9010.92.4.1159.

Moore, Mark E., Alison M. Konrad, Yang Yang, Eddy S.W. Ng, and Alison J. Doherty. 2011. “The Vocational Well-Being of Workers with Childhood Onset of Disability: Life Satisfaction and Perceived Workplace Discrimination.” *Journal of Vocational Behavior* 79 (3): 681–98. https://doi.org/10.1016/j.jvb.2011.03.019.

Nagle, Joelle. 2018. “Twitter, Cyber-Violence, and the Need for a Critical Social Media Literacy in Teacher Education: A Review of the Literature.” *Teaching and Teacher Education* 76 (September): 86–94. https://doi.org/10.1016/j.tate.2018.08.014.

Namie, Gary. 2003. “Workplace Bullying: Escalated Incivility.” *Ivey Business Journal Online* nov/dec: 1–6.

Namie, Gary, and Ruth F. Namie. 2011. *Bully Free Workplace. Stop Jerks, Weasels, and Snakes from Killing Your Organization*. John Wiley And Sons Ltd. https://www.amazon.com/Bully-Free-Workplace-Weasels-Killing-Organization/dp/0470942207.

Neall, Annabelle M., and Michelle R. Tuckey. 2014. “A Methodological Review of Research on the Antecedents and Consequences of Workplace Harassment.” *Journal of Occupational and Organizational Psychology* 87 (2): 225–57. https://doi.org/10.1111/joop.12059.

Neese, William T, O. C. Ferrell, and Linda Ferrell. 2003. “An Analysis to Mail and Wire Fraud: Cases Related to Marketing Communication: Implications for Corporate Citizenship.”

Nelen, Wendy, Wouter De Wit, Milou Golbach, Loes Van Druten, Chaja Deen, and Ron Scholte. 2018. “Sociale Veiligheid in En Rond Scholen. Primair (Speciaal) Onderwijs 2010-2018. Voortgezet (Speciaal) Onderwijs 2006-2018 (Social Safety in and around Schools. Primary (Special) Education 2010-2018. Secondary (Special) Education 2006-2018).” Nijmegen: Praktikon BV. https://www.rijksoverheid.nl/documenten/rapporten/2018/12/21/monitor-sociale-veiligheid.

Neuman, Joel H., and Robert A. Baron. 1998. “Workplace Violence and Workplace Aggression: Evidence Concerning Specific Forms, Potential Causes, and Preferred Targets.” *Journal of Management* 24 (3): 391–419. https://doi.org/10.1177/014920639802400305.

———. 2005. “Aggression in the Workplace a Social- Psychological Perspective.” In *Counterproductive Work Behavior; Investigations of Actors and Targets*, edited by Suzy Fox and Paul E. Spector, 13–40. Washington, DC, US.: American Psychological Association. https://doi.org/https://doi.org/10.1037/10893-000.

Nielsen, Morten Birkeland, and Ståle Valvatne Einarsen. 2018. “What We Know, What We Do Not Know, and What We Should and Could Have Known about Workplace Bullying: An Overview of the Literature and Agenda for Future Research.” *Aggression and Violent Behavior* 42 (July): 71–83. https://doi.org/10.1016/j.avb.2018.06.007.

Nielsen, Morten Birkeland, Stig Berge Matthiesen, and Ståle Einarsen. 2008. “Sense of Coherence as a Protective Mechanism among Targets of Workplace Bullying.” *Journal of Occupational Health Psychology* 13 (2): 128–36. https://doi.org/10.1037/1076-8998.13.2.128.

Nielsen, Morten Birkeland, Tone Tangen, Thormod Idsoe, Stig Berge Matthiesen, and Nils Magerøy. 2015. “Post-Traumatic Stress Disorder as a Consequence of Bullying at Work and at School. A Literature Review and Meta-Analysis.” *Aggression and Violent Behavior* 21 (March): 17–24. https://doi.org/10.1016/j.avb.2015.01.001.

Okechukwu, Cassandra A., Kerry Souza, Kelly D. Davis, and A. Butch de Castro. 2014. “Discrimination, Harassment, Abuse, and Bullying in the Workplace: Contribution of Workplace Injustice to Occupational Health Disparities.” *American Journal of Industrial Medicine* 57 (5): 573–86. https://doi.org/10.1002/ajim.22221.

Park, Eun, Mikyoung Lee, and Myungsook Park. 2017. “Instruments and Taxonomy of Workplace Bullying in Health Care Organizations.” *Asian Nursing Research* 11 (4): 237–45. https://doi.org/10.1016/j.anr.2017.10.001.

Park, Young, Charlotte Fritz, and Steve Jex. 2018. “Daily Cyber Incivility and Distress: The Moderating Roles of Resources at Work and Home.” *Journal of Management* 44 (7): 2535–57. https://doi.org/10.1177/0149206315576796.

Pearcey, Patricia A, and Barbara E Elliott. 2004. “Student Impressions of Clinical Nursing.” *Nurse Education Today* 24 (5): 382–87. https://doi.org/10.1016/j.nedt.2004.03.007.

Pearson, Christine, Lynne Andersson, and Christine Porath. 2005. “Workplace Incivility.” In *Counterproductive Workplace Behavior: Investigations of Actors and Targets*, edited by S. Fox and Paul E Spector, 177–200. Washington, D.C.: Adlerian Psychology Associates.

Pearson, Christine M., Lynne M. Andersson, and Judith W. Wegner. 2001. “When Workers Flout Convention: A Study of Workplace Incivility.” *Human Relations* 54 (11): 1387–1419. https://doi.org/10.1177/00187267015411001.

Peng, He. 2012. “Counterproductive Work Behavior among Chinese Knowledge Workers.” *International Journal of Selection and Assessment* 20 (2): 119–38. https://doi.org/10.1111/j.1468-2389.2012.00586.x.

Penney, Lisa M., and Paul E. Spector. 2005. “Job Stress, Incivility, and Counterproductive Work Behavior (CWB): The Moderating Role of Negative Affectivity.” *Journal of Organizational Behavior* 26 (7): 777–96. https://doi.org/10.1002/job.336.

Perry, Elissa L., and Lisa M. Finkelstein. 1999. “Toward a Broader View of Age Discrimination in Employment-Related Decisions: A Joint Consideration of Organizational Factors and Cognitive Processes.” *Human Resource Management Review* 9 (1): 21–49. https://doi.org/10.1016/S1053-4822(99)00010-8.

Pfuhl, Erdwin H., and Stuart Henry. 1993. *The Deviance Process*. 3rd ed. New York: Transaction Publishers.

Pina, Afroditi, Theresa A. Gannon, and Benjamin Saunders. 2009. “An Overview of the Literature on Sexual Harassment: Perpetrator, Theory, and Treatment Issues.” *Aggression and Violent Behavior* 14 (2): 126–38. https://doi.org/10.1016/j.avb.2009.01.002.

Poole, Chris. 2010. *If Your Colleague Is Abused at Home: Dealing with Domestic Violence in the Workplace*. EU Daphne.

Porath, Christine L., and Christine M. Pearson. 2012. “Emotional and Behavioral Responses to Workplace Incivility and the Impact of Hierarchical Status.” *Journal of Applied Social Psychology* 42 (Suppl. 1): E326–57. https://doi.org/10.1111/j.1559-1816.2012.01020.x.

Quine, Lyn. 2001. “Workplace Bullying in Nurses.” *Journal of Health Psychology* 6 (1): 73–84. https://doi.org/10.1177/135910530100600106.

Raab, J. 2003. “Dark Networks as Problems.” *Journal of Public Administration Research and Theory* 13 (4): 413–39. https://doi.org/10.1093/jopart/mug029.

Ransley, Janet, and Lorraine Mazerolle. 2009. “Policing in an Era of Uncertainty.” *Police Practice and Research* 10 (4): 365–81. https://doi.org/10.1080/15614260802586335.

Raver, J. L., and L. H. Nishii. 2010. “Once, Twice, or Three Times as Harmful? Ethnic Harassment, Gender Harassment, and Generalized Workplace Harassment.” *Journal of Applied Psychology* 95 (2): 236–54. https://doi.org/10.1037/a0018377.

Reichert, Tom, and Courtney Carpenter. 2004. “An Update on Sex in Magazine Advertising: 1983 to 2003.” *Journalism & Mass Communication Quarterly* 81 (4): 823–37. https://doi.org/10.1177/107769900408100407.

Reijntjes, Albert, Jan H. Kamphuis, Peter Prinzie, and Michael J. Telch. 2010. “Peer Victimization and Internalizing Problems in Children: A Meta-Analysis of Longitudinal Studies.” *Child Abuse & Neglect* 34 (4): 244–52. https://doi.org/10.1016/j.chiabu.2009.07.009.

Reyns, Bradford W., Billy Henson, and Bonnie S. Fisher. 2012. “Stalking in the Twilight Zone: Extent of Cyberstalking Victimization and Offending among College Students.” *Deviant Behavior* 33 (1): 1–25. https://doi.org/10.1080/01639625.2010.538364.

Rickett, Bridgette, and Andrew Roman. 2012. “‘Heroes and Matriarchs’: Working-Class Femininities, Violence and Door Supervision Work.” *Gender, Work & Organization* 20 (6): n/a-n/a. https://doi.org/10.1111/gwao.12002.

Riketta, Michael. 2005. “Organizational Identification: A Meta-Analysis.” *Journal of Vocational Behavior* 66 (2): 358–84. https://doi.org/10.1016/j.jvb.2004.05.005.

Robinson, Richard N.S. 2008. “Revisiting Hospitality’s Marginal Worker Thesis: A Mono-Occupational Perspective.” *International Journal of Hospitality Management* 27 (3): 403–13. https://doi.org/10.1016/j.ijhm.2007.09.003.

Robinson, Sandra L., and Rebecca J. Bennett. 1995. “A Typology of Deviant Workplace Behaviors: A Multidimensional Scaling Study.” *Academy of Management Journal* 38 (2): 555–72. https://doi.org/10.5465/256693.

Rominiecka, Marta. 2008. “Non-Verbal Cues in Politics: An Analysis of Gestural Signals Sent by American and European Politicians.” *Poznań Studies in Contemporary Linguistics* 44 (2). https://doi.org/10.2478/v10010-008-0012-0.

Rosewarne, Lauren. 2007. “Pin-Ups in Public Space.” *Women’s Studies International Forum* 30 (4): 313–25. https://doi.org/10.1016/j.wsif.2007.05.003.

Rospenda, Kathleen M, Judith A Richman, and Candice A Shannon. 2009. “Prevalence and Mental Health Correlates of Harassment and Discrimination in the Workplace.” *Journal of Interpersonal Violence* 24 (5): 819–43. https://doi.org/10.1177/0886260508317182.

Ruiz-Hernández, José Antonio, Cecilia López-García, Bartolomé Llor-Esteban, Inmaculada Galián-Muñoz, and Ana Pilar Benavente-Reche. 2016. “Evaluation of the Users Violence in Primary Health Care: Adaptation of an Instrument.” *International Journal of Clinical and Health Psychology* 16 (3): 295–305. https://doi.org/10.1016/j.ijchp.2016.06.001.

Sakurai, Kenji, and Steve M. Jex. 2012. “Coworker Incivility and Incivility Targets’ Work Effort and Counterproductive Work Behaviors: The Moderating Role of Supervisor Social Support.” *Journal of Occupational Health Psychology* 17 (2): 150–61. https://doi.org/10.1037/a0027350.

Scarduzio, Jennifer A., and Patricia Geist-Martin. 2010. “Accounting for Victimization: Male Professors’ Ideological Positioning in Stories of Sexual Harassment.” *Management Communication Quarterly* 24 (3): 419–45. https://doi.org/10.1177/0893318909358746.

Schein, Edgar H. 2010. “The Three Levels of Culture.” In *Organizational Culture and Leadership*, 5th ed., 8. Josey-Bass. http://my.safaribooksonline.com/book/leadership/9780470190609.

Schilpzand, Pauline, Irene E. De Pater, and Amir Erez. 2016. “Workplace Incivility: A Review of the Literature and Agenda for Future Research.” *Journal of Organizational Behavior* 37 (February): S57–88. https://doi.org/10.1002/job.1976.

Schneider, Kimberly T., Robert T. Hitlan, and Phanikiran Radhakrishnan. 2000. “An Examination of the Nature and Correlates of Ethnic Harassment Experiences in Multiple Contexts.” *Journal of Applied Psychology* 85 (1): 3–12. https://doi.org/10.1037/0021-9010.85.1.3.

Schneider, Kimberly T., Eric D. Wesselmann, and Eros R. DeSouza. 2017. “Confronting Subtle Workplace Mistreatment: The Importance of Leaders as Allies.” *Frontiers in Psychology* 8 (JUN): 8–11. https://doi.org/10.3389/fpsyg.2017.01051.

Schuilenburg, M. B., A Coenraads, and P Van Calster. 2009. “Onder de Mensen. De Aanpak van Transportcriminaliteit Door Politie, Verzekeraars En Schade-Experts. (Among People. The Approach to Transport Crime by the Police, Insurers and Damage Experts).” *Justitiële Verkenningen* 35 (1): 43–63. https://search.proquest.com/openview/4fabe5000c856439826a19cfdc2045bc.

Scott, Kristin L., Simon Lloyd D. Restubog, and Thomas J. Zagenczyk. 2013. “A Social Exchange-Based Model of the Antecedents of Workplace Exclusion.” *Journal of Applied Psychology* 98 (1): 37–48. https://doi.org/10.1037/a0030135.

Serenko, Alexander. 2019. “Knowledge Sabotage as an Extreme Form of Counterproductive Knowledge Behavior: Conceptualization, Typology, and Empirical Demonstration.” *Journal of Knowledge Management* 23 (7): 1260–88. https://doi.org/10.1108/JKM-01-2018-0007.

Sexton, Patricia Cayo, and Carroll M. Brodsky. 1977. “The Harassed Worker.” *Industrial and Labor Relations Review* 31 (1): 123. https://doi.org/10.2307/2522527.

Shahtahmasebi, Said. 2004. “Quality of Life: A Case Report of Bullying in the Workplace.” *The Scientific World Journal* 4: 118–23. https://doi.org/10.1100/tsw.2004.13.

Shallcross, L., M. Sheehan, and S. Ramsay. 2008. “WORKPLACE MOBBING : EXPERIENCES IN THE PUBLIC SECTOR.” *International Journal of Organisational Behaviour, 13(2),* 13 (2): 58–70. https://eprints.qut.edu.au/43891/.

Silva João, Ana Lúcia da, and António Fernando Saldanha Portelada. 2019. “Mobbing and Its Impact on Interpersonal Relationships at the Workplace.” *Journal of Interpersonal Violence* 34 (13): 2797–2812. https://doi.org/10.1177/0886260516662850.

Skarlicki, Daniel P, and Robert Folger. 1997. “Retaliation in the Workplace: The Roles of Distributive, Procedural, and Interactional Justice.” *Journal of Applied Psychology* 82 (3): 434–43. https://doi.org/10.1037/0021-9010.82.3.434.

Slavin, S., C. Batrouney, and D. Murphy. 2007. “Fear Appeals and Treatment Side-Effects: An Effective Combination for HIV Prevention?” *AIDS Care* 19 (1): 130–37. https://doi.org/10.1080/09540120600866473.

Sliter, Katherine A., Michael T. Sliter, Scott A. Withrow, and Steve M. Jex. 2012. “Employee Adiposity and Incivility: Establishing a Link and Identifying Demographic Moderators and Negative Consequences.” *Journal of Occupational Health Psychology* 17 (4): 409–24. https://doi.org/10.1037/a0029862.

Smith, Peter K. 2009. “Cyberbullying.” *Zeitschrift Für Psychologie / Journal of Psychology* 217 (4): 180–81. https://doi.org/10.1027/0044-3409.217.4.180.

Snijder, James, Lynn Schrepferman, Jessica Oeser, Gerald Patterson, Mike Stoolmiller, Kassy Johnson, and ABIGAIL SNYDER. 2005. “Deviancy Training and Association with Deviant Peers in Young Children: Occurrence and Contribution to Early-Onset Conduct Problems.” *Development and Psychopathology* 17 (02): 397–413. https://doi.org/10.1017/S0954579405050194.

Snyder, Lori Anderson, Jennifer S. Carmichael, Lauren V. Blackwell, Jeanette N. Cleveland, and George C. Thornton. 2010. “Perceptions of Discrimination and Justice among Employees with Disabilities.” *Employee Responsibilities and Rights Journal* 22 (1): 5–19. https://doi.org/10.1007/s10672-009-9107-5.

Sobre-Denton, Miriam Shoshana. 2012. “Stories from the Cage.” *Journal of Contemporary Ethnography* 41 (2): 220–50. https://doi.org/10.1177/0891241611429301.

Spector, Paul E. 1998. “A Control Model of the Job Stress Process.” In *Theories of Organizational Stress*, edited by C L Cooper, 153–69. New York: Oxford University Press.

Spector, Paul E., Suzy Fox, Lisa M. Penney, Kari Bruursema, Angeline Goh, and Stacey Kessler. 2006. “The Dimensionality of Counterproductivity: Are All Counterproductive Behaviors Created Equal?” *Journal of Vocational Behavior* 68 (3): 446–60. https://doi.org/10.1016/j.jvb.2005.10.005.

Spector, Paul E., and Zhiqing E. Zhou. 2014. “The Moderating Role of Gender in Relationships of Stressors and Personality with Counterproductive Work Behavior.” *Journal of Business and Psychology* 29 (4): 669–81. https://doi.org/10.1007/s10869-013-9307-8.

Spector, Paul E, Suzy Fox, and Theresa Domagalski. 2006. “Emotions, Violence, and Counterproductive Work Behavior.” In *Handbook OfWorkplace Violence*, edited by Kevin E Kelloway, Julian Barling, and J Hurrell, 29–46. Thousand Oaks CA: Sage.

Spector, Paul E, and Steve M. Jex. 1998. “Development of Four Self-Report Measures of Job Stressors and Strain: Interpersonal Conflict at Work Scale, Organizational Constraints Scale, Quantitative Workload Inventory, and Physical Symptoms Inventory.” *Journal of Occupational Health Psychology* 3 (4): 356–87. https://psycnet.apa.org/buy/1998-12418-005.

Stafford, Thomas F., and Andrew Urbaczewski. 2004. “Spyware: The Ghost in the Machine.” *Communications of the Association for Information Systems* 14: 291–306. http://130.18.86.27/faculty/warkentin/SecurityPapers/Merrill/StaffordUrbaczewski2004_CAIS14_Spyware.pdf.

Steinberg, Matthew P, Elaine Allensworth, and David W. Johnson. 2011. *Student and Teacher Safety in Chicago Public Schools: The Roles of Community Context and School Social Organization*. Chicago: Consortium on Chicago School Research. https://doi.org/ISBN: 978098450764151500.

Stewart, Susan M., Mark N. Bing, H. Kristl Davison, David J. Woehr, and Michael D. McIntyre. 2009. “In the Eyes of the Beholder: A Non-Self-Report Measure of Workplace Deviance.” *Journal of Applied Psychology* 94 (1): 207–15. https://doi.org/10.1037/a0012605.

Strandmark, M., and LR-M. Hallberg. 2007. “Being Rejected and Expelled from the Workplace: Experiences of Bullying in the Public Service Sector.” *Qualitative Research in Psychology* 4 (1–2): 1–14. https://doi.org/10.1080/14780880701473359.

Strandmark, Margaretha, and Gullbritt Rahm. 2014. “Development, Implementation and Evaluation of a Process to Prevent and Combat Workplace Bullying.” *Scandinavian Journal of Public Health* 42 (15_suppl): 66–73. https://doi.org/10.1177/1403494814549494.

Tepper, Bennett J. 2000. “Consequences of Abusive Supervision.” *Academy of Management Journal* 43 (2): 178–90. https://doi.org/10.5465/1556375.

———. 2007. “Abusive Supervision in Work Organizations: Review, Synthesis, and Research Agenda.” *Journal of Management* 33 (3): 261–89. https://doi.org/10.1177/0149206307300812.

Thomas, Sandra P., and Renee Burk. 2009. “Junior Nursing Students’ Experiences of Vertical Violence during Clinical Rotations.” *Nursing Outlook* 57 (4): 226–31. https://doi.org/10.1016/j.outlook.2008.08.004.

Towns, D M, and M S Johnson. 2003. “Sexual Harassment in the 21st Century - E-Harassment in the Workplace.” *Employee Relations Law Journal* 29 (June): 7–24. https://www.researchgate.net/publication/298232804_Sexual_harassment_in_the_21st_century_-_E-harassment_in_the_workplace.

Treadway, Darren C., Garry L. Adams, and Joseph M. Goodman. 2005. “The Formation of Political Sub-Climates: Predictions from Social Identity, Structuration, and Symbolic Interaction.” *Journal of Business and Psychology* 20 (2): 201–9. https://doi.org/10.1007/s10869-005-8259-z.

Treadway, Darren C., Gerald R. Ferris, Allison B. Duke, Garry L. Adams, and Jason B. Thatcher. 2007. “The Moderating Role of Subordinate Political Skill on Supervisors’ Impressions of Subordinate Ingratiation and Ratings of Subordinate Interpersonal Facilitation.” *Journal of Applied Psychology* 92 (3): 848–55. https://doi.org/10.1037/0021-9010.92.3.848.

Trembly. 2014. “E-Mails Nail Wrongdoers.” The Free Library. September 2014. https://doi.org/10.1002/imhj.20015.

Trépanier, Sarah-Geneviève, Claude Fernet, and Stéphanie Austin. 2013. “Workplace Psychological Harassment in Canadian Nurses: A Descriptive Study.” *Journal of Health Psychology* 18 (3): 383–96. https://doi.org/10.1177/1359105312443401.

Tsuno, Kanami, Norito Kawakami, Akizumi Tsutsumi, Akihito Shimazu, Akiomi Inoue, Yuko Odagiri, Toru Yoshikawa, Takashi Haratani, Teruichi Shimomitsu, and Ichiro Kawachi. 2015. “Socioeconomic Determinants of Bullying in the Workplace: A National Representative Sample in Japan.” Edited by Harry Zhang. *PLOS ONE* 10 (3): e0119435. https://doi.org/10.1371/journal.pone.0119435.

Underwood, M.K. 2002. “Sticks and Stones and Social Exclusion: Aggression among Girls and Boys.” In *Blackwell Handbook of Childhood Social Development*, edited by Peter K. Smith and Craig H. Hart, 1st ed., 387. Chichester: Blackwell Publishing Ltd. https://www.gacbe.ac.in/images/E books/Blackwell Handbook of Childhood Social Development.pdf#page=550.

Vallant, Sharon, and Stephen Neville. 2006. “The Relationship between Student Nurse and Nurse Clinician: Impact on Student Learning.” *Nursing Praxis in New Zealand* 22 (3): 23–33. https://go.gale.com/ps/anonymous?id=GALE%7CA160281698&sid=googleScholar&v=2.1&it=r&linkaccess=abs&issn=01127438&p=AONE&sw=w.

Vartia, Maarit A.L. 2001. “Consequences of Workplace Bullying with Respect to the Well-Being of Its Targets and the Observers of Bullying.” *Scandinavian Journal of Work, Environment & Health* 27 (1): 63–69. https://doi.org/10.5271/sjweh.588.

Vartia, Maarit A L. 1993. “Psychological Harassment (Bullying, Mobbing) at Work.” OECD Panel group on women, work, and health,.

Verschuren, C. M. 2009a. *Handleiding Sociale Veiligheidsindex: Sociale Veiligheid Binnen de Werkomgeving (Manual Social Safetyindex: Social Safety in the Working Environment )*. 1st ed. Amsterdam: Pearson.

———. 2009b. *Sociale Veiligheidsindex (SVI) ( Social Safety Index)- Questionnaire 154 Items*. 1st ed. Amsterdam: Pearson.

———. 2012. *Handleiding Sociale Veiligheidsindex: Sociale Veiligheid Binnen de Werkomgeving (Manual Social Safetyindex: Social Safety in the Working Environment )*. 2nd ed. Zeist: Kerckebosch. https://www.kerckebosch.nl/arbo-veiligheid/handleiding-sociale-veiligheidsindex-2832.

Vickers, Margaret H. 2014. “Workplace Bullying as Workplace Corruption.” *Administration & Society* 46 (8): 960–85. https://doi.org/10.1177/0095399713498750.

Vliert, Evert Van de. 2010. “Moving Bullies and Victims up on Conflict-Researchers’ Waiting Lists.” *Negotiation and Conflict Management Research* 3 (2): 87–90. https://doi.org/10.1111/j.1750-4716.2010.00055.x.

Vliert, Evert Van der. 1997. *Complex Interpersonal Conflict Behaviour*. 1st ed. London: Psychology Press. https://doi.org/10.4324/9780203776049.

Walsh, Benjamin M., Vicki J. Magley, David W. Reeves, Kimberly A. Davies-Schrils, Matthew D. Marmet, and Jessica A. Gallus. 2012. “Assessing Workgroup Norms for Civility: The Development of the Civility Norms Questionnaire-Brief.” *Journal of Business and Psychology* 27 (4): 407–20. https://doi.org/10.1007/s10869-011-9251-4.

waters, Harry. 1992. “Race, Culture and Interpersonal Conflict.” *International Journal of Intercultural Relations* 16 (4): 437–54. https://doi.org/10.1016/0147-1767(92)90032-P.

Weatherbee, Terrance G. 2010. “Counterproductive Use of Technology at Work: Information &amp; Communications Technologies and Cyberdeviancy.” *Human Resource Management Review* 20 (1): 35–44. https://doi.org/10.1016/j.hrmr.2009.03.012.

Weatherbee, Terrance Gordon. 2007. *Cyberaggression in the Workplace: Construct Development, Operationalization, and Measurement (Dissertation at Saint Mary’s University, Halifax)*. *ProQuest Dissertations and Theses*. https://search.proquest.com/docview/304719031?accountid=14504%0Ahttp://godot.lib.sfu.ca/GODOT/hold_tab.cgi?url_ver=Z39.88-2004&rft_val_fmt=info:ofi/fmt:kev:mtx:dissertation&genre=dissertations+%26+theses&sid=ProQ:ProQuest+Dissertations+%26+Theses+A%26I&at.

Weatherbee, Terrance, and E. Kelloway. 2006. “A Case of Cyberdeviancy: Cyberaggression in the Workplace.” In *Handbook of Workplace Violence*, edited by Kevin E Kelloway, Julian Barling, and J.J Hurrell, 445–88. 2455 Teller Road, Thousand Oaks California 91320 United States: SAGE Publications, Inc. https://doi.org/10.4135/9781412976947.n19.

Webb, P. Taylor. 2008. “Re‐mapping Power in Educational Micropolitics.” *Critical Studies in Education* 49 (2): 127–42. https://doi.org/10.1080/17508480802040183.

West, Sarah Myers, Kate Crawford, and Meredith Whittaker. 2019. “Discriminationg Systems: Gender, Race, and Power in AI.” New York. https://ainowinstitute.org/discriminatingsystems.pdf.

Williams, Kipling D. 1997. *Social Ostracism*. Edited by R. M Kowalski. New York: Plenium Press.

Williams, Kipling D., and Lisa Zadro. 2005. “Ostracism: The Indiscriminate Early Detection System.” In *The Social Outcast: Ostracism, Social Exclusion, Rejection, and Bullying*, edited by Kipling D. Williams, Joseph P. Forgas, and W Von Hippel, 19–34. New York: Psychology Press.

Williams, Kipling D, and Kristin L Sommer. 1997. “Social Ostracism by Coworkers: Does Rejection Lead to Loafing or Compensation?” *Personality and Social Psychology Bulletin* 23 (7): 693–706. https://doi.org/10.1177/0146167297237003.

Wilson, Nicole L., and Camilla M. Holmvall. 2013. “The Development and Validation of the Incivility from Customers Scale.” *Journal of Occupational Health Psychology* 18 (3): 310–26. https://doi.org/10.1037/a0032753.

Wolf, Lisa A., Altair M. Delao, and Cydne Perhats. 2014. “Nothing Changes, Nobody Cares: Understanding the Experience of Emergency Nurses Physically or Verbally Assaulted While Providing Care.” *Journal of Emergency Nursing* 40 (4): 305–10. https://doi.org/10.1016/j.jen.2013.11.006.

Wu, Ivan H. C., Brent Lyons, and Frederick T. L. Leong. 2015. “How Racial/Ethnic Bullying Affects Rejection Sensitivity: The Role of Social Dominance Orientation.” *Cultural Diversity and Ethnic Minority Psychology* 21 (1): 156–61. https://doi.org/10.1037/a0037930.

Yu, Lingtao, and Michelle K. Duffy. 2015. “The Spiral of Abuse? Reciprocal Relationships among Abusive Supervision, Performance, and Motives.” *Academy of Management Proceedings* 2015 (1): 15436. https://doi.org/10.5465/ambpp.2015.167.

Yuan, Zhenyu, YoungAh Park, and Michael T. Sliter. 2020. “Put You down versus Tune You out: Further Understanding Active and Passive e-Mail Incivility.” *Journal of Occupational Health Psychology* 25 (5): 330–44. https://doi.org/10.1037/ocp0000215.

Zadro, Lisa, Kipling D Williams, and Rick Richardson. 2004. “How Low Can You Go? Ostracism by a Computer Is Sufficient to Lower Self-Reported Levels of Belonging, Control, Self-Esteem, and Meaningful Existence.” *Journal of Experimental Social Psychology* 40 (4): 560–67. https://doi.org/10.1016/j.jesp.2003.11.006.

Zahlquist, Lena, Jørn Hetland, Anders Skogstad, Arnold B. Bakker, and Ståle Valvatne Einarsen. 2019. “Job Demands as Risk Factors of Exposure to Bullying at Work: The Moderating Role of Team-Level Conflict Management Climate.” *Frontiers in Psychology* 10 (September): 1–11. https://doi.org/10.3389/fpsyg.2019.02017.

Zapf, Dieter, and Claudia Gross. 2001. “Conflict Escalation and Coping with Workplace Bullying: A Replication and Extension.” *European Journal of Work and Organizational Psychology* 10 (4): 497–522. https://doi.org/10.1111/j.1600-0587.1996.tb01264.x.

Zapf, Dieter, Carmen Knorz, and Matthias Kulla. 1996. “On the Relationship between Mobbing Factors, and Job Content, Social Work Environment, and Health Outcomes.” *European Journal of Work and Organizational Psychology* 5 (2): 215–37. https://doi.org/10.1080/13594329608414856.

Zhou, Jing, and Gerald R. Ferris. 1995. “The Dimensions and Consequences of Organizational Politics Perceptions: A Confirmatory Analysis1.” *Journal of Applied Social Psychology* 25 (19): 1747–64. https://doi.org/10.1111/j.1559-1816.1995.tb01816.x.

Žukauskas, Pranas, and Jolita Vveinhardt. 2013. “Mobbing and Bullying within the Organization: Socio-Demographic Portrait of the Victim.” *Krytyka ﻿Prawa* 5: 693–716. https://doi.org/10.7206/kp.2080-1084.35.
