# Supplementary material for: A Systematic Review of Negative Work Behavior: Toward an Integrated Definition
Source: Front Psychol. 2021 Oct 27;12:726973. doi: 10.3389/fpsyg.2021.726973 (PMC8578924; doi:10.3389/fpsyg.2021.726973)
Supplement: Supplementary file 2 [file Table_2.docx]

**Supplementary**

**TABLE 2 |** B. Nature of harm in NWB labels derived from study titles

| **Construct NWB** | **Nature of Harm: B1. physical B2. material B3. psychological B4. social** |
| --- | --- |
| Aggression B  B  B  B | 1. physical damage (Fredericksen and McCorkle 2013), somatic symptoms, headaches, disturbances in sleep patterns, increased probability of bringing a weapon into one's work environment (Haines, Marchand, and Harvey 2006; Marchand, Demers, and Durand 2005; McDermut, Haaga, and Kirk 2000), high blood pressure, coronary heart disease and suicide (Kivimäki et al. 2005; Lutgen-Sandvik, Tracy, and Alberts 2007; Namie 2003) 2. economic harm﻿ (Fredericksen and McCorkle 2013) 3. psychological harm (Schat and Kelloway 2005), negative impact on functioning effectively (Keashly and Harvey 2005),﻿ reduced job satisfaction, psychological distress, feelings of fear (Budd, Arvey, and Lawless 1996; Haines, Marchand, and Harvey 2006; Marchand, Demers, and Durand 2005; McDermut, Haaga, and Kirk 2000; Rogers and Kelloway 1997; Schat and Kelloway 2003), depression and posttraumatic stress disorder (Kivimäki et al. 2005; Leymann and Gustafsson 1996; Lutgen-Sandvik, Tracy, and Alberts 2007; Namie 2003; Van den Bossche et al. 2012), respond with enacted aggression (Hershcovis et al. 2012; Lian et al. 2014; Detert et al. 2007; Mayer et al. 2012), respons is revenge (Aquino, Tripp, and Bies 2006) 4. socioeconomic and moral harm﻿ (Fredericksen and McCorkle 2013) |
| Bullying B  B  B  B | 1. psychosomatic illness (Djurkovic, McCormack, and Casimir 2004), musculoskeletal disorders (Choi, Yi, and Kim 2018), sleep dysfunction (Morten Birkeland Nielsen et al. 2018), ﻿somatization, physical health problems (Nielsen & Einarsen, 2012), chronic neck pain (Kääriä et al. 2012), diabetes type 2 (Xu et al. 2018) 2. employer monetary costs (Sabbath et al. 2018), socioeconomic impact (Reknes et al. 2019), absenteeism, turnover and productivity (Giga, Hoel, and Lewis 2008),﻿ sickness absence (Nielsen, Indregard, & Overland, 2016), disability retirement as consequence (Nielsen, Emberland, and Knardahl, 2017) 3. hurtful effect on target (Pallesen et al. 2017), mental health problems, anxiety, depression, post-traumatic stress, general strain, burnout (Nielsen and Einarsen, 2012), cyberbullying in social network as reaction (Escartín et al. 2019) 4. damages the victim’s friendship networks (indirect bullying; Björkqvist, Österman, and Lagerspetz 1994), health and family consequences, affecting health of victims children (Ng 2019)﻿ |
| Mobbing B  B  B  B | 1. considerable psychosomatic misery (Leymann and Tallgren 1989), sleep problems (Hansen et al. 2014), risk of cardiovascular disease (Hansen, Hogh, and Persson 2011), fatigue (Reknes et al. 2014) 2. material damage (Wolmerath 2019), claims and legal fees ﻿(Bultena and Whatcott 2008), ﻿long periods of sick leave; a catastrophic drop in production by the whole group; the necessity for frequent intervention by personnel officers, personnel consultants, managers of various grades, occupational health staff, external consultants, the company's health care centers (Leymann 1990) 3. substance abuse (Dobry, Braquehais, and Sher 2013), more prone to psychiatric and neurological diseases (Jacob and Kostev 2017), loss of dignity, lowered self-confidence and productivity, and an excessive amount of non-work-related stress and other related health issues (Kircher et al. 2011), mental health problems (Verkuil, Atasayi, and Molendijk 2015), post-traumatic stress disorder (Baran Tatar and Yuksel 2018), burnout (Trépanier, Fernet, and Austin 2015) 4. social misery (Leymann and Tallgren 1989), affecting the family of the victim and intimate relationships (Sperry and Duffy 2009) |
| Harassment B  / Discrimination B  B  B | 1. health difficulties (Hyde et al. 2006; Spector, Chen, and O’Connell 2000), problem drinking (Rospenda, Richman, and Shannon 2009), headache (Tynes, Johannessen, and Sterud 2013) 2. discomfort, loss of income and employment (Lutgen-Sandvik 2006; Zapf and Gross 2001), for organizations: absenteeism (Ayoko, Callan, and Härtel 2003; Giebels and Janssen 2005), lowered productivity (Chen, Tjosvold, & Su Fang, 2005), causes everyday NWB through digital technologies (Henry and Powell 2018) 3. psychological distress (Ayoko, Callan, and Härtel, 2003; Giebels and Janssen, 2005; Hauge, Skogstad, and Einarsen, 2010), causes toxic work environments in organizations (Jonason, Slomski, and Partyka 2012) 4. affects the dignity of workers, effects for society (Di Martino 2009), depressive symptoms in family members (Crouter et al. 2006) |
| Deviance B  B  B  B | 1. no physical well-being (Robinson, Wang, & Kiewitz, 2014), bodily harm (Bennett and Robinson, 2000a; Rioux and Marie-Élène Roberge, 2005; Robinson and Bennett, 1995) 2. costs in the employee-customer relationship, functional quality, and company performance (Harris and Ogbonna, 2006; Hollinger and Adams, 2010), ﻿﻿disruption of procedures, productivity, and profitability (Aubé, Rousseau, Mama, and Morin, 2009; Dalal, 2005; Lanyon and Goodstein, 2003; Pearson, Andersson, and Porath, 2005; Robinson, 2008; Spector and Fox, 2005; Vardi and Weitz, 2004), decreased productivity, increased turnover (O’Leary-Kelly, Griffin, and Glew, 1996), extremely costly both to organizations and to society (Bennett and Robinson 2003) 3. harms well-being of target and organization members (Robinson and Bennett, 1995),﻿ uncertainty, negative reactions (Tangirala and Alge 2006; Thau et al. 2009), humiliate, depreciate, reduce, destroy self-worth (Bennett and Robinson, 2000; Rioux and Marie-Élène Roberge, 2005; Robinson and Bennett, 1995), lower self-esteem, team spirit (Harris and Ogbonna, 2006)﻿, low morale, and stress (O’Leary-Kelly, Griffin, and Glew 1996) 4. work-family conflict (Darrat, Amyx, and Bennett 2010) |
| Counterproductive Work B Behavior (CWB) B  B  B | 1. somatic pressure, exhaustion and burnout (Cortina and Magley 2003; Cropanzano et al. 1997) 2. defaced or destroyed property belongings (Chen and Spector, 1992), severe effect on quality of products, technological innovation of enterprises, leak of core secrets, fatal harm to enterprises (Chen, 2017) 3. decreased job satisfaction, altruism, organizational commitment, and conscientiousness, and increased intention to leave and deviant behavior (Peng 2011) 4. withdraw behavior, ﻿absenteeism (Yperen, Hagedoorn, and Geurts 1996)   ﻿ |
| Violence B  B  B  B | 1. death, growth disorders (Campo and Klijn 2018), ﻿physical pain (Ray 2007; Ridenour et al. 2015) 2. decreased commitment to the profession and organization, lower quality of teamwork, increased absenteeism and lower quality of work (Camerino et al. 2008; Esmaeilpour, Salsali, and Ahmadi 2011) 3. psychological damage, deprivation (Campo and Klijn 2018), psychological and emotional problems (Ray 2007), depression, anxiety and work-related stress (Aytaç and Dursun 2012; Rodwell and Demir 2012), emotional exhaustion and burnout (Budin et al. 2013; Waschgler et al. 2013) 4. sexual, racial problems (Ray 2007), lower satisfaction of life (Budin et al. 2013; Waschgler et al. 2013) |
| Abuse B  /Abusive supervision  B  B      B | 1. alcohol and other drug use (Richman et al. 1999), physical symptoms and illness (Bambi et al. 2018), violates workers’ physical integrity (Richman et al. 1999) 2. causes direct reactions of workplace bullying with costs for individual and organization (Einarsen et al., 2003), turnover (Bambi et al. 2018), causes health care costs, lost productivity (Tepper et al. 2006) 3. short- and long-term effects on lowering the victim's self-perceptions and well-being (Keashly and Harvey 2005), results in symptomatic distress (Richman et al. 1999), has psychological and behavioral impact (Bambi et al. 2018), ﻿causes moral outrage, anger, retaliation, resistance (Mitchell, Vogel, and Folger 2015; Priesemuth and Schminke 2019), violates workers’ psychological and/or professional integrity (Richman et al. 1999) 4. results in ﻿interpersonal counterproductive work behaviors (Tepper 2007), causes ﻿family undermining as displaced aggression (Hoobler and Brass 2006) |
| Terror B  B  B  B | 1. lower levels of health, health problems (Giorgi et al. 2015) 2. harms the victim’s communication channels, information flows, reputation and/or professionalism (Leymann 1996) 3. stress and anxiety (Leymann 1996), undermines or sabotages the motivation, well-being, job satisfaction (Einarsen, Aasland, and Skogstad, 2007), ﻿turns into a relational hell of human existence (Zamperini and Menegatto 2013) 4. being defenseless and unable to get any help (Leymann 1996), causes differences in victimization between people with equal power and people with power differences (Leymann and Zapf 1990), detrimental role stress consequences (Örtqvist and Wincent 2006), involving behavior such as harassment and mistreatment (Einarsen, Aasland, and Skogstad, 2007) |
| Injustice B  B  B  B | 1. results in physical harm (Citron and Franks, 2014) 2. loss of income and employment (Lutgen-Sandvik 2006; Zapf and Gross 2001), financial risk for personal safety, reputational damage (Citron and Franks 2014) 3. affects the dignity of workers (Di Martino 2009), causes a psychological, risk for personal safety, causes shame (Citron and Franks 2014), causes different CWBs with mental problem effects (Martinko, Gundlach, and Douglas 2002) 4. discomfort in another person (Lutgen-Sandvik 2006; Zapf and Gross 2001), effects for society (Di Martino 2009), social risk for personal safety (Citron and Franks 2014), paranoia at home from digital injustice (Citron and Franks 2014) |
| Interpersonal conflict B  B  B  B | 1. cardiovascular disease (Jacob and Kostev 2017) 2. reduced performance, absenteeism, and turnover (Sliter et al. 2011) 3. reduced job satisfaction and organizational commitment (Frone 2000), negative employee outcomes, such as negative emotions (Fox, Spector, and Miles,2001), anger behaviors﻿ (Sliter, Pui, Sliter, and Jex, 2011) 4. reduced life satisfaction (Appelberg et al. 1991) |
| Victimization B  /Scapegoating B  B  B | 1. alcohol use problems (McFarlin et al. 2001), physical harm (Aquino and Thau 2009) 2. reduced job performance (Jensen, Patel, and Raver 2014), high costs for ﻿sickness absenteeism, job turnover, exclusion from social work- related processes, unemployment (Glambek, Skogstad, and Einarsen 2016; Morten Birkeland Nielsen and Einarsen 2018; Theorell et al. 2015) 3. mental health problems (Verkuil, Atasayi, and Molendijk 2015), decreased well-being, job satisfaction (Glambek, Skogstad, and Einarsen 2016; Morten Birkeland Nielsen and Einarsen 2018; Theorell et al. 2015), negative psychological outcomes (Hershcovis and Barling 2010) 4. losing the respect of others, loss of standing in the group, or damage to one’s reputation (Wright et al. 2004), |
| Micropolitics B B  B  B | 1. increased levels of absenteeism (Vigoda 2001) 2. turnover intentions (Valle and Perrewe 2000), ﻿denial of the desired benefit (Ferris et al., 2000b), reduced organizational efficiency and effectiveness (Kacmar et al. 1999; Mintzberg 1983) 3. lower job satisfaction (Vigoda 2001), variety of dysfunctional attitudinal, psychological health, and behavioral outcomes, increased stress, burnout, turnover intentions and counterproductive work behavior, and decreased citizenship behavior and job performance (Bedi and Schat 2013; Vigoda-Gadot and Kapun 2005; Atinc, Fuller, and Darrat 2010) 4. higher intentions of exit and neglect and lower levels of loyalty (Vigoda 2001), censure by the political target and co-workers, and diminution of reputation and perceived trustworthiness (Ferris et al., 2000b) |
| Ostracism B  B  B  B | 1. physical pain (Eisenberger, Lieberman, and Williams 2003), health problems (O’Reilly et al. 2015) 2. reduced organizational engagement (Wu et al. 2016), no personal or organizational benefit (Balliet and Ferris 2013) 3. negative mood (Gonsalkorale and Williams 2007), psychologically aversive reactions, including anger (Chow, Tiedens, and Govan 2008), anxiety, job search behavior, negative attitudes toward work, low psychological health, lower job satisfaction, higher turnover intention (Ferris, Brown, Berry, and Lian, 2008; Hitlan and Noel, 2009), reduced personal well-being, emotional exhaustion and psychological distress (Ferris et al., 2008; Wu, Yim, Kwan, and Zhang, 2012), depression, worthlessness, alienation and helplessness (Riva et al. 2017), instigates stress (Sarfraz et al. 2019), causes individual and/or organizational CWB (Zhao, Peng, and Sheard 2013), lower self-esteem (Ferris, Lian, Brown, and Morrison, 2015) 4. solitude seeking, ﻿stronger desires to be alone (Ren, Wesselmann, and Williams 2016) |
| Incivility B  B  B  B | 1. physical health problems (Bunk and Magley, 2013; Lim and Cortina, 2005), health problems among targets and witnesses (Lim et al., 2008; Pearson et al., 2005; Pearson and Porath, 2009), adiposity (Sliter, Sliter, Withrow, and Jex, 2012) 2. negative occupational and organizational outcomes (Bunk and Magley, 2013; Lim and Cortina, 2005), ﻿cost millions, decrease in performance and attendance (Lim et al., 2008; Pearson et al., 2005; Pearson and Porath, 2009), monetary costs employer (Sabbath et al. 2018)(Porath, 2015; Porath & Pearson, 2013; Yeung & Griffin, 2008), lower costumer service quality (Sliter, Jex, Wolford, and McInnerney, 2010), higher health care utilization (Sabbath et al. 2018) 3. psychological problems, ﻿job dissatisfaction, interpersonal conflict, turnover intent (Hershcovis 2011), burnout, withdrawal (Sliter et al. 2012), lower self-efficasy, negative mood, higher interpersonal problems such as privacy invasion, ﻿exclusionary behavior, turnover intention (Riadi, Hendryadi, and Tricahyadinata 2019) 4. a double-edged sword, bringing harm to target and damage to status perpetrator (Williams 2001), lower marital satisfaction and higher family-to-work conflict (Ferguson 2012) |
| Social safety B  B  B  B | 1. work-related injury and illness, lost time injury (disabilities, short and long absence), economic cost for worker, organization, society (O’Neill, Martinov-Bennie, and Cheung 2013), fatality, injury, or illness (Health and safety department US 2016), ﻿work-related ill-health, sleeping problems (Aumayr-Pintar, Cerf, and Surdykowska 2019; Eurofound 2013), ﻿lost life years (Kreis and Bödeker 2004) 2. social insurance on statutory sick pay, disability allowances, industrial injuries disablement and incapacity benefits (Adema and Ladaique 2009), economic losses (ILO, 2003), sickness absence (Lusinyan and Bonato 2007), private insurance and health care outlays of affected individuals, the indirect costs of companies (e.g. training inexperienced replacement workers, administrative expenses, production bottlenecks, low employee morale; Pouliakas and Theodossiou 2013), socio-economic costs of (sickness) absence in GDP (Eurofound 2013) 3. ﻿reduced psychological well-being,﻿ depression, stress, fatigue (Eurofound 2013) ﻿ 4. ﻿the impact on families and communities and the inefficiency of having a large proportion of a potentially active workforce disabled, idle or prematurely retired (Pouliakas and Theodossiou 2013) |

# References Table 4 |

Adema, W., and M Ladaique. 2009. “How Expensive Is the Welfare State?: Gross and Net Indicators in the OECD Social Expenditure Database (SOCX).” 92. OECD Social, Employment and Migration Working Papers. Paria. https://doi.org/https://doi.org/10.1787/220615515052.

Appelberg, Kirsi, Kalle Romanov, Marja-Liisa Honkasalo, and Markku Koskenvuo. 1991. “Interpersonal Conflicts at Work and Psychosocial Characteristics of Employees.” *Social Science & Medicine* 32 (9): 1051–56. https://doi.org/10.1016/0277-9536(91)90162-6.

Aquino, Karl, and Stefan Thau. 2009. “Workplace Victimization: Aggression from the Target’s Perspective.” *Annual Review of Psychology* 60 (1): 717–41. https://doi.org/10.1146/annurev.psych.60.110707.163703.

Aquino, Karl, Thomas M. Tripp, and Robert J. Bies. 2006. “Getting Even or Moving on? Power, Procedural Justice, and Types of Offense as Predictors of Revenge, Forgiveness, Reconciliation, and Avoidance in Organizations.” *Journal of Applied Psychology* 91 (3): 653–68. https://doi.org/10.1037/0021-9010.91.3.653.

Atinc, Guclu, Jerry Bryan Fuller, and Mahmoud A Darrat. 2010. “Perceptions of Organizational Politics: A Meta-Analysis of Theoretical Antecedents.” *Journal of Managerial Issues* XXII (4): 494–513. https://doi.org/10.2307/25822527.

Aubé, C., V. Rousseau, C. Mama, and E. M. Morin. 2009. “Counterproductive Behaviors and Psychological Well-Being: The Moderating Effect of Task Interdependence.” *Journal of Business and Psychology* 24 (3): 351–61. https://doi.org/10.1007/s10869-009-9113-5.

Aumayr-Pintar, Christine, Catherine Cerf, and Barbara Surdykowska. 2019. “Annual Review of Working Life 2018.” Luxembourg. http://eurofound.link/ef19029.

Ayoko, Oluremi B., Victor J. Callan, and Charmine E.J. Härtel. 2003. “Workplace Conflict, Bullying, and Counterproductive Behaviors.” *The International Journal of Organizational Analysis* 11 (4): 283–301. https://doi.org/10.1108/eb028976.

Aytaç, Serpil, and Salih Dursun. 2012. “The Effect on Employees of Violence Climate in the Workplace.” *Work* 41: 3026–31. https://doi.org/10.3233/WOR-2012-0559-3026.

Balliet, Daniel, and D.Lance Ferris. 2013. “Ostracism and Prosocial Behavior: A Social Dilemma Perspective.” *Organizational Behavior and Human Decision Processes* 120 (2): 298–308. https://doi.org/10.1016/j.obhdp.2012.04.004.

Bambi, Stefano, Chiara Foà, Christian De Felippis, Alberto Lucchini, Andrea Guazzini, and Laura Rasero. 2018. “Workplace Incivility, Lateral Violence and Bullying among Nurses. A Review about Their Prevalence and Related Factors.” *Acta Biomedica* 89 (6): 51–79. https://doi.org/10.23750/abm.v89i6-S.7461.

Baran Tatar, Zeynep, and Sahika Yuksel. 2018. “Mobbing at Workplace - Psychological Trauma and Documentation of Psychiatric Symptoms.” *Archives of Neuropsychiatry* 56 (1): 57–62. https://doi.org/10.29399/npa.22924.

Bedi, Akanksha, and Aaron C. H. Schat. 2013. “Perceptions of Organizational Politics: A Meta-Analysis of Its Attitudinal, Health, and Behavioural Consequences.” *Canadian Psychology/Psychologie Canadienne* 54 (4): 246–59. https://doi.org/10.1037/a0034549.

Bennett, Rebecca J., and Sandra L. Robinson. 2000. “Development of a Measure of Workplace Deviance.” *Journal of Applied Psychology* 85 (3): 349–60. https://doi.org/10.1037/0021-9010.85.3.349.

———. 2003. “The Past, Present, and Future of Workplace Deviance Research.” In *Organizational Behavior: The State of the Science*, edited by Jerald Greenberg, 247–81. Mahwah, NJ: Lawrence Erlbaum Associates Publishers. https://psycnet.apa.org/record/2003-02890-007.

Björkqvist, Kaj, Karin Österman, and Kirsti M. J. Lagerspetz. 1994. “Sex Differences in Covert Aggression among Adults.” *Aggressive Behavior* 20 (1): 27–33. https://doi.org/10.1002/1098-2337(1994)20:1<27::AID-AB2480200105>3.0.CO;2-Q.

Bossche, S Van den, M Van der Klauw, J F Ybema, E de Vroome, and A Venema. 2012. “Agressie Op Het Werk. Ontwikkelingen, Risico’s, Impact En Behoefte Aan Maatregelen (Aggression at Work. Developments, Risks, Impact and Need for Measures).” Hoofddorp.

Budd, John W., Richard D. Arvey, and Peggy Lawless. 1996. “Correlates and Consequences of Workplace Violence.” *Journal of Occupational Health Psychology* 1 (2): 197–210. https://doi.org/10.1037/1076-8998.1.2.197.

Budin, Wendy C., Carol S. Brewer, Ying-Yu Chao, and Christine Kovner. 2013. “Verbal Abuse From Nurse Colleagues and Work Environment of Early Career Registered Nurses.” *Journal of Nursing Scholarship* 45 (3): 308–16. https://doi.org/10.1111/jnu.12033.

Bultena, Charles D, and Richard B. Whatcott. 2008. “Bush Whacked at Work: A Comparative Analysis of Mobbing & Bullying at Work.” *Proceedings of ASBBS* 15 (1): 652–66.

Bunk, Jennifer A., and Vicki J. Magley. 2013. “The Role of Appraisals and Emotions in Understanding Experiences of Workplace Incivility.” *Journal of Occupational Health Psychology* 18 (1): 87–105. https://doi.org/10.1037/a0030987.

Camerino, Donatella, Madeleine Estryn-Behar, Paul Maurice Conway, Beatrice Isabella Johanna Maria van Der Heijden, and Hans-Martin Hasselhorn. 2008. “Work-Related Factors and Violence among Nursing Staff in the European NEXT Study: A Longitudinal Cohort Study.” *International Journal of Nursing Studies* 45 (1): 35–50. https://doi.org/10.1016/j.ijnurstu.2007.01.013.

Campo, Varinia Rodríguez, and Tatiana Paravic Klijn. 2018. “Verbal Abuse and Mobbing in Pre-Hospital Care Services in Chile.” *Revista Latino-Americana de Enfermagem* 25 (January): e2956. https://doi.org/10.1590/1518-8345.2073.2956.

Chen, Peter Y., and Paul E. Spector. 1992. “Relationships of Work Stressors with Aggression, Withdrawal, Theft and Substance Use: An Exploratory Study.” *Journal of Occupational and Organizational Psychology* 65 (3): 177–84. https://doi.org/10.1111/j.2044-8325.1992.tb00495.x.

Chen, Si-hua. 2017. “An Evolutionary Game Model of Knowledge Workers’ Counterproductive Work Behaviors Based on Preferences.” *Complexity*, 1–11. https://doi.org/10.1155/2017/3295436.

Chen, Yifeng, Dean Tjosvold, and Sofia Su Fang. 2005. “Working with Foreign Managers: Conflict Management for Effective Leader Realtionships in China.” *International Journal of Conflict Management* 16 (3): 265–86. https://doi.org/10.1108/eb022932.

Choi, Sookja, Yunjeong Yi, and Jiyun Kim. 2018. “Exposure to Adverse Social Behavior in the Workplace and Sickness Presenteeism among Korean Workers: The Mediating Effects of Musculoskeletal Disorders.” *International Journal of Environmental Research and Public Health* 15 (10): 219. https://doi.org/10.3390/ijerph15102198.

Chow, Rosalind M., Larissa Z. Tiedens, and Cassandra L. Govan. 2008. “Excluded Emotions: The Role of Anger in Antisocial Responses to Ostracism.” *Journal of Experimental Social Psychology* 44 (3): 896–903. https://doi.org/10.1016/j.jesp.2007.09.004.

Citron, Danielle Keats, and Mary Anne Franks. 2014. “Criminalizing Revenge Porn.” *Wake Forest Law Review* 49 (2014–1): 345. https://scholarship.law.bu.edu/faculty_scholarship/643.

Cortina, Lilia M., and Vicki J. Magley. 2003. “Raising Voice, Risking Retaliation: Events Following Interpersonal Mistreatment in the Workplace.” *Journal of Occupational Health Psychology* 8 (4): 247–65. https://doi.org/10.1037/1076-8998.8.4.247.

Cropanzano, Russell, John C Howes, Alicia A Grandey, and Paul Toth. 1997. “The Relationship of Organizational Politics and Support to Work Behaviors, Attitudes, and Stress.” *Journal of Organizational Behavior* 18 (2): 159–80. https://doi.org/10.1002/(SICI)1099-1379(199703)18:2<159::AID-JOB795>3.0.CO;2-D.

Crouter, Ann C., Kelly D. Davis, Kimberly Updegraff, Melissa Delgado, and Melissa Fortner. 2006. “Mexican American Fathers’ Occupational Conditions: Links to Family Members’ Psychological Adjustment.” *Journal of Marriage and Family* 68 (4): 843–58. https://doi.org/10.1111/j.1741-3737.2006.00299.x.

Dalal, Reeshad S. 2005. “A Meta-Analysis of the Relationship between Organizational Citizenship Behavior and Counterproductive Work Behavior.” *Journal of Applied Psychology* 90 (6): 1241–55. https://doi.org/10.1037/0021-9010.90.6.1241.

Darrat, Mahmoud, Douglas Amyx, and Rebecca Bennett. 2010. “An Investigation into the Effects of Work–Family Conflict and Job Satisfaction on Salesperson Deviance.” *Journal of Personal Selling & Sales Management* 30 (3): 239–51. https://doi.org/10.2753/PSS0885-3134300304.

Detert, James R., Linda K. Treviño, Ethan R. Burris, and Meena Andiappan. 2007. “Managerial Modes of Influence and Counterproductivity in Organizations: A Longitudinal Business-Unit-Level Investigation.” *Journal of Applied Psychology* 92 (4): 993–1005. https://doi.org/10.1037/0021-9010.92.4.993.

Djurkovic, Nikola, Darcy McCormack, and Gian Casimir. 2004. “The Physical and Psychological Effects of Workplace Bullying and Their Relationship to Intention to Leave: A Test of the Psychosomatic and Disability Hypotheses.” *International Journal of Organization Theory & Behavior* 7 (4): 469–97. https://doi.org/10.1108/IJOTB-07-04-2004-B001.

Dobry, Yuriy, María Dolores Braquehais, and Leo Sher. 2013. “Bullying, Psychiatric Pathology and Suicidal Behavior.” *International Journal of Adolescent Medicine and Health* 25 (3): 295–99. https://doi.org/10.1515/ijamh-2013-0065.

Einarsen, S, Merethe Schanke Aasland, and Anders Skogstad. 2007. “Destructive Leadership Behaviour: A Definition and Conceptual Model.” *The Leadership Quarterly* 18 (3): 207–16. https://doi.org/10.1016/j.leaqua.2007.03.002.

Einarsen, S, Helge Hoel, Dieter & Zapf, and Cary Lynn Cooper. 2003. *Bullying and Emotional Abuse in the Workplace : International Perspectives in Research and Practice*. Edited by Ståle Einarsen, Helge Hoel, Dieter Zapf, and Cary L. Cooper. 1st ed. London ; New York: Taylor & Francis. https://books.google.es/books.

Eisenberger, Naomi I., Matthew D Lieberman, and kipling D Williams. 2003. “Does Rejection Hurt? An FMRI Study of Social Exclusion.” *Science* 302 (5643): 290–92. https://doi.org/10.1126/science.1089134.

Escartín, Jordi, Ivana Vranjes, Elfi Baillien, and Guy Notelaers. 2019. “Workplace Bullying and Cyberbullying Scales: An Overview.” In *Concepts, Approaches and Methods, Handbooks of Workplace Bullying, Emotional Abuse and Harassment*, edited by Premilla D’Cruz, 1–44. Singapore. https://doi.org/10.1007/978-981-10-5334-4_10-1.

Esmaeilpour, M., M. Salsali, and F. Ahmadi. 2011. “Workplace Violence against Iranian Nurses Working in Emergency Departments.” *International Nursing Review* 58 (1): 130–37. https://doi.org/10.1111/j.1466-7657.2010.00834.x.

Eurofound. 2013. “Physical and Psychological Violence at the Workplace.” Luxembourg. https://doi.org/10.2806/49169.

Ferguson, Merideth. 2012. “You Cannot Leave It at the Office: Spillover and Crossover of Coworker Incivility.” *Journal of Organizational Behavior* 33 (4): 571–88. https://doi.org/10.1002/job.774.

Ferris, D. Lance, Douglas J. Brown, Joseph W. Berry, and Huiwen Lian. 2008. “The Development and Validation of the Workplace Ostracism Scale.” *Journal of Applied Psychology* 93 (6): 1348–66. https://doi.org/10.1037/a0012743.

Ferris, D. Lance, Huiwen Lian, Douglas J. Brown, and Rachel Morrison. 2015. “Ostracism, Self-Esteem, and Job Performance: When Do We Self-Verify and When Do We Self-Enhance?” *Academy of Management Journal* 58 (1): 279–97. https://doi.org/10.5465/amj.2011.0347.

Ferris, Gerald R, Gloria Harrell-Cook, and James H Dulebohn. 2000. “Organizational Politics: The Nature of the Relationship between Politics Perceptions and Political Behavior.” *Research in the Sociology of Organizations* 17: 89–130. https://doi.org/10.1016/S0733-558X(00)17004-1.

Fox, Suzy, Paul E. Spector, and Don Miles. 2001. “Counterproductive Work Behavior (CWB) in Response to Job Stressors and Organizational Justice: Some Mediator and Moderator Tests for Autonomy and Emotions.” *Journal of Vocational Behavior* 59 (3): 291–309. https://doi.org/10.1006/jvbe.2001.1803.

Fredericksen, Elizabeth D., and Suzanne McCorkle. 2013. “Explaining Organizational Responses to Workplace Aggression.” *Public Personnel Management* 42 (2): 223–38. https://doi.org/10.1177/0091026013487050.

Frone, Michael R. 2000. “Interpersonal Conflict at Work and Psychological Outcomes: Testing a Model among Young Workers.” *Journal of Occupational Health Psychology* 5 (2): 246–55. https://doi.org/10.1037/1076-8998.5.2.246.

Giebels, Ellen, and Onne Janssen. 2005. “Conflict Stress and Reduced Well-Being at Work: The Buffering Effect of Third-Party Help.” *European Journal of Work and Organizational Psychology* 14 (2): 137–55. https://doi.org/10.1080/13594320444000236.

Giga, Sabir I., Helge Hoel, and Duncan Lewis. 2008. “The Costs of Workplace Bullying.” www.researchgate.net/profile/Sabir_Giga/publication/260246863_The_Costs_of_Workplace_Bullying.

Giorgi, Gabriele, Serena Mancuso, Francisco Javier Fiz Perez, Francesco Montani, Francois Courcy, and Giulio Arcangeli. 2015. “Does Leaders’ Health (and Work-Related Experiences) Affect Their Evaluation of Followers’ Stress?” *Safety and Health at Work* 6 (3): 249–55. https://doi.org/10.1016/j.shaw.2015.07.005.

Glambek, Mats, Anders Skogstad, and Ståle Einarsen. 2016. “Do the Bullies Survive? A Five-Year, Three-Wave Prospective Study of Indicators of Expulsion in Working Life among Perpetrators of Workplace Bullying.” *Industrial Health* 54 (1): 68–73. https://doi.org/10.2486/indhealth.2015-0075.

Gonsalkorale, Karen, and Kipling D. Williams. 2007. “The KKK Won’t Let Me Play: Ostracism Even by a Despised Outgroup Hurts.” *European Journal of Social Psychology* 37 (6): 1176–86. https://doi.org/10.1002/ejsp.392.

Haines, Victor Y., Alain Marchand, and Steve Harvey. 2006. “Crossover of Workplace Aggression Experiences in Dual-Earner Couples.” *Journal of Occupational Health Psychology* 11 (4): 305–14. https://doi.org/10.1037/1076-8998.11.4.305.

Hansen, Åse Marie, Annie Hogh, Anne Helene Garde, and Roger Persson. 2014. “Workplace Bullying and Sleep Difficulties: A 2-Year Follow-up Study.” *International Archives of Occupational and Environmental Health* 87 (3): 285–94. https://doi.org/10.1007/s00420-013-0860-2.

Hansen, Åse Marie, Annie Hogh, and Roger Persson. 2011. “Frequency of Bullying at Work, Physiological Response, and Mental Health.” *Journal of Psychosomatic Research* 70 (1): 19–27. https://doi.org/10.1016/j.jpsychores.2010.05.010.

Harris, L. C., and Emmanuel Ogbonna. 2006. “Service Sabotage: A Study of Antecedents and Consequences.” *Journal of the Academy of Marketing Science* 34 (4): 543–58. https://doi.org/10.1177/0092070306287324.

Hauge, Lars Johan, Anders Skogstad, and Ståle Einarsen. 2010. “The Relative Impact of Workplace Bullying as a Social Stressor at Work.” *Scandinavian Journal of Psychology* 51 (5): 426–33. https://doi.org/10.1111/j.1467-9450.2010.00813.x.

Health and safety department US. 2016. “Department of Labor: OSHA 3148-06R 2016.” *OSHA Report*. https://www.osha.gov/laws-regs/federalregister/2016-12-07.

Henry, Nicola, and Anastasia Powell. 2018. “Technology-Facilitated Sexual Violence: A Literature Review of Empirical Research.” *Trauma, Violence, & Abuse* 19 (2): 195–208. https://doi.org/10.1177/1524838016650189.

Hershcovis, M. Sandy. 2011. “‘Incivility, Social Undermining, Bullying…oh My!’: A Call to Reconcile Constructs within Workplace Aggression Research.” *Journal of Organizational Behavior* 32 (3): 499–519. https://doi.org/10.1002/job.689.

Hershcovis, M. Sandy, and Julian Barling. 2010. “Comparing Victim Attributions and Outcomes for Workplace Aggression and Sexual Harassment.” *Journal of Applied Psychology* 95 (5): 874–88. https://doi.org/10.1037/a0020070.

Hershcovis, M. Sandy, Tara C. Reich, Sharon K. Parker, and Jennifer Bozeman. 2012. “The Relationship between Workplace Aggression and Target Deviant Behaviour: The Moderating Roles of Power and Task Interdependence.” *Work & Stress* 26 (1): 1–20. https://doi.org/10.1080/02678373.2012.660770.

Hitlan, Robert T., and Jennifer Noel. 2009. “The Influence of Workplace Exclusion and Personality on Counterproductive Work Behaviours: An Interactionist Perspective.” *European Journal of Work and Organizational Psychology* 18 (4): 477–502. https://doi.org/10.1080/13594320903025028.

Hollinger, R. C, and A Adams. 2010. “National Retail Security Survey Final Report.” Gainsville, FL.

Hoobler, Jenny M., and Daniel J. Brass. 2006. “Abusive Supervision and Family Undermining as Displaced Aggression.” *Journal of Applied Psychology* 91 (5): 1125–33. https://doi.org/10.1037/0021-9010.91.5.1125.

Hyde, Martin, Paavo Jappinen, Tores Theorell, and Gabriel Oxenstierna. 2006. “Workplace Conflict Resolution and the Health of Employees in the Swedish and Finnish Units of an Industrial Company.” *Social Science & Medicine* 63 (8): 2218–27. https://doi.org/10.1016/j.socscimed.2006.05.002.

Jacob, Louis, and Karel Kostev. 2017. “Conflicts at Work Are Associated with a Higher Risk of Cardiovascular Disease.” *GMS German Medical Science* 15: 1–8. https://doi.org/10.3205/000249.

Jensen, Jaclyn M., Pankaj C. Patel, and Jana L. Raver. 2014. “Is It Better to Be Average? High and Low Performance as Predictors of Employee Victimization.” *Journal of Applied Psychology* 99 (2): 296–309. https://doi.org/10.1037/a0034822.

Jonason, Peter K., Sarah Slomski, and Jamie Partyka. 2012. “The Dark Triad at Work: How Toxic Employees Get Their Way.” *Personality and Individual Differences* 52 (3): 449–53. https://doi.org/10.1016/j.paid.2011.11.008.

Kääriä, S., M. Laaksonen, O. Rahkonen, E. Lahelma, and P. Leino-Arjas. 2012. “Risk Factors of Chronic Neck Pain: A Prospective Study among Middle-Aged Employees.” *European Journal of Pain* 16 (6): 911–20. https://doi.org/10.1002/j.1532-2149.2011.00065.x.

Kacmar, K. Michele, Dennis P. Bozeman, Dawn S. Carlson, and William P. Anthony. 1999. “An Examination of the Perceptions of Organizational Politics Model: Replication and Extension.” *Human Relations* 52 (3): 383–416. https://doi.org/10.1177/001872679905200305.

Keashly, Loraleigh, and Steve Harvey. 2005. “Emotional Abuse in the Workplace.” In *Counterproductive Work Behavior: Investigations of Actors and Targets.*, 201–35. Washington: American Psychological Association. https://doi.org/10.1037/10893-009.

Kircher, Jan C, Cath Stilwell, Elizabeth Peffer Talbot, and Sandra Chesborough. 2011. “Academic Bullying in Social Work Departments: The Silent Epidemic.” *NACSW Convention 2011*, 1–29. https://pdfs.semanticscholar.org/bd00/02a688207f29d65f8e2ac68c2916c9571b30.pdf.

Kivimäki, Mika, Jane E. Ferrie, Eric Brunner, Jenny Head, Martin J. Shipley, Jussi Vahtera, and Michael G. Marmot. 2005. “Justice at Work and Reduced Risk of Coronary Heart Disease Among Employees.” *Archives of Internal Medicine* 165 (19): 2245. https://doi.org/10.1001/archinte.165.19.2245.

Kreis, Julia, and Wolfgang Bödeker. 2004. “Health-Related and Economic Benefits of Workplace Health Promotion and Prevention IGA-Report 3e.” Essen. www.iga-info.de.

Lanyon, Richard I., and Leonard D. Goodstein. 2003. “Validity and Reliability of a Pre-Employment Screening Test: The Counterproductive Behavior Index (CBI).” *Journal of Business and Psychology* 18 (4): 533–53. https://doi.org/10.1023/B:JOBU.0000028450.43358.15.

Leymann, Heinz. 1990. “Mobbing and Psychological Terror at Workplaces.” *Violence and Victims* 5: 119–26. https://www.mobbingportal.com/LeymannV&V1990(3).pdf.

———. 1996. “The Content and Development of Mobbing at Work.” *European Journal of Work and Organizational Psychology* 5 (2): 165–84. https://doi.org/10.1080/13594329608414853.

Leymann, Heinz, and Annelie Gustafsson. 1996. “Mobbing at Work and the Development of Post-Traumatic Stress Disorders.” *European Journal of Work and Organizational Psychology* 5 (2): 251–75. https://doi.org/10.1080/13594329608414858.

Leymann, Heinz, and U Tallgren. 1989. “Undersökning Av Frekvensen of Vuxenmobbning Inom SSAB Med Ett Nytt Frågeformulär [Investigation of the Frequency of Adult Mobbing at Work within SSAB with a New Survey Questionnaire].” *Arbete, Människa, Miljö* 1: 3–12.

Leymann, Heinz, and Dieter Zapf. 1990. “Mobbing and Psyhological Terror at Workplaces’ Violence and Victims.” *European Journal* 5 (2): 22. https://www.mobbingportal.com/LeymannV&V1990(3).pdf.

Lian, Huiwen, Douglas J Brown, D Lance Ferris, Lindie H Liang, Lisa M. Keeping, and Rachel Morrison. 2014. “Abusive Supervision and Retaliation: A Self-Control Framework.” *Academy of Management Journal* 57 (1): 116–39. https://doi.org/10.5465/amj.2011.0977.

Lim, Sandy, and Lilia M. Cortina. 2005. “Interpersonal Mistreatment in the Workplace: The Interface and Impact of General Incivility and Sexual Harassment.” *Journal of Applied Psychology* 90 (3): 483–96. https://doi.org/10.1037/0021-9010.90.3.483.

Lim, Sandy, Lilia M. Cortina, and Vicki J. Magley. 2008. “Personal and Workgroup Incivility: Impact on Work and Health Outcomes.” *Journal of Applied Psychology* 93 (1): 95–107. https://doi.org/10.1037/0021-9010.93.1.95.

Lusinyan, Lusine, and Leo Bonato. 2007. “Work Absence in Europe.” *IMF Staff Papers* 54 (3): 475–538. https://doi.org/10.1057/palgrave.imfsp.9450016.

Lutgen-Sandvik, Pamela. 2006. “Take This Job and … : Quitting and Other Forms of Resistance to Workplace Bullying.” *Communication Monographs* 73 (4): 406–33. https://doi.org/10.1080/03637750601024156.

Lutgen-Sandvik, Pamela, Sarah J Tracy, and Jess K Alberts. 2007. “Burned by Bullying in the American Workplace: Prevalence, Perception, Degree and Impact.” *Journal of Management Studies* 44 (6): 837–62. https://doi.org/10.1111/j.1467-6486.2007.00715.x.

Marchand, Alain, Andrée Demers, and Pierre Durand. 2005. “Does Work Really Cause Distress? The Contribution of Occupational Structure and Work Organization to the Experience of Psychological Distress.” *Social Science & Medicine* 61 (1): 1–14. https://doi.org/10.1016/j.socscimed.2004.11.037.

Martinko, Mark J., Michael J. Gundlach, and Scott C. Douglas. 2002. “Toward an Integrative Theory of Counterproductive Workplace Behavior: A Causal Reasoning Perspective.” *International Journal of Selection and Assessment* 10 (1&2): 36–50. https://doi.org/10.1111/1468-2389.00192.

Martino, Vittorio Di. 2009. “Workplace Violence in the Health Sector Country Case Studies Brazil, Bulgaria, Lebanon, Portugal, South Africa, Thailand and an Additional Australian Study. Synthesis Report.” https://www.who.int/violence_injury_prevention/violence/activities/workplace/WVsynthesisreport.pdf.

Mayer, David M., Stefan Thau, Kristina M. Workman, Marius Van Dijke, and David De Cremer. 2012. “Leader Mistreatment, Employee Hostility, and Deviant Behaviors: Integrating Self-Uncertainty and Thwarted Needs Perspectives on Deviance.” *Organizational Behavior and Human Decision Processes* 117 (1): 24–40. https://doi.org/10.1016/j.obhdp.2011.07.003.

McDermut, Jennifer Fine, David A. F. Haaga, and Lindsey Kirk. 2000. “An Evaluation of Stress Symptoms Associated with Academic Sexual Harassment.” *Journal of Traumatic Stress* 13 (3): 397–411. https://doi.org/10.1023/A:1007725022534.

McFarlin, Susan K., William Fals-Stewart, Debra A. Major, and Elaine M. Justice. 2001. “Alcohol Use and Workplace Aggression: An Examination of Perpetration and Victimization.” *Journal of Substance Abuse* 13 (3): 303–21. https://doi.org/10.1016/S0899-3289(01)00080-3.

Mintzberg, Henry. 1983. *Power in and around Organizations*. Prentice Hall.

Mitchell, Marie S, Ryan M Vogel, and Robert Folger. 2015. “Third Parties’ Reactions to the Abusive Supervision of Coworkers.” *Journal of Applied Psychology* 100 (4): 1040–55. https://doi.org/10.1037/apl0000002.

Namie, Gary. 2003. “Workplace Bullying: Escalated Incivility.” *Ivey Business Journal Online* nov/dec: 1–6.

Ng, Catalina Sau Man. 2019. “Effects of Workplace Bullying on Chinese Children’s Health, Behaviours and School Adjustment via Parenting: Study Protocol for a Longitudinal Study.” *BMC Public Health* 19 (1): 129. https://doi.org/10.1186/s12889-019-6458-1.

Nielsen, M. B., and S. Einarsen. 2012. “Prospective Relationships between Workplace Sexual Harassment and Psychological Distress.” *Occupational Medicine* 62 (3): 226–28. https://doi.org/10.1093/occmed/kqs010.

Nielsen, Morten Birkeland, and Ståle Valvatne Einarsen. 2018. “What We Know, What We Do Not Know, and What We Should and Could Have Known about Workplace Bullying: An Overview of the Literature and Agenda for Future Research.” *Aggression and Violent Behavior* 42 (July): 71–83. https://doi.org/10.1016/j.avb.2018.06.007.

Nielsen, Morten Birkeland, Jan Shahid Emberland, and Stein Knardahl. 2017. “Workplace Bullying as a Predictor of Disability Retirement.” *Journal of Occupational and Environmental Medicine* 59 (7): 609–14. https://doi.org/10.1097/JOM.0000000000001026.

Nielsen, Morten Birkeland, Anne-Marthe Rustad Indregard, and Simon Ã˜verland. 2016. “Workplace Bullying and Sickness Absence: A Systematic Review and Meta-Analysis of the Research Literature.” *Scandinavian Journal of Work, Environment & Health* 42 (5): 359–70. https://doi.org/10.5271/sjweh.3579.

Nielsen, Morten Birkeland, Ståle Pallesen, Anette Harris, and Ståle Valvatne Einarsen. 2018. “Protocol for a Systematic Review and Meta-Analysis of Research on the Associations between Workplace Bullying and Sleep.” *Systematic Reviews* 7 (1): 232. https://doi.org/10.1186/s13643-018-0898-z.

O’Leary-Kelly, Anne M, Ricky W Griffin, and David J Glew. 1996. “Organization-Motivated Aggression: A Research Framework.” *Academy of Management Review* 21 (1): 225–53. http://links.jstor.org/sici?sici=0363-7425%28199601%3A1%3C225%3AOAARF%3E2.0.CO%3B2-0.

O’Neill, Sharron, Nonna Martinov-Bennie, and Angela Cheung. 2013. “Issues in the Measurement and Reporting of Work Health and Safety Performance: A Review.” *International Governance and Performance Research Centre*. Macquarie University NSW. https://www.researchgate.net/publication/301283018_Issues_in_the_Measurement_and_Reporting_of_Work_Health_and_Safety_Performance.

O’Reilly, Jane, Sandra L. Robinson, Jennifer L. Berdahl, and Sara Banki. 2015. “Is Negative Attention Better than No Attention? The Comparative Effects of Ostracism and Harassment at Work.” *Organization Science* 26 (3): 774–93. https://doi.org/10.1287/orsc.2014.0900.

Örtqvist, Daniel, and Joakim Wincent. 2006. “Prominent Consequences of Role Stress: A Meta-Analytic Review.” *International Journal of Stress Management* 13 (4): 399–422. https://doi.org/10.1037/1072-5245.13.4.399.

Pallesen, Ståle, Morten B. Nielsen, Nils Magerøy, Cecilie S. Andreassen, and Ståle Einarsen. 2017. “An Experimental Study on the Attribution of Personality Traits to Bullies and Targets in a Workplace Setting.” *Frontiers in Psychology* 8 (JUN): 1–7. https://doi.org/10.3389/fpsyg.2017.01045.

Pearson, Christine, Lynne Andersson, and Christine Porath. 2005. “Workplace Incivility.” In *Counterproductive Workplace Behavior: Investigations of Actors and Targets*, edited by S. Fox and Paul E Spector, 177–200. Washington, D.C.: Adlerian Psychology Associates.

Pearson, Christine, and Christine Porath. 2009. *The Cost of Bad Behavior: How Incivility Is Damaging Your Business and What to Do about It.* Penguin Publishing Group. https://www.isbns.fm/isbn/9781591842613/.

Peng, He. 2011. “Dimensions and Measurement of Counterproductive Work Behaviors among Knowledge Workers.” *Journal of Management Science* 24: 12–22.

Porath, Christine L. 2015. “The Costs of Bad Behavior.” *Organizational Dynamics* 44 (4): 254–57. https://doi.org/10.1016/j.orgdyn.2015.09.001.

Porath, Christine, and Christine Pearson. 2013. “The Price of Incivility.” *Harvard Business Review*, 2013. https://hbr.org/2013/01/the-price-of-incivility.

Pouliakas, Konstantinos, and Ioannis Theodossiou. 2013. “The Economics of Health and Safety at Work: An Interdisciplinary Review of the Theory and Policy.” *Journal of Economic Surveys* 27 (1): 167–208. https://doi.org/10.1111/j.1467-6419.2011.00699.x.

Priesemuth, Manuela, and Marshall Schminke. 2019. “Helping Thy Neighbor? Prosocial Reactions to Observed Abusive Supervision in the Workplace.” *Journal of Management* 45 (3): 1225–51. https://doi.org/10.1177/0149206317702219.

Ray, Melinda Mercer. 2007. “The Dark Side of the Job: Violence in the Emergency Department.” *Journal of Emergency Nursing* 33 (3): 257–61. https://doi.org/10.1016/j.jen.2007.01.015.

Reknes, Iselin, Ståle Valvatne Einarsen, Johannes Gjerstad, and Morten Birkeland Nielsen. 2019. “Dispositional Affect as a Moderator in the Relationship between Role Conflict and Exposure to Bullying Behaviors.” *Frontiers in Psychology* 10 (JAN): 1–12. https://doi.org/10.3389/fpsyg.2019.00044.

Reknes, Iselin, Ståle Pallesen, Nils Magerøy, Bente Elisabeth Moen, Bjørn Bjorvatn, and Ståle Einarsen. 2014. “Exposure to Bullying Behaviors as a Predictor of Mental Health Problems among Norwegian Nurses: Results from the Prospective SUSSH-Survey.” *International Journal of Nursing Studies* 51 (3): 479–87. https://doi.org/10.1016/j.ijnurstu.2013.06.017.

Ren, Dongning, Eric Wesselmann, and Kipling D. Williams. 2016. “Evidence for Another Response to Ostracism.” *Social Psychological and Personality Science* 7 (3): 204–12. https://doi.org/10.1177/1948550615616169.

Riadi, S.S., Hendryadi, and I. Tricahyadinata. 2019. “Workplace Incivility, Self-Efficacy, and Turnover Intention Relationship Model: A Multi-Group Analysis.” *Russian Journal of Agricultural and Socio-Economic Sciences* 85 (1): 358–68. https://doi.org/10.18551/rjoas.2019-01.44.

Richman, J A, K M Rospenda, S J Nawyn, J A Flaherty, M Fendrich, M L Drum, and T P Johnson. 1999. “Sexual Harassment and Generalized Workplace Abuse among University Employees: Prevalence and Mental Health Correlates.” *American Journal of Public Health* 89 (3): 358–63. https://doi.org/10.2105/AJPH.89.3.358.

Ridenour, Marilyn, Marilyn Lanza, Scott Hendricks, Dan Hartley, Jill Rierdan, Robert Zeiss, and Harlan Amandus. 2015. “Incidence and Risk Factors of Workplace Violence on Psychiatric Staff.” *Work* 51 (1): 19–28. https://doi.org/10.3233/WOR-141894.

Rioux, Phanie, and Secor-Paris Marie-Élène Roberge. 2005. “Classification Des Comportements Antisociaux Au Travail (Classification of Anti-Social Behaviour at Work).” *Interactions* 9 (1): 63–88. https://www.usherbrooke.ca/psychologie/fileadmin/sites/psychologie/espace-etudiant/Revue_Interactions/Volume_9_no_1/V9N1_RIOUX_ROBERGE_BRUNET_SAVOIE_COURCY_p63-88.pdf.

Riva, Paolo, Lorenzo Montali, James H. Wirth, Simona Curioni, and Kipling D. Williams. 2017. “Chronic Social Exclusion and Evidence for the Resignation Stage.” *Journal of Social and Personal Relationships* 34 (4): 541–64. https://doi.org/10.1177/0265407516644348.

Robinson, Richard N.S. 2008. “Revisiting Hospitality’s Marginal Worker Thesis: A Mono-Occupational Perspective.” *International Journal of Hospitality Management* 27 (3): 403–13. https://doi.org/10.1016/j.ijhm.2007.09.003.

Robinson, Sandra L., and Rebecca J. Bennett. 1995. “A Typology of Deviant Workplace Behaviors: A Multidimensional Scaling Study.” *Academy of Management Journal* 38 (2): 555–72. https://doi.org/10.5465/256693.

Robinson, Sandra L., Wei Wang, and Christian Kiewitz. 2014. “Coworkers Behaving Badly: The Impact of Coworker Deviant Behavior upon Individual Employees.” *Annual Review of Organizational Psychology and Organizational Behavior* 1 (1): 123–43. https://doi.org/10.1146/annurev-orgpsych-031413-091225.

Rodwell, John, and Defne Demir. 2012. “Oppression and Exposure as Differentiating Predictors of Types of Workplace Violence for Nurses.” *Journal of Clinical Nursing* 21 (15–16): 2296–2305. https://doi.org/10.1111/j.1365-2702.2012.04192.x.

Rogers, Kimberley-Ann, and E. Kevin Kelloway. 1997. “Violence at Work: Personal and Organizational Outcomes.” *Journal of Occupational Health Psychology* 2 (1): 63–71. https://doi.org/10.1037/1076-8998.2.1.63.

Rospenda, Kathleen M, Judith A Richman, and Candice A Shannon. 2009. “Prevalence and Mental Health Correlates of Harassment and Discrimination in the Workplace.” *Journal of Interpersonal Violence* 24 (5): 819–43. https://doi.org/10.1177/0886260508317182.

Sabbath, Erika L., Jessica A.R. Williams, Leslie I. Boden, Tommaso Tempesti, Gregory R. Wagner, Karen Hopcia, Dean Hashimoto, and Glorian Sorensen. 2018. “Mental Health Expenditures: Association with Workplace Incivility and Bullying among Hospital Patient Care Workers.” *Journal of Occupational and Environmental Medicine* 60 (8): 737–42. https://doi.org/10.1097/JOM.0000000000001322.

Sarfraz, Muddassar, Wang Qun, Ambreen Sarwar, Muhammad Ibrahim Abdullah, Muhammad Kashif Imran, and Imran Shafique. 2019. “Mitigating Effect of Perceived Organizational Support on Stress in the Presence of Workplace Ostracism in the Pakistani Nursing Sector.” *Psychology Research and Behavior Management* Volume 12 (September): 839–49. https://doi.org/10.2147/PRBM.S210794.

Schat, Aaron C. H., and E. Kevin Kelloway. 2003. “Reducing the Adverse Consequences of Workplace Aggression and Violence: The Buffering Effects of Organizational Support.” *Journal of Occupational Health Psychology* 8 (2): 110–22. https://doi.org/10.1037/1076-8998.8.2.110.

Schat, Aaron C.H., and Kevin E Kelloway. 2005. “Workplace Aggression.” In *Handbook of Work Stress*, edited by Julian Barling, E Kevin Kelloway, and Michael R. Frone, 189–218. Thousand Oaks: Sage.

Skarlicki, Daniel P, and Robert Folger. 1997. “Retaliation in the Workplace: The Roles of Distributive, Procedural, and Interactional Justice.” *Journal of Applied Psychology* 82 (3): 434–43. https://doi.org/10.1037/0021-9010.82.3.434.

Sliter, Katherine A., Michael T. Sliter, Scott A. Withrow, and Steve M. Jex. 2012. “Employee Adiposity and Incivility: Establishing a Link and Identifying Demographic Moderators and Negative Consequences.” *Journal of Occupational Health Psychology* 17 (4): 409–24. https://doi.org/10.1037/a0029862.

Sliter, Michael, Steve Jex, Katherine Wolford, and Joanne McInnerney. 2010. “How Rude! Emotional Labor as a Mediator between Customer Incivility and Employee Outcomes.” *Journal of Occupational Health Psychology* 15 (4): 468–81. https://doi.org/10.1037/a0020723.

Sliter, Michael T., Shuang Yueh Pui, Katherine A. Sliter, and Steve M. Jex. 2011. “The Differential Effects of Interpersonal Conflict from Customers and Coworkers: Trait Anger as a Moderator.” *Journal of Occupational Health Psychology* 16 (4): 424–40. https://doi.org/10.1037/a0023874.

Spector, Paul E., Peter Y. Chen, and Brian J. O’Connell. 2000. “A Longitudinal Study of Relations between Job Stressors and Job Strains While Controlling for Prior Negative Affectivity and Strains.” *Journal of Applied Psychology* 85 (2): 211–18. https://doi.org/10.1037/0021-9010.85.2.211.

Spector, Paul E., and Suzy Fox. 2005. “The Stressor-Emotion Model of Counterproductive Work Behavior.” In *Counterproductive Work Behavior: Investigations of Actors and Targets.*, edited by Suzy Fox and Paul E Spector, 151–74. Washington: American Psychological Association. https://doi.org/10.1037/10893-007.

Sperry, Len, and Maureen Duffy. 2009. “Workplace Mobbing: Family Dynamics and Therapeutic Considerations.” *The American Journal of Family Therapy* 37 (5): 433–42. https://doi.org/10.1080/01926180902945756.

Tangirala, Subrahmaniam, and Bradley J. Alge. 2006. “Reactions to Unfair Events in Computer-Mediated Groups: A Test of Uncertainty Management Theory.” *Organizational Behavior and Human Decision Processes* 100 (1): 1–20. https://doi.org/10.1016/j.obhdp.2005.11.002.

Tepper, Bennett J. 2007. “Abusive Supervision in Work Organizations: Review, Synthesis, and Research Agenda.” *Journal of Management* 33 (3): 261–89. https://doi.org/10.1177/0149206307300812.

Tepper, Bennett J., Michelle K. Duffy, Henle Christine A., and Lambert Lisa Schurer. 2006. “Procedural Injustice, Victim Precipitation, and Abusive Supervision.” *Personnel Psychology* 59 (1): 101–23. https://doi.org/10.1111/j.1744-6570.2006.00725.x.

Thau, Stefan, Rebecca J. Bennett, Marie S. Mitchell, and Mary Beth Marrs. 2009. “How Management Style Moderates the Relationship between Abusive Supervision and Workplace Deviance: An Uncertainty Management Theory Perspective.” *Organizational Behavior and Human Decision Processes* 108 (1): 79–92. https://doi.org/10.1016/j.obhdp.2008.06.003.

Theorell, Töres, Anne Hammarström, Gunnar Aronsson, Lil Träskman Bendz, Tom Grape, Christer Hogstedt, Ina Marteinsdottir, Ingmar Skoog, and Charlotte Hall. 2015. “A Systematic Review Including Meta-Analysis of Work Environment and Depressive Symptoms.” *BMC Public Health* 15 (1): 738. https://doi.org/10.1186/s12889-015-1954-4.

Trépanier, Sarah-Geneviève, Claude Fernet, and Stéphanie Austin. 2015. “A Longitudinal Investigation of Workplace Bullying, Basic Need Satisfaction, and Employee Functioning.” *Journal of Occupational Health Psychology* 20 (1): 105–16. https://doi.org/10.1037/a0037726.

Tynes, Tore, Håkon A. Johannessen, and Tom Sterud. 2013. “Work-Related Psychosocial and Organizational Risk Factors for Headache.” *Journal of Occupational and Environmental Medicine* 55 (12): 1436–42. https://doi.org/10.1097/JOM.0b013e3182a7e678.

Valle, Matthew, and Pamela L. Perrewe. 2000. “Do Politics Perceptions Relate to Political Behaviors? Tests of an Implicit Assumption and Expanded Model.” *Human Relations* 53 (3): 359–86. https://doi.org/10.1177/0018726700533004.

Vardi, Y., and E. Weitz. 2004. *Misbehavior in Organizations*. Mahwah, NJ: Erlbaum.

Verkuil, Bart, Serpil Atasayi, and Marc L Molendijk. 2015. “Workplace Bullying and Mental Health : A Meta- Analysis on Cross-Sectional and Longitudinal Data Workplace Bullying and Mental Health : A Meta-Analysis on Cross-Sectional and Longitudinal Data.” *PLoS ONE* 10 (8): 1–17. https://doi.org/10.1371/journal. pone0135225.

Vigoda-Gadot, Eran, and Danit Kapun. 2005. “Perceptions of Politics and Perceived Performance in Public and Private Organisations: A Test of One Model across Two Sectors.” *Policy & Politics* 33 (2): 251–76. https://doi.org/10.1332/0305573053870185.

Vigoda, Eran. 2001. “Reactions to Organizational Politics: A Cross-Cultural Examination in Israel and Britain.” *Human Relations* 54 (11): 1483–1518. https://doi.org/10.1177/00187267015411004.

Waschgler, Kathrin, José Antonio Ruiz-Hernández, Bartolomé Llor-Esteban, and Mariano García-Izquierdo. 2013. “Patients’ Aggressive Behaviours towards Nurses: Development and Psychometric Properties of the Hospital Aggressive Behaviour Scale- Users.” *Journal of Advanced Nursing* 69 (6): 1418–27. https://doi.org/10.1111/jan.12016.

Williams, Kipling D. 2001. *Ostracism the Power of Sillence.* Edited by Peter Salovey. 9th ed. New York, London: The Guilford Press.

Wolmerath, Martin. 2019. *Mobbing. Rechtshandbuch Fur Die Praxis ( Mobbing. Legal Handbook for Practice)*. 5th ed. Baden-Baden: NomosPraxis. http://dnb.d-nb.

Wright, Bradley R. E., Avshalom Caspi, Terrie E. Moffitt, and Ray Paternoster. 2004. “Does the Perceived Risk of Punishment Deter Criminally Prone Individuals? Rational Choice, Self-Control, and Crime.” *Journal of Research in Crime and Delinquency* 41 (2): 180–213. https://doi.org/10.1177/0022427803260263.

Wu, Chia-Huei, Jun Liu, Ho Kwong Kwan, and Cynthia Lee. 2016. “Why and When Workplace Ostracism Inhibits Organizational Citizenship Behaviors: An Organizational Identification Perspective.” *Journal of Applied Psychology* 101 (3): 362–78. https://doi.org/10.1037/apl0000063.

Wu, Long-Zeng, Frederick Hong-kit Yim, Ho Kwong Kwan, and Xiaomeng Zhang. 2012. “Coping with Workplace Ostracism: The Roles of Ingratiation and Political Skill in Employee Psychological Distress.” *Journal of Management Studies* 49 (1): 178–99. https://doi.org/10.1111/j.1467-6486.2011.01017.x.

Xu, Tianwei, Linda L. Magnusson Hanson, Theis Lange, Liis Starkopf, Hugo Westerlund, Ida E. H. Madsen, Reiner Rugulies, et al. 2018. “Workplace Bullying and Violence as Risk Factors for Type 2 Diabetes: A Multicohort Study and Meta-Analysis.” *Diabetologia* 61 (1): 75–83. https://doi.org/10.1007/s00125-017-4480-3.

Yperen, Nico W. Van, Mariet Hagedoorn, and Sabine Geurts. 1996. “Intent to Leave and Absenteeism as Reactions to Perceived Inequity: The Role of Psychological and Social Constraints.” *Journal of Occupational and Organizational Psychology* 69: 367–72. https://repository.ubn.ru.nl/handle/2066/28712.

Zamperini, Adriano, and Marialuisa Menegatto. 2013. “La Violenza Collettiva e Il G8 Di Genova. Trauma Psicopolitico e Terapia Sociale Della Testimonianza.” *Psicoterapia e Scienze Umane*, no. 3 (September): 423–42. https://doi.org/10.3280/PU2013-003002.

Zapf, Dieter, and Claudia Gross. 2001. “Conflict Escalation and Coping with Workplace Bullying: A Replication and Extension.” *European Journal of Work and Organizational Psychology* 10 (4): 497–522. https://doi.org/10.1111/j.1600-0587.1996.tb01264.x.

Zhao, Hongdan, Zhenglong Peng, and Geoff Sheard. 2013. “Workplace Ostracism and Hospitality Employees’ Counterproductive Work Behaviors: The Joint Moderating Effects of Proactive Personality and Political Skill.” *International Journal of Hospitality Management* 33 (1): 219–27. https://doi.org/10.1016/j.ijhm.2012.08.006.
