# Supplementary material for: A Systematic Review of Negative Work Behavior: Toward an Integrated Definition
Source: Front Psychol. 2021 Oct 27;12:726973. doi: 10.3389/fpsyg.2021.726973 (PMC8578924; doi:10.3389/fpsyg.2021.726973)
Supplement: Supplementary file 3 [file Table_3.docx]

**Supplementary**

**TABLE 3 |** C. Actor types and roles in NWB labels derived from study titles

| **Construct NWB** | **Individual actors: C1. Stranger C2. Co-worker/manager C3. Customer, client, pupil C4. Relative**  **C5. Group/networks Roles: in Italic** |
| --- | --- |
| Aggression C  C  C  C  C | 1. third parties such as audience, citizens are a key factor in escalation (Felson 2006), ﻿citizens (Fredericksen and McCorkle 2013), direct are actors, indirect are bystanders (Dupré, Dawe, and Barling 2014) 2. workers and ex-workers (Baron and Neuman 1998), within organization (Schat and Kelloway 2005) 3. customer related aggression (Dormann and Zapf 2004), prisoners (*changing roles of target and offender)* (Ireland, 2013), clients in escalating role (Felson 2006) 4. ﻿patients’ relatives (Deery, Walsh, and Guest 2011) 5. groups and organizations can be aggressors and targets (Pinto 2014),﻿ complex interactions in group members between multiple intrapersonal and environmental variables (Brees, Mackey, and Martinko 2013) |
| Bullying C C  C  C  C | 1. ﻿strangers (in cyber bullying; Kavitha and Bhuvaneswari 2016) 2. organizational members (De Cuyper, Baillien, and De Witte 2009), team-level conflicts (Zahlquist et al. 2019; Olthof and Goossens 2008; Salmivalli et al. 1998; Goossens, Olthof, and Dekker 2006), supervisor and manager as perpetrators (Zapf et al. 2003), vertical/ downwards bullying by managers (Parzefall and Salin 2010), staff (Berry et al. 2012), teachers (Fahie and Devine 2014) 3. pupils (Fahie and Devine 2014), patient (Berry et al. 2012),﻿ victims, witnesses, bystanders who offer support or castigate perpetrators, and officials tasked with investigating reported grievances (Helge Hoel et al. 2009), clients, customers, patients, students (Török et al. 2016) 4. social networks with *participant roles as followers* (Broersen, Ossenblok, and Montesano Montessori 2015), *assistants, defenders, reinforcers or outsiders* (Huitsing and Veenstra 2012), family (Berry et al. 2012), friends, contacts (Kavitha and Bhuvaneswari 2016) 5. one or more persons (De Cuyper, Baillien, and De Witte 2009), restricted to the organizational level or, in some cases, to economic forces and globalization (Helge Hoel, Cooper, and Faragher 2001), multiple perpetrators (Glambek, Skogstad, and Einarsen 2020) |
| Mobbing C C  C  C  C | 1. - 2. organizational members, school members (González Trijueque and Graña Gómez 2010), colleagues, boss (Hubert and van Veldhoven 2001) 3. - 4. intrusion into private persons (Sperry 2009), husband, children (Pheko 2018) 5. victim, mobber and witness (Maran, Bernardelli, and Varetto 2018), groups of employees (Takaki, Taniguchi, and Hirokawa 2013), more than one co-worker against a colleague (Friedenberg 2008), mutual actors in a group (Leymann and Tallgren 1989), organizational groups (Sperry 2009) |
| Harassment C  /Discrimination C  C  C  C | 1. legal system as actor in procedural harassment (Clemente et al. 2019), public, strangers (McCarty, Iannone, and Kelly 2014) 2. employees: co-workers, supervisors (Grandey, Kern, and Frone 2007), organization members as perpetrator, target and witness / bystander (Neall and Tuckey 2014) 3. customers (Grandey, Kern, and Frone 2007; LeBlanc and Kelloway 2002), ﻿customer sexual harassment (Yagil, 2008), students (Benya, Widnall, and Johnson 2018; H Hoel, Giga, and Davidson 2007) 4. institutional harassment by the legal system on family members (Clemente et al. 2019) 5. in workgroups and departments ﻿reflecting the shared environmental properties (James et al., 2008), perpetrators, victims, witnesses or a narrow group of those (Russo et al. 2008), |
| Deviance C  C  C  C  C | 1. co-traffic users of road workers such as in speeding (Sheahan and Smith 2003), ﻿actors around the workplace to work with i.e. recreational drug use (Lucas and Friedrich 2005) 2. organizational members, workplace actors (Bashir et al. 2019; Robinson and Bennett 1995) 3. the target in service sabotage is the customer (Harris & Ogbonna, 2006), passengers (Sheahan and Smith 2003)﻿, external constituents of the organization, such as customers and suppliers (Galperin 2012) 4. - 5. the social audience rather than the individual actor determines whether or not behavior is labelled deviant (Erikson 1987), among occupational groups (Sheahan and Smith 2003) |
| Counterproductive C  Work Behavior (CWB) C  C  C  C | 1. - 2. ﻿organization (CWBO), its members (CWBI / CWBP), or both (Robinson and Bennett 1995), employees (de Jonge and Peeters 2009), workers: co-workers, supervisors (Spector and Fox 2005) 3. clients, customers, patients (de Jonge and Peeters 2009), clients, public (Gruys and Sackett 2003), stakeholders, clients, customers (Spector and Fox 2005), students (Lucas and Friedrich 2005) 4. - 5. inter-, intra-personal, groups (O’Boyle, Forsyth, and O’Boyle 2011) |
| Violence C  C  C  C  C | 1. stranger with criminal intent (Injury Prevention Research Centre 2001; Teymourzadeh et al. 2014), interpersonal stranger violence (Agrawal et al. 2019), public violence (Scalora et al. 2003) 2. worker-on-worker (Injury Prevention Research Centre 2001), staff, management (Teymourzadeh et al. 2014) horizontal (between co-workers -vertical (between supervisor-workers) (Fernandez et al. 2017) 3. customer/client (Injury Prevention Research Centre 2001), client violence ﻿(Macdonald and Sirotich 2005) 4. a personal relationship (Injury Prevention Research Centre 2001), relatives (Peng et al. 2018) 5. ﻿a group or a community (Campo and Klijn 2018) |
| Abuse C  C  C  C  C | 1. outsiders (Grandey, Kern, and Frone 2007) 2. organization members (Keashly and Harvey 2005), insiders (Grandey, Kern, and Frone 2007), school staff (Braverman 2007) 3. customers (Grandey, Kern, and Frone 2007) 4. ﻿patient’ relatives (Öztunç 2006) 5. one or more other persons, including management (Aquino 2000) |
| Terror C C  C  C  C | 1. terrorist attack by organizational outsiders (Birkeland et al. 2017), terrorism (Nissen et al. 2019) 2. staff in working life (Leymann and Zapf 1990) 3. pupils (Haviland 2008) 4. all people in the organization surrounding perpetrator and target (Kant et al. 2013) 5. individuals or subnational groups, through the intimidation of a large audience (Brandt and Sandler 2010), specific groups of people (Gee 1996), one or a number of persons (Leymann and Zapf 1990) |
| Injustice C C  C  C  C | 1. strangers on internet (Citron and Franks 2014) 2. actors in the workplace (Rizvi, Friedman, and Azam 2017) 3. discrimination of actors of demographic minority groups at the institutional level (Okechukwu et al. 2014), specific groups (e.g. women, race) embedded in institutions (e.g. education, social work) of society (Fahrenwald et al. 2007)﻿ 4. - 5. at the interpersonal and institutional level (Okechukwu et al. 2014), between ethnic groups (van der Leun and van der Woude 2011) ﻿ |
| Interpersonal conflict C C  C  C  C | 1. - 2. supervisors or co-workers (e.g., Bruk-Lee & Spector, 2006; Frone, 2000), counter-aggression (Lee and Brotheridge, 2006), parties involved in hostile workplace interactions (Burton and Hoobler, 2006; Lutgen-Sandvik et al., 2007),﻿ within that unit, or between that unit and relevant outsiders such as other units within the organization, clients, and the like (Nauta, De Dreu, and Van Der Vaart 2002), co-workers (Sliter, Pui, Sliter, & Jex, 2011) 3. ﻿customer verbal aggression (Grandey, Dickter, and Sin, 2004), customer interpersonal conflict ( Sliter et al., 2011), clients, and the like (Nauta, De Dreu, and Van Der Vaart 2002) 4. - 5. between two or more people (Einarsen et al., 2003), individual or group perceives differences and opposition between oneself and another individual or group about interests, beliefs or values that matter to them (De Dreu, Harinck, and Van Vianen 1999) |
| Victimization C  /Scapegoating  C    C  C  C | 1. public in the school context (Astor et al. 2014), peers, adults in different systems (school, community, family) (Ferrer et al. 2011), violence and aggression by public (Friis et al. 2020) 2. other organizational persons (on behalf the scapegoat/victim) (Aquino et al., 1999), employees in the work setting (Nielsen and Einarsen 2012; Snyder 2012), one or more members of an organization (Aquino and Thau 2009), perpetrator and victim among students (Reyns, Henson, and Fisher 2012) 3. clients in a practice setting (Ringstad 2005) 4. relatives (Ferrer et al. 2011) 5. formal and informal organizational social network of actor positions (Lamertz and Aquino 2004), scapegoating as victimization within the workgroup (Hutchinson 2013) |
| Micropolitics C C  C  C  C | 1. public in and around organizations, social responsible actors (Mintzberg, Etzion, and Saku 2019) 2. actors in the organization, workplace (Vigoda 2001; Zhou and Ferris 1995), co-workers and supervisors (Atinc, Fuller, and Darrat 2010) 3. actors in the external business environment (Baysinger 1984) 4. - 5. Individual versus group of actors (Mintzberg 1983), one or more members of an organization (Aquino and Thau 2009), organizational politics: actors in the system perspective (Cyert and March 1963), individuals, groups, organizations attempt to influence others as actors (Drory and Romm 1990) |
| Ostracism C C  C  C  C | 1. - 2. employees (Fiset, Al Hajj, and Vongas 2017), in organization and work group (Balliet and Ferris 2013) 3. clients from welfare work (Saunders and Wong 2009), an external source such as an informant (Williams 2007), complying with clients to reduce ostracism (Carter‐Sowell, Chen, and Williams 2008), ostracizees’ social support of others (Fiset, Al Hajj, and Vongas 2017) 4. - 5. groups and dyads (Faulkner, Williams, Sherman & Williams, 1997), group-level phenomenon (Balliet and Ferris 2013), ingroup/outgroup (Gonsalkorale and Williams 2007) |
| Incivility C C  C  C  C | 1. consumer-provider relationships (Sliter et al. 2010), visitors (Hershcovis and Reich 2013) 2. workplace, co-workers, managers (Hershcovis and Barling 2010; Schilpzand, De Pater, and Erez 2016) 3. customer, client (Wilson and Holmvall 2013; Sliter et al. 2010; Schilpzand, De Pater, and Erez 2016), patients (Hershcovis and Reich 2013), clients (Walsh et al. 2020), customers (Grandey, Dickter, and Sin 2004) 4. actors in organization with non-employee status such as deliverers (Holroyd-Leduc and Straus 2018; Pattani et al. 2018) 5. workers in organization level group (Griffin 2010), personal level to group level (Lim et al., 2008) |
| Social safety C C  C  C  C | 1. criminal (National Institute for Occupational Safety and Health 2013; Merchant and Lundell 2001) 2. co-workers, managers (National Institute for Occupational Safety and Health 2013; Merchant and Lundell 2001) 3. customer/client (National Institute for Occupational Safety and Health 2013; Merchant and Lundell 2001) 4. ﻿bystanders (in roles as *outsiders, endorsers, or followers*)(Broersen, Ossenblok, and Montesano Montessori 2015), personal relations (National Institute for Occupational Safety and Health 2013; Merchant and Lundell 2001) 5. pupils and staff (Sijbers et al. 2014)**,** employees, patients (McLindon, Humphreys, and Hegarty 2018) |

# References Table 5 | C. Actor types and roles in NWB labels derived from study titles

Agrawal, Priyanka, Yousra Yusuf, Omrana Pasha, Shahmir H. Ali, Homayra Ziad, and Adnan A. Hyder. 2019. “Interpersonal Stranger Violence and American Muslims: An Exploratory Study of Lived Experiences and Coping Strategies.” *Global Bioethics* 30 (1): 28–42. https://doi.org/10.1080/11287462.2019.1683934.

Aquino, Karl. 2000. “Structural and Individual Determinants of Workplace Victimization: The Effects of Hierarchical Status and Conflict Management Style.” *Journal of Management* 26 (2): 171–93. https://doi.org/10.1177/014920630002600201.

Aquino, Karl, and Stefan Thau. 2009. “Workplace Victimization: Aggression from the Target’s Perspective.” *Annual Review of Psychology* 60 (1): 717–41. https://doi.org/10.1146/annurev.psych.60.110707.163703.

Astor, Ron Avi, Rami Benbenishty, Roxana Marachi, and Heather Ann Meyer. 2014. “The Social Context of Schools: Monitoring and Mapping Student Victimization in Schools.” In *Handbook of School Violence and School Safety: From Research to Practice*, edited by S. R Jimerson and Furlong. M. J, 221–33. Mahwah, NJ: Erlbaum.

Atinc, Guclu, Jerry Bryan Fuller, and Mahmoud A Darrat. 2010. “Perceptions of Organizational Politics: A Meta-Analysis of Theoretical Antecedents.” *Journal of Managerial Issues* XXII (4): 494–513. https://doi.org/10.2307/25822527.

Aydin, Nilüfer, Peter Fischer, and Dieter Frey. 2010. “Turning to God in the Face of Ostracism: Effects of Social Exclusion on Religiousness.” *Personality and Social Psychology Bulletin* 36 (6): 742–53. https://doi.org/10.1177/0146167210367491.

Balliet, Daniel, and D.Lance Ferris. 2013. “Ostracism and Prosocial Behavior: A Social Dilemma Perspective.” *Organizational Behavior and Human*

*Decision Processes* 120 (2): 298–308. https://doi.org/10.1016/j.obhdp.2012.04.004.

Baron, R. A., and J. H. Neuman. 1998. “Workplace Violence and Workplace Aggression: Evidence Concerning Specific Forms, Potential Causes, and Preferred Targets.” *Journal of Management* 24 (3): 391–419. https://doi.org/10.1177/014920639802400305.

Bashir, Mohsin, Muhammad Abrar, Muhammad Yousaf, Shahnawaz Saqib, and Rizwan Shabbir. 2019. “Organizational Politics and Workplace Deviance In Unionized Settings: Mediating Role of Job Stress and Moderating Role of Resilience.” *Psychology Research and Behavior Management* Volume 12 (October): 943–59. https://doi.org/10.2147/PRBM.S213672.

Baysinger, Barry D. 1984. “Domain Maintenance as an Objective of Business Political Activity: An Expanded Typology.” *Academy of Management Review* 9 (2): 248–58. https://doi.org/10.5465/amr.1984.4277642.

Benya, F, S E Widnall, and Paul D. Johnson. 2018. *Sexual Harassment of Women*. Edited by Paula A. Johnson, Sheila E. Widnall, and Frazier F. Benya. *Sexual Harassment of Women*. Washington, D.C.: National Academies Press. https://doi.org/10.17226/24994.

Berry, Peggy A., Gordon L. Gillespie, Donna Gates, and John Schafer. 2012. “Novice Nurse Productivity Following Workplace Bullying.” *Journal of Nursing Scholarship* 44 (1): 80–87. https://doi.org/10.1111/j.1547-5069.2011.01436.x.

Birkeland, Marianne Skogbrott, Morten Birkeland Nielsen, Marianne Bang Hansen, Stein Knardahl, and Trond Heir. 2017. “The Impact of a Workplace Terrorist Attack on Employees’ Perceptions of Leadership: A Longitudinal Study from Pre- to Postdisaster.” *The Leadership Quarterly* 28 (5): 659–71. https://doi.org/10.1016/j.leaqua.2017.01.002.

Brandt, P. T., and T. Sandler. 2010. “What Do Transnational Terrorists Target? Has It Changed? Are We Safer?” *Journal of Conflict Resolution*.

Braverman, Mark. 2007. “Abuse and Violence in the Workplace and School.” *Journal of Emotional Abuse* 4 (3–4): 1–11. https://doi.org/10.1300/J135v04n03_01.

Brees, Jeremy R., Jeremy Mackey, and Mark J. Martinko. 2013. “An Attributional Perspective of Aggression in Organizations.” Edited by Raymond T. Lee. *Journal of Managerial Psychology* 28 (3): 252–72. https://doi.org/10.1108/02683941311321150.

Broersen, Annemiek, Anja Ossenblok, and Nicolien Montesano Montessori. 2015. “Pesten En Sociale Veiligheid Op Scholen: Definities En Keuzes Op Micro-, Meso- En Macroniveau [ Bullying and Social Safety in Schools: Definitions and Choices at Micro, Meso and Macro Level].” In *Tijdschrift Voor Orthopedagogiek*, 54:108–19. http://schoolpsychologencongres.nl/images/presentaties/Bijbehorend-artikel-wokshop-Annemiek-Broersen.pdf.

Burns, Tom. 1961. “Micropolitics: Mechanisms of Institutional Change.” *Administrative Science Quarterly* 6 (3): 257. https://doi.org/10.2307/2390703.

Campo, Varinia Rodríguez, and Tatiana Paravic Klijn. 2018. “Verbal Abuse and Mobbing in Pre-Hospital Care Services in Chile.” *Revista Latino-Americana de Enfermagem* 25 (January): e2956. https://doi.org/10.1590/1518-8345.2073.2956.

Carter‐Sowell, Adrienne R., Zhansheng Chen, and Kipling D. Williams. 2008. “Ostracism Increases Social Susceptibility.” *Social Influence* 3 (3): 143–53. https://doi.org/10.1080/15534510802204868.

Citron, Danielle Keats, and Mary Anne Franks. 2014. “Criminalizing Revenge Porn.” *Wake Forest Law Review* 49 (2014–1): 345. https://scholarship.law.bu.edu/faculty_scholarship/643.

Clemente, Miguel, Dolores Padilla-Racero, Pablo Espinosa, Adela Reig-Botella, and Manuel Gandoy-Crego. 2019. “Institutional Violence against Users of the Family Law Courts and the Legal Harassment Scale.” *Frontiers in Psychology* 10 (JAN): 1–8. https://doi.org/10.3389/fpsyg.2019.00001.

Cuyper, Nele De, Elfi Baillien, and Hans De Witte. 2009. “Job Insecurity, Perceived Employability and Targets’ and Perpetrators’ Experiences of Workplace Bullying.” *Work & Stress* 23 (3): 206–24. https://doi.org/10.1080/02678370903257578.

Cyert, R. M., and J. G. March. 1963. *A Behavioral Theory of the Firm.* 2(4). Englewood Cliffs N.J.

Deery, Stephen, Janet Walsh, and David Guest. 2011. “Workplace Aggression: The Effects of Harassment on Job Burnout and Turnover Intentions.” *Work, Employment and Society* 25 (4): 742–59. https://doi.org/10.1177/0950017011419707.

Dormann, Christian, and Dieter Zapf. 2004. “Customer-Related Social Stressors and Burnout.” *Journal of Occupational Health Psychology* 9 (1): 61–82. https://doi.org/10.1037/1076-8998.9.1.61.

Dreu, C. K. W. De, F. Harinck, and A. E. M. Van Vianen. 1999. “Conflict and Performance in Groups and Organizations.” In *International Review of Industrial and Organizational Psychology*, edited by C. L. Cooper and I. T. Robertson, 369–414. John Wiley & Sons Ltd.

Drory, Amos, and Tsilia Romm. 1990. “The Definition of Organizational Politics: A Review.” *Human Relations* 43 (11): 1133–54. https://doi.org/10.1177/001872679004301106.

Dupré, Kathryne E., Kimberly-Anne Dawe, and Julian Barling. 2014. “Harm to Those Who Serve.” *Journal of Interpersonal Violence* 29 (13): 2355–77. https://doi.org/10.1177/0886260513518841.

Einarsen, S, Helge Hoel, Dieter & Zapf, and Cary Lynn Cooper. 2003. *Bullying and Emotional Abuse in the Workplace : International Perspectives in Research and Practice*. Edited by Ståle Einarsen, Helge Hoel, Dieter Zapf, and Cary L. Cooper. 1st ed. London ; New York: Taylor & Francis. https://books.google.es/books.

Erikson, K. T. 1987. “Notes on the Sociology of Deviance.” In *Deviance: The Interactionist Perspective*, edited by Earl Rubington and Martin S. Weinberg, Macmillan. New York.

Fahie, Declan, and Dympna Devine. 2014. “The Impact of Workplace Bullying on Primary School Teachers and Principals.” *Scandinavian Journal of Educational Research* 58 (2): 235–52. https://doi.org/10.1080/00313831.2012.725099.

Fahrenwald, Nancy L., Janette Y. Taylor, Shawn M. Kneipp, and Mary K. Canales. 2007. “Academic Freedom and Academic Duty to Teach Social Justice: A Perspective and Pedagogy for Public Health Nursing Faculty.” *Public Health Nursing* 24 (2): 190–97. https://doi.org/10.1111/j.1525-1446.2007.00624.x.

Felson, Richard B. 2006. “Violence as Instrumental Behavior.” In *Handbook of Workplace Violence*, edited by Kevin Kelloway, Julian Barling, and R Hurrell, Jr. Joseph, 1st ed., 7–29. Thousand Oaks, London, New Delhi: Sage Publications, Inc.

Ferrer, Belén Martínez, David Moreno Ruiz, Luis V. Amador, and Jim Orford. 2011. “School Victimization among Adolescents. An Analysis from an Ecological Perspective.” *Psychosocial Intervention* 20 (2): 149–60. https://doi.org/10.5093/in2011v20n2a3.

Fiset, John, Raghid Al Hajj, and John G. Vongas. 2017. “Workplace Ostracism Seen through the Lens of Power.” *Frontiers in Psychology* 8 (SEP): 1–19. https://doi.org/10.3389/fpsyg.2017.01528.

Fredericksen, Elizabeth D., and Suzanne McCorkle. 2013. “Explaining Organizational Responses to Workplace Aggression.” *Public Personnel Management* 42 (2): 223–38. https://doi.org/10.1177/0091026013487050.

Friedenberg, Joan. 2008. *The Anatomy of an Academic Mobbing.* Lewiston. NY: Edwin Mellen Press.

Friis, Camilla B., Lasse Suonperä Liebst, Richard Philpot, and Marie Rosenkrantz Lindegaard. 2020. “Ticket Inspectors in Action: Body-Worn Camera Analysis of Aggressive and Nonaggressive Passenger Encounters.” *Psychology of Violence* 10 (5): 483–92. https://doi.org/10.1037/vio0000276.

Galperin, Bella L. 2012. “Exploring the Nomological Network of Workplace Deviance: Developing and Validating a Measure of Constructive Deviance.” *Journal of Applied Social Psychology* 42 (12): 2988–3025. https://doi.org/10.1111/j.1559-1816.2012.00971.x.

Gee, J. P. 1996. *Social Linguistics and Literacies: Ideology in Discourses*. 2nd ed. London: Taylor & Francis.

Glambek, Mats, Anders Skogstad, and Ståle Valvatne Einarsen. 2020. “Does the Number of Perpetrators Matter? An Extension and Re‐analysis of Workplace Bullying as a Risk Factor for Exclusion from Working Life.” *Journal of Community & Applied Social Psychology* 1 (8): casp.2456. https://doi.org/10.1002/casp.2456.

Gonsalkorale, Karen, and Kipling D. Williams. 2007. “The KKK Won’t Let Me Play: Ostracism Even by a Despised Outgroup Hurts.” *European Journal of Social Psychology* 37 (6): 1176–86. https://doi.org/10.1002/ejsp.392.

González Trijueque, David, and José Luis Graña Gómez. 2010. “Workplace Bullying: Prevalence and Descriptive Analysis in a Multi-Occupational Sample.” *Psicothema* 21 (2): 288–93. http://www.psychologyinspain.com/content/full/2010/14003.pdf.

Goossens, F.A., T. Olthof, and P.H. Dekker. 2006. “New Participant Role Scales: Comparison between Various Criteria for Assigning Roles and Indications for Their Validity.” *Aggressive Behavior* 32 (4): 343–57. https://doi.org/10.1002/ab.20133.

Grandey, Alicia A., David N. Dickter, and Hock-Peng Sin. 2004. “The Customer Isnot Always Right: Customer Aggression and Emotion Regulation of Service Employees.” *Journal of Organizational Behavior* 25 (3): 397–418. https://doi.org/10.1002/job.252.

Grandey, Alicia A., Julie H. Kern, and Michael R. Frone. 2007. “Verbal Abuse from Outsiders versus Insiders: Comparing Frequency, Impact on Emotional Exhaustion, and the Role of Emotional Labor.” *Journal of Occupational Health Psychology* 12 (1): 63–79. https://doi.org/10.1037/1076-8998.12.1.63.

Griffin, Barbara. 2010. “Multilevel Relationships between Organizational-Level Incivility, Justice and Intention to Stay.” *Work & Stress* 24 (4): 309–23. https://doi.org/10.1080/02678373.2010.531186.

Gruys, Melissa L., and Paul R. Sackett. 2003. “Investigating the Dimensionality of Counterproductive Work Behavior.” *International Journal of Selection and Assessment* 11 (1): 30–42. https://doi.org/10.1111/1468-2389.00224.

Harris, L. C., and Emmanuel Ogbonna. 2006. “Service Sabotage: A Study of Antecedents and Consequences.” *Journal of the Academy of Marketing Science* 34 (4): 543–58. https://doi.org/10.1177/0092070306287324.

Haviland, Victoria S. 2008. “Things Get Glossed Over.” *Journal of Teacher Education* 59 (1): 40–54. https://doi.org/10.1177/0022487107310751.

Hershcovis, M. Sandy, and Julian Barling. 2010. “Comparing Victim Attributions and Outcomes for Workplace Aggression and Sexual Harassment.” *Journal of Applied Psychology* 95 (5): 874–88. https://doi.org/10.1037/a0020070.

Hershcovis, M. Sandy, and Tara C. Reich. 2013. “Integrating Workplace Aggression Research: Relational, Contextual, and Method Considerations.” *Journal of Organizational Behavior* 34 (S1): S26–42. https://doi.org/10.1002/job.1886.

Hoel, H, S I Giga, and M J Davidson. 2007. “Expectations and Realities of Student Nurses’ Experiences of Negative Behaviour and Bullying in Clinical Placement and the Influences of Socialization Processes.” *Health Services Management Research* 20 (4): 270–78. https://doi.org/10.1258/095148407782219049.

Hoel, Helge, Cary L. Cooper, and Brian Faragher. 2001. “The Experience of Bullying in Great Britain: The Impact of Organizational Status.” Edited by Cary L. Cooper. *European Journal of Work and Organizational Psychology* 10 (4): 443–65. https://doi.org/10.1080/13594320143000780.

Hoel, Helge, Lars Glasø, Jørn Hetland, Cary L. Cooper, and Ståle Einarsen. 2009. “Leadership Styles as Predictors of Self-Reported and Observed Workplace Bullying.” *British Journal of Management* 21 (2): 453–68. https://doi.org/10.1111/j.1467-8551.2009.00664.x.

Holroyd-Leduc, Jayna M., and Sharon E. Straus. 2018. “#MeToo and the Medical Profession.” *Canadian Medical Association Journal* 190 (33): E972–73. https://doi.org/10.1503/cmaj.181037.

Hubert, Adrienne B., and Marc van Veldhoven. 2001. “Risk Sectors for Undesirable Behaviour and Mobbing.” *European Journal of Work and Organizational Psychology* 10 (4): 415–24. https://doi.org/10.1080/13594320143000799.

Huitsing, Gijs, and René Veenstra. 2012. “Bullying in Classrooms: Participant Roles from a Social Network Perspective.” *Aggressive Behavior* 38 (6): 494–509. https://doi.org/10.1002/ab.21438.

Hutchinson, Marie. 2013. “Bullying as Workgroup Manipulation: A Model for Understanding Patterns of Victimization and Contagion within the Workgroup.” *Journal of Nursing Management* 21 (3): 563–71. https://doi.org/10.1111/j.1365-2834.2012.01390.x.

Injury Prevention Research Centre. 2001. “Workplace Violence: A Report to the Nation.” Iowa City, IA. http://www.public-health.uiowa.edu/iprc/.

Ireland, Jane. 2013. “Bullying among Prisoners: Innovations in Theory and Research.” In *Bullying among Prisoners: The Need for Innovation.*, edited by Jane L. Ireland, 2nd ed., 1–30. Cullompton: Willan. https://doi.org/10.4324/9781843925750.

Jonge, Jan de, and Maria C.W. Peeters. 2009. “Convergence of Self-Reports and Coworker Reports of Counterproductive Work Behavior: A Cross-Sectional Multi-Source Survey among Health Care Workers.” *International Journal of Nursing Studies* 46 (5): 699–707. https://doi.org/10.1016/j.ijnurstu.2008.12.010.

Kant, Leo, Anders Skogstad, Torbjørn Torsheim, and Ståle Einarsen. 2013. “Beware the Angry Leader: Trait Anger and Trait Anxiety as Predictors of Petty Tyranny.” *The Leadership Quarterly* 24 (1): 106–24. https://doi.org/10.1016/j.leaqua.2012.08.005.

Kavitha, S, and R Bhuvaneswari. 2016. “Impact of Social Media on Millenials- a Conceptual Study.” *Journal of Management Sciences and Technology* 4 (1): 80–86. https://www.apeejay.edu/aitsm/journal/docs/issue-oct-2016/ajmst040108.pdf.

Keashly, Loraleigh, and Steve Harvey. 2005. “Emotional Abuse in the Workplace.” In *Counterproductive Work Behavior: Investigations of Actors and Targets.*, 201–35. Washington: American Psychological Association. https://doi.org/10.1037/10893-009.

Lamertz, Kai, and Karl Aquino. 2004. “Social Power, Social Status and Perceptual Similarity of Workplace Victimization: A Social Network Analysis of Stratification.” *Human Relations* 57 (7): 795–822. https://doi.org/10.1177/0018726704045766.

LeBlanc, Manon Mireille, and E. Kevin Kelloway. 2002. “Predictors and Outcomes of Workplace Violence and Aggression.” *Journal of Applied Psychology* 87 (3): 444–53. https://doi.org/10.1037/0021-9010.87.3.444.

Leun, Joanne P. van der, and Maartje A.H. van der Woude. 2011. “Ethnic Profiling in the Netherlands? A Reflection on Expanding Preventive Powers, Ethnic Profiling and a Changing Social and Political Context.” *Policing and Society* 21 (4): 444–55. https://doi.org/10.1080/10439463.2011.610194.

Leymann, Heinz, and U Tallgren. 1989. “Undersökning Av Frekvensen of Vuxenmobbning Inom SSAB Med Ett Nytt Frågeformulär [Investigation of the Frequency of Adult Mobbing at Work within SSAB with a New Survey Questionnaire].” *Arbete, Människa, Miljö* 1: 3–12.

Leymann, Heinz, and Dieter Zapf. 1990. “Mobbing and Psyhological Terror at Workplaces’ Violence and Victims.” *European Journal* 5 (2): 22. https://www.mobbingportal.com/LeymannV&V1990(3).pdf.

Lim, Sandy, Lilia M. Cortina, and Vicki J. Magley. 2008. “Personal and Workgroup Incivility: Impact on Work and Health Outcomes.” *Journal of Applied Psychology* 93 (1): 95–107. https://doi.org/10.1037/0021-9010.93.1.95.

Lucas, Gale M., and James Friedrich. 2005. “Individual Differences in Workplace Deviance and Integrity as Predictors of Academic Dishonesty.” *Ethics & Behavior* 15 (1): 15–35. https://doi.org/10.1207/s15327019eb1501_2.

Macdonald, Grant, and Frank Sirotich. 2005. “Violence in the Social Work Workplace.” *International Social Work* 48 (6): 772–81. https://doi.org/10.1177/0020872805057087.

Maran, Daniela Acquadro, Silvia Bernardelli, and Antonella Varetto. 2018. “Mobbing (Bullying at Work) in Italy: Characteristics of Successful Court Cases.” *Journal of Injury and Violence Research* 10 (1): 17–24. https://doi.org/10.5249/jivr.v10i1.945.

McCarty, Megan K., Nicole E. Iannone, and Janice R. Kelly. 2014. “Stranger Danger: The Role of Perpetrator and Context in Moderating Reactions to Sexual Harassment.” *Sexuality & Culture* 18 (4): 739–58. https://doi.org/10.1007/s12119-013-9215-0.

McLindon, Elizabeth, Cathy Humphreys, and Kelsey Hegarty. 2018. “‘It Happens to Clinicians Too’: An Australian Prevalence Study of Intimate Partner and Family Violence against Health Professionals.” *BMC Women’s Health* 18 (1): 113. https://doi.org/10.1186/s12905-018-0588-y.

Merchant, James A, and John A Lundell. 2001. “Workplace Violence Intervention Research Workshop, April 5-7, Washington, DC.” *American Journal of Preventive Medicine* 20 (2): 135–40. https://doi.org/10.1016/S0749-3797(00)00289-0.

Mintzberg, Henry. 1983. *Power in and around Organizations*. Prentice Hall.

Mintzberg, Henry, Dror Etzion, and Mantere Saku. 2019. “Summary for Policymakers.” In *Climate Change 2013 - The Physical Science Basis*, edited by Intergovernmental Panel on Climate Change, 53:1–30. Cambridge: Cambridge University Press. https://doi.org/10.1017/CBO9781107415324.004.

National Institute for Occupational Safety and Health. 2013. “NIOSH-WPVHC-Workplace Violence Types.” *Workplace Violence Prevention for Nurses*. https://wwwn.cdc.gov/wpvhc/Course.aspx/Slide/Unit1_5.

Nauta, Aukje, Carsten K.W. De Dreu, and Taco Van Der Vaart. 2002. “Social Value Orientation, Organizational Goal Concerns and Interdepartmental Problem-Solving Behavior.” *Journal of Organizational Behavior*. https://doi.org/10.1002/job.136.

Neall, Annabelle M., and Michelle R. Tuckey. 2014. “A Methodological Review of Research on the Antecedents and Consequences of Workplace Harassment.” *Journal of Occupational and Organizational Psychology* 87 (2): 225–57. https://doi.org/10.1111/joop.12059.

Nielsen, Morten Birkeland, and Ståle Einarsen. 2012. “Outcomes of Exposure to Workplace Bullying: A Meta-Analytic Review.” *Work & Stress* 26 (4): 309–32. https://doi.org/10.1080/02678373.2012.734709.

Nissen, Alexander, Marianne Bang Hansen, Morten Birkeland Nielsen, Stein Knardahl, and Trond Heir. 2019. “Employee Safety Perception Following Workplace Terrorism: A Longitudinal Study.” *European Journal of Psychotraumatology* 10 (1): 1478584. https://doi.org/10.1080/20008198.2018.1478584.

O’Boyle, Ernest H., Donelson R. Forsyth, and Allison S. O’Boyle. 2011. “Bad Apples or Bad Barrels: An Examination of Group- and Organizational-Level Effects in the Study of Counterproductive Work Behavior.” *Group & Organization Management* 36 (1): 39–69. https://doi.org/10.1177/1059601110390998.

Okechukwu, Cassandra A., Kerry Souza, Kelly D. Davis, and A. Butch de Castro. 2014. “Discrimination, Harassment, Abuse, and Bullying in the Workplace: Contribution of Workplace Injustice to Occupational Health Disparities.” *American Journal of Industrial Medicine* 57 (5): 573–86. https://doi.org/10.1002/ajim.22221.

Olthof, Tjeert, and Frits A. Goossens. 2008. “Bullying and the Need to Belong: Early Adolescents’ Bullying-Related Behavior and the Acceptance They Desire and Receive from Particular Classmates.” *Social Development* 17 (1): 24–46. https://doi.org/10.1111/j.1467-9507.2007.00413.x.

Öztunç, Gürsel. 2006. “Examination of Incidents of Workplace Verbal Abuse against Nurses.” *Journal of Nursing Care Quality* 21 (4): 360–65. https://doi.org/10.1097/00001786-200610000-00014.

Parzefall, Marjo-Riitta, and Denise M. Salin. 2010. “Perceptions of and Reactions to Workplace Bullying: A Social Exchange Perspective.” *Human Relations* 63 (6): 761–80. https://doi.org/10.1177/0018726709345043.

Pattani, Reena, Shiphra Ginsburg, Alekhya Mascarenhas Johnson, Julia E. Moore, Sabrina Jassemi, and Sharon E. Straus. 2018. “Organizational Factors Contributing to Incivility at an Academic Medical Center and Systems-Based Solutions.” *Academic Medicine* 93 (10): 1569–75. https://doi.org/10.1097/ACM.0000000000002310.

Peng, Li, Kai Xing, Hong Qiao, Huiying Fang, Hongkun Ma, Mingli Jiao, Yanhua Hao, et al. 2018. “Psychological Violence against General Practitioners and Nurses in Chinese Township Hospitals: Incidence and Implications.” *Health and Quality of Life Outcomes* 16 (1): 117. https://doi.org/10.1186/s12955-018-0940-9.

Pheko, Mpho M. 2018. “Auto Ethnography and Cognitive Adaptation: Two Powerful Buffers against the Negative Consequences of Workplace Bullying and Academic Mobbing.” *International Journal of Qualitative Studies on Health and Well-Being* 13 (1). https://doi.org/10.1080/17482631.2018.1459134.

Pinto, Jonathan. 2014. “Expanding the Content Domain of Workplace Aggression: A Three-Level Aggressor-Target Taxonomy.” *International Journal of Management Reviews* 16 (3): 290–313. https://doi.org/10.1111/ijmr.12021.

Reyns, Bradford W., Billy Henson, and Bonnie S. Fisher. 2012. “Stalking in the Twilight Zone: Extent of Cyberstalking Victimization and Offending among College Students.” *Deviant Behavior* 33 (1): 1–25. https://doi.org/10.1080/01639625.2010.538364.

Ringstad, Robin. 2005. “Conflict in the Workplace: Social Workers as Victims and Perpetrators.” *Social Work* 50 (4): 305–13. https://doi.org/10.1093/sw/50.4.305.

Rizvi, Syed Tahir, Barry A. Friedman, and Rauf I. Azam. 2017. “Overall Injustice, Workplace Deviance and Turnover Intention among Educators and Supporters.” *The BRC Academy Journal of Business* 7 (1): 45–71. https://doi.org/10.15239/j.brcacadjb.2017.07.01.ja03.

Robinson, Sandra L., and Rebecca J. Bennett. 1995. “A Typology of Deviant Workplace Behaviors: A Multidimensional Scaling Study.” *Academy of Management Journal* 38 (2): 555–72. https://doi.org/10.5465/256693.

Russo, Andrea, Ranko Milić, Bojana Knežević, Rosanda Mulić, and Jadranka Mustajbegović. 2008. “Harassment in Workplace among School Teachers: Development of Survey.” *Croatian Medical Journal* 49 (4): 545–52. https://doi.org/10.3325/cmj.2008.4.545.

Salmivalli, Christina, Kirsti Lagerspetz, Kaj Björkqvist, Karin Österman, and Ari Kaukiainen. 1998. “Bullying as a Group Process: Participant Roles and Their Relations to Social Status within the Group.” *Aggressive Behavior* 22 (1): 1–15. https://doi.org/10.1002/(SICI)1098-2337(1996)22:1<1::AID-AB1>3.0.CO;2-T.

Saunders, Peter, and Melissa Wong. 2009. “Still Doing It Tough : An Update on Deprivation and Social Exclusion among Welfare Service Clients.” *Social Policy*, no. July. http://www.sprc.unsw.edu.au/media/File/Report7_09_Still_doing_it_tough.pdf.

Scalora, Mario J., David O’neil Washington, Thomas Casady, and Sarah P. Newell. 2003. “Nonfatal Workplace Violence Risk Factors.” *Journal of Interpersonal Violence* 18 (3): 310–27. https://doi.org/10.1177/0886260502250092.

Schat, Aaron C.H., and Kevin E Kelloway. 2005. “Workplace Aggression.” In *Handbook of Work Stress*, edited by Julian Barling, E Kevin Kelloway, and Michael R. Frone, 189–218. Thousand Oaks: Sage.

Schilpzand, Pauline, Irene E. De Pater, and Amir Erez. 2016. “Workplace Incivility: A Review of the Literature and Agenda for Future Research.” *Journal of Organizational Behavior* 37 (February): S57–88. https://doi.org/10.1002/job.1976.

Sheahan, Matthew, and Philip Smith. 2003. “Deviance and Marginal Occupations: The Case of Taxi Drivers.” *Deviant Behavior* 24 (5): 449–66. https://doi.org/10.1080/713840244.

Sijbers, Rob, Wouter De Wit, Daan Fettelaar, and Ton Mooij. 2014. “Sociale Veiligheid in En Rond Scholen. Primair (Speciaal) Onderwijs 2010-2014; Voortgezet (Speciaal) Onderwijs 2006-2014.( Social Safety in and around Schools. Primary (Special) Education 2010-2014; Secundairy (Special) Education 2006-2014).” Nijmegen.

Sliter, Michael, Steve Jex, Katherine Wolford, and Joanne McInnerney. 2010. “How Rude! Emotional Labor as a Mediator between Customer Incivility and Employee Outcomes.” *Journal of Occupational Health Psychology* 15 (4): 468–81. https://doi.org/10.1037/a0020723.

Sliter, Michael T., Shuang Yueh Pui, Katherine A. Sliter, and Steve M. Jex. 2011. “The Differential Effects of Interpersonal Conflict from Customers and Coworkers: Trait Anger as a Moderator.” *Journal of Occupational Health Psychology* 16 (4): 424–40. https://doi.org/10.1037/a0023874.

Snyder, Joseph. 2012. “How Does Bullying Relate to Elder Abuse?” *Temple Political & Civil Rights Law Review* 22: 386. http://heinonline.org/HOL/Page?handle=hein.journals/ tempcr22&id=412&div=&collection=.

Spector, Paul E., and Suzy Fox. 2005. “The Stressor-Emotion Model of Counterproductive Work Behavior.” In *Counterproductive Work Behavior: Investigations of Actors and Targets.*, edited by Suzy Fox and Paul E Spector, 151–74. Washington: American Psychological Association. https://doi.org/10.1037/10893-007.

Sperry, Len. 2009. “Workplace Mobbing and Bullying: A Consulting Psychology Perspective and Overview.” *Consulting Psychology Journal: Practice and Research* 61 (3): 165–68. https://doi.org/10.1037/a0016936.

Takaki, Jiro, Toshiyo Taniguchi, and Kumi Hirokawa. 2013. “Associations of Workplace Bullying and Harassment with Pain.” *International Journal of Environmental Research and Public Health* 10 (10): 4560–70. https://doi.org/10.3390/ijerph10104560.

Teymourzadeh, Ehsan, Arash Rashidian, Mohammad Arab, Ali Akbari-Sari, and Seyyed Mostafa Hakimzadeh. 2014. “Nurses Exposure to Workplace Violence in a Large Teaching Hospital in Iran.” *International Journal of Health Policy and Management* 3 (6): 301–5. https://doi.org/10.15171/ijhpm.2014.98.

Török, Eszter, Åse Marie Hansen, Matias Brødsgaard Grynderup, Anne Helene Garde, Annie Høgh, and Kirsten Nabe-Nielsen. 2016. “The Association between Workplace Bullying and Depressive Symptoms: The Role of the Perpetrator.” *BMC Public Health* 16 (1): 1–10. https://doi.org/10.1186/s12889-016-3657-x.

Vigoda, Eran. 2001. “Reactions to Organizational Politics: A Cross-Cultural Examination in Israel and Britain.” *Human Relations* 54 (11): 1483–1518. https://doi.org/10.1177/00187267015411004.

Walsh, Benjamin M., Allison Burrus, Dana Kabat-Farr, Alyssa K. McGonagle, Elizabeth Call, Allie McIntire, and Frances C. Shen. 2020. “Living a Calling and Perceived Work Ability in Domestic Violence Services.” *Journal of Counseling Psychology* 67 (2): 241–50. https://doi.org/10.1037/cou0000387.

Williams, Kipling D. 2007. “Ostracism.” *Annual Review of Psychology* 58 (1): 425–52. https://doi.org/10.1146/annurev.psych.58.110405.085641.

Wilson, Nicole L., and Camilla M. Holmvall. 2013. “The Development and Validation of the Incivility from Customers Scale.” *Journal of Occupational Health Psychology* 18 (3): 310–26. https://doi.org/10.1037/a0032753.

Zahlquist, Lena, Jørn Hetland, Anders Skogstad, Arnold B. Bakker, and Ståle Valvatne Einarsen. 2019. “Job Demands as Risk Factors of Exposure to Bullying at Work: The Moderating Role of Team-Level Conflict Management Climate.” *Frontiers in Psychology* 10 (September): 1–11. https://doi.org/10.3389/fpsyg.2019.02017.

Zapf, D, S Einarsen, H Hoel, and M Vartia. 2003. “Empirical Findings on Bullying in the Workplace.” In *Bullying and Emotional Abuse in the Workplace: International Perspectives in Research and Practice.*, edited by S. Einarsen, H. Hoel, D Zapf, and C. Cooper, 103–26. London: Taylor & Francis.

Zhou, Jing, and Gerald R. Ferris. 1995. “The Dimensions and Consequences of Organizational Politics Perceptions: A Confirmatory Analysis1.” *Journal of Applied Social Psychology* 25 (19): 1747–64. https://doi.org/10.1111/j.1559-1816.1995.tb01816.x.
